# Response to FOLFIRINOX by gender in patients with metastatic pancreatic cancer: Results from the PRODIGE 4/ ACCORD 11 randomized trial

**DOI:** 10.1371/journal.pone.0183288

**Published:** 2017-09-20

**Authors:** Aurélien Lambert, Marta Jarlier, Sophie Gourgou Bourgade, Thierry Conroy

**Affiliations:** 1 Department of medical oncology, Institut de Cancérologie de Lorraine, Vandoeuvre-lès-Nancy, France; 2 Biostatistics unit, Institut régional du cancer de Montpellier, Parc Euromédecine, Montpellier, France; German Cancer Research Center (DKFZ), GERMANY

## Abstract

**Background:**

Hohla et al. suggested that female gender could positively predict response to FOLFIRINOX in patients with advanced pancreatic cancer. In this study, we explored the response to the FOLFIRINOX regimen by gender within the trial PRODIGE4/ACCORD 11.

**Patients and methods:**

Data were described by gender, both in FOLFIRINOX group and in the intention-to-treat population of the trial. The relative effect of gender (females in comparison to males) on overall survival (OS) and progression-free survival was estimated by using a Cox proportional hazard model and was presented with the Hazard Ratio and their 95% confidence interval. The analysis of prognostic factors of OS included also: age (older than 65 years), ECOG performance status, primary tumor location, synchronous metastases, number of metastatic sites, hepatic metastasis, pulmonary metastases, lymph node metastases, level of Albumin and level of serum carbohydrate antigen 19–9 and three domains from the EORTC Quality of Life QLQC-30 questionnaire.

**Results:**

The FOLFIRINOX group (*N* = 171 patients) included 106 women (62%) and 65 men. No significant differences were observed between genders regarding demographic and clinical parameters, excepted for lymph nodes metastasis (17% and 35% in women and men respectively; *p* = 0.012). Median OS was longer for females as compared to males in FOLFIRINOX group (13.1 versus 10.3 months respectively; HR = 0.73; 95% CI, 0.51–1.06). Similarly, median PFS was superior (7.2 versus 5.9 months; HR = 0.79; 95% CI, 0.57–1.10). Nevertheless, in both cases, the differences were not statistically significant (p = 0.10 et p = 0.169, respectively).

**Conclusions:**

In this study, the overall survival and progression-free survival rates were not significantly higher for females than for males in FOLFIRINOX group (HR = 0.73; 95% CI, 0.51–1.06 and HR = 0.79; 95% CI, 0.57–1.10 respectively). Even if the percentage of patients with lymph node metastasis is higher for males than for females, the interaction between gender and lymph node metastasis was non-significant. Our exploratory analysis did not permit to definitively conclude about a possible effect of gender on the prognosis of patients under FOLFIRINOX. This subject deserves further evaluation.

**Trial registration:**

ClinicalTrials.gov number: NCT00112658

**Key message:**

Our analysis suggests that FOLFIRINOX, as first-line option for patients with metastatic pancreatic cancer who are younger than 76 years and who have a good performance status (ECOG 0 or 1), no cardiac ischemia and normal or nearly normal bilirubin levels, is beneficial, but not particularly in female patients.

## Introduction

The combination of oxaliplatin, irinotecan and fluorouracil, known as FOLFIRINOX, emerged as a first-line treatment option for selected patients with metastatic pancreatic cancer. The pivotal PRODIGE 4/ACCORD 11 randomized trial comparing FOLFIRINOX with single-agent gemcitabine demonstrated superior median survival and longer time without degradation of quality of life [1]. The objective response rate was 31,6% with FOLFIRINOX versus 9,4% with Gemcitabine (*p*<0.001) [1]. In their study, Hohla et al. suggested that female gender could positively predict response to FOLFIRINOX in patients with advanced pancreatic cancer [2]. In this secondary analysis, we explored the response to the FOLFIRINOX regimen by gender within the trial PRODIGE 4/ACCORD 11.

## Methods

### Patients

**Fig 1:**
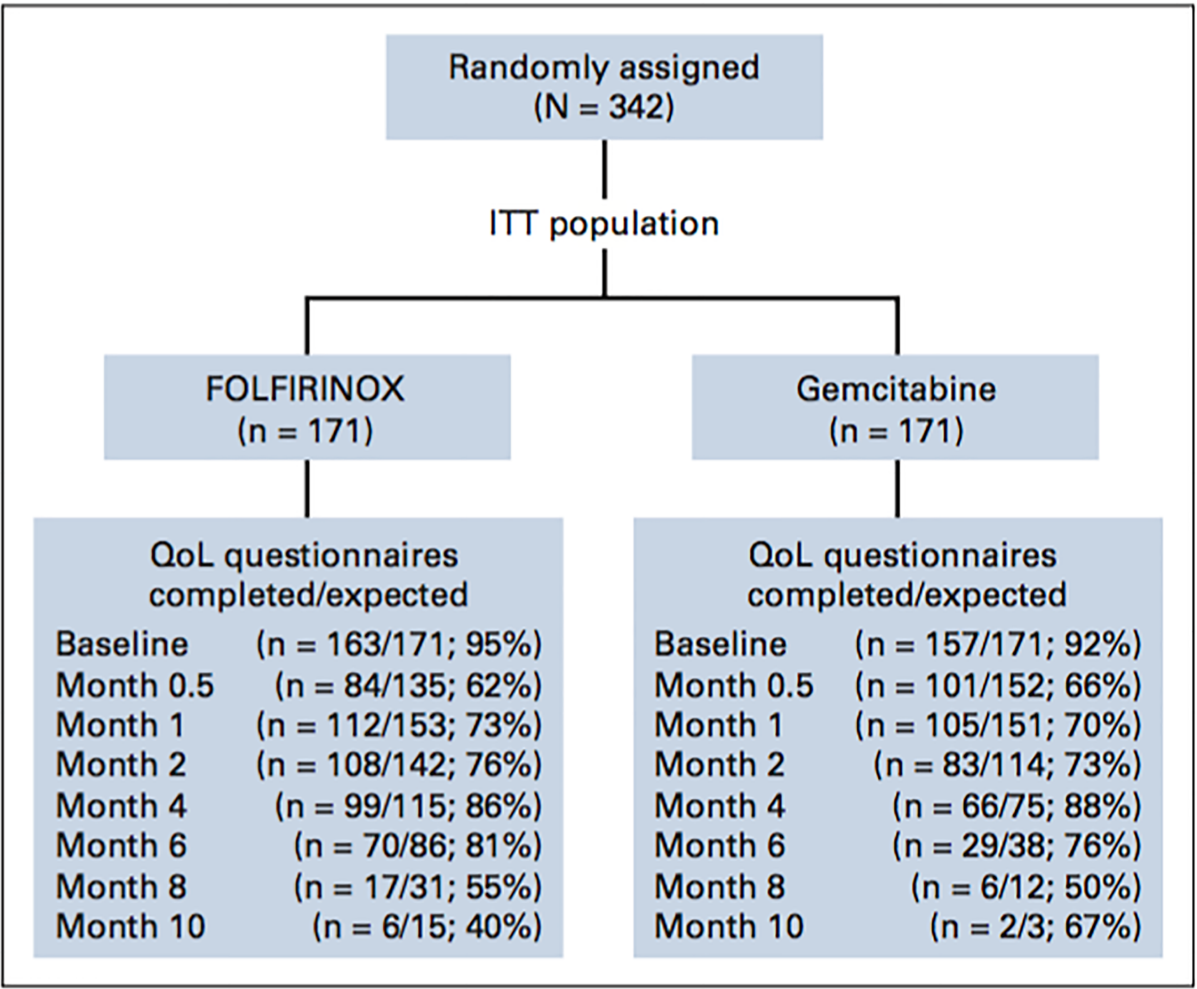
CONSORT diagram for European organisation for the research of cancer quality of life questionnaires C30 completed for the total number of eligible.

Between December 2005 and October 2009, a total of 342 patients were enrolled in the study. The database was closed for final analysis on April 16, 2010. The intention-to-treat (ITT) population included 171 patients in each group, and the safety population (all patients who received treatment) included 167 patients in the FOLFIRINOX group and 169 patients in the gemcitabine group (Fig 1). Data were described by gender, both in FOLFIRINOX group and in the ITT population of the trial.

### Study design

This multicenter, randomized, phase 2–3 trial was conducted at 15 centers during phase 2 and expanded to 48 centers during phase 3. Patients were randomly assigned to receive FOLFIRINOX or gemcitabine within 1 week after enrollment. Randomization was performed centrally in a 1:1 ratio with stratification according to center, performance status (0 vs. 1), and primary tumor localization (the head vs. the body or tail of the pancreas).

The study was approved by the Lorraine ethics committee. All patients provided written informed consent. An independent data and safety monitoring committee supervised the collation of efficacy and safety data. The trial was conducted according the Declaration of Helsinki, the Good Clinical Practice guidelines of the International Conference on Harmonization, and relevant French and European laws and directives. Data were collected at the headquarters of the French anticancer centers (Unicancer, the study sponsor) and analyzed by the statistician, who vouches for the accuracy of the data. Oxaliplatin and irinotecan were donated by Sanofi-Aventis and Pfizer, respectively; these drug manufacturers had no role in the design of the study, in the accrual or analysis of the data, or in the preparation of the manuscript.

### Statistical analysis

Qualitative variables were compared using the chi-square test. Overall survival (OS) and progression-free survival (PFS) were estimated by using the Kaplan-Meier method and comparisons were performed using the Log-rank test.

The relative effect of gender (females in comparison to males) on OS and PFS was estimated by using a Cox proportional hazard model and was presented with the Hazard Ratio (HR), their 95% confidence interval (95% CI) and the associated p-value. Univariate Cox analysis to determine baseline prognostic factors for OS included also clinical baseline variables previously studied in the original publication (1) (age older than 65 years, ECOG performance status, primary tumor location, synchronous metastases, number of metastatic sites, hepatic metastasis, pulmonary metastases, lymph node metastases, level of Albumin and level of serum carbohydrate antigen 19–9 (CA19-9)). Albumin and CA19-9 range levels were defined in Conroy et al[1]. Moreover, three domains (Physical Function, constipation and Dyspnea) from the EORTC Quality of Life QLQC-30 questionnaire previously found to be significant [3] were also evaluated.

Based on p-values (selection criteria was fixed at p ≤ 0.20) in univariate analysis, a first multivariable analysis was performed using a Cox proportional hazard model including only clinical variables as well as a term testing for the interaction between gender and lymph node metastases. In a second time, significant clinical variables in the first model were kept (a more stringent selection criteria at p < 0.10 was considered) and quality of life dimensions were added to the model. The details concerning variables included in the analyses are presented in two dedicated tables.

Data were analyzed with the software STATA, version 13.

## Results

Demographic and clinical variables stratified by gender and treatment arms are showed in Table 1. The FOLFIRINOX group (*N* = 171 patients) included 106 women (62%) and 65 men. No significant differences were observed between genders regarding baseline variables, excepted for lymph nodes metastasis (17% and 35% in women and men respectively; *p* = 0.012). This difference was also observed in the ITT population (62 lymph node metastasis in males (29.5%) and 25 in females (19.2%); p = 0.035).

**Table 1:** Demographic and clinical characteristics by gender and treatment arms.

	FOLFIRINOX arm (N = 171)			Gemcitabine arm (N = 171)			
	Male	Female	p-value	Male	Female		p-value
	(N = 106)	(N = 65)		(N = 105)	(N = 66)		
Age—yr			0.291				0.607
Median	60	62		61		61	
Range	31–74	25–76		40–75		34–75	
ECOG performance status score—no. (%)			0.134				0.456
0	44 (41.5)	19 (29.2)		37 (35.2)		27 (40.9)	
1	62 (58.5)	45 (69.2)		68 (64.8)		39 (59.1)	
2	0 (0.0)	1 (1.5)		0 (0.0)		0 (0.0)	
Pancreatic tumor location—no. (%)			0.763				0.996
Head	43 (40.6)	24 (37.4)		38 (36.2)		25 (37.9)	
Body	33 (31.1)	20 (32.1)		36 (34.3)		22 (33.3)	
Tail	27 (25.5)	18 (26.7)		28 (26.7)		17 (25.8)	
Multicentric	3 (2.8)	3 (4.6)		3 (2.9)		2 (3.0)	
Metastatic sites involved			0.551				0.862
Median	2	2		2		2	
Range	1–6	1–5		1–6		1–4	
Measurable metastatic site—no. (%)							
Liver	92 (87.6)	57 (89.1)	0.778	93 (88.6)		57 (86.4)	0.669
Pancreas	57 (54.3)	32 (50.0)	0.588	55 (52.4)		36 (54.5)	0.782
Lymph node	37 (35.2)	11 (17.2)	0.012*	25 (23.8)		14 (21.2)	0.694
Lung	18 (17.1)	15 (23.4)	0.317	30 (28.6)		19 (28.8)	0.976
Peritoneal	16 (15.2)	17 (26.6)	0.072	20 (19.0)		12 (18.2)	0.888
Other	13 (12.4)	5 (7.8)	0.350	17 (16.2)		12 (18.2)	0.736

The objective response rate for females and males in the FOLFIRINOX group were of 36.9% and 28.3% respectively (*p* = 0.239), whereas the disease control rate was of 71.7% versus 67.7% for males and females respectively (*p* = 0.578). Similar results were observed in the ITT population of the study: objective response rate of 24.4% in females and 18.0% in males (p = 0.153); disease control rate of 60.7% and 60.3% in males and females respectively (p = 0.948).

**Fig 2:**
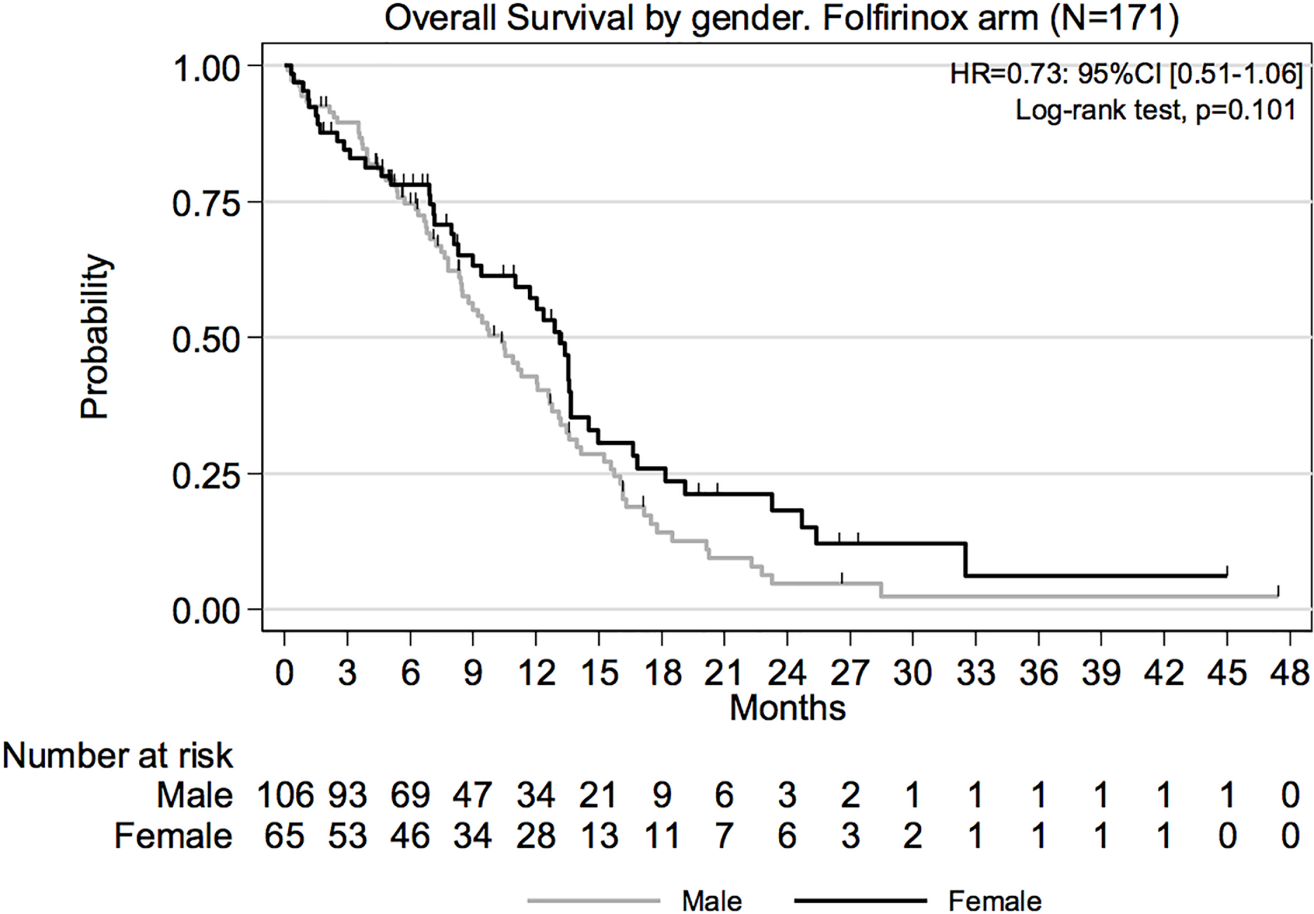
Overall survival by gender, Folfirinox arm.

**Fig 3:**
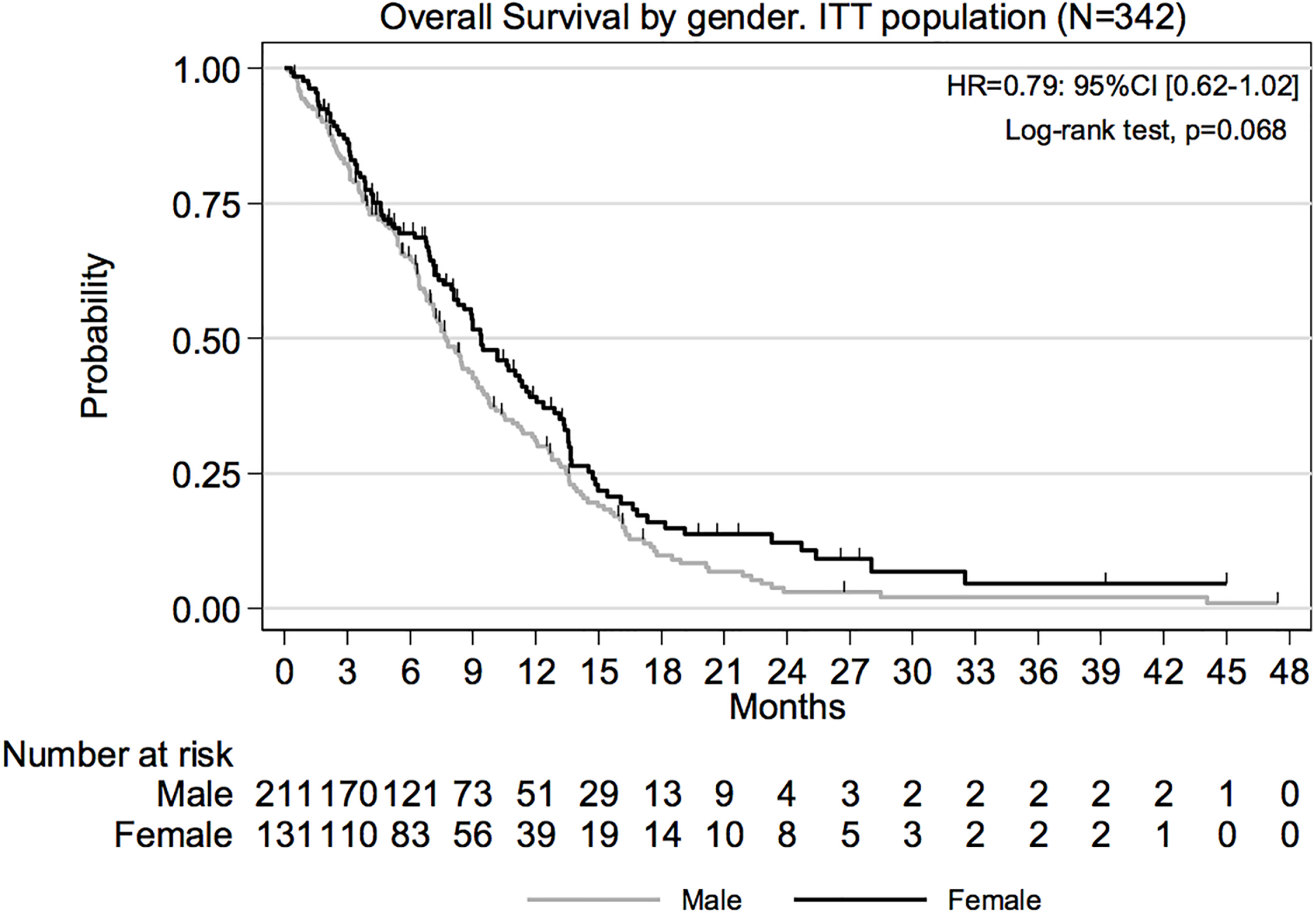
Overall survival by gender, ITT population.

Median OS was longer for females as compared to males in FOLFIRINOX group (13.1 versus 10.3 months respectively; HR = 0.73; 95% CI, 0.51–1.06) (Fig 2). Similarly, median PFS was superior (7.2 versus 5.9 months; HR = 0.79; 95% CI, 0.57–1.10). However, even if the observed differences were numerically higher, they were not statistically significant (p-values associated with HR were 0.101 and 0.169 respectively). Gender effect on OS (Fig 3) and PFS were also evaluated over the ITT population (Table 2). The HR for OS was estimated at 0.79 (95% CI 0.62–1.02; p = 0.068) in ITT population and 0.80 (95% CI (0.57–1.11; p = 0.182) in Gemcitabine arm.

**Table 2:** Overall survival and progression-free survival by gender and treatment arm.

	FOLFIRINOX arm (N = 171)		
	Male (n = 106)	Female (n = 65)	
Deaths	81 (76.4)	45 (69.2)	
**OS**			
Median OS, months (95%CI)	10.3 (8.4–12.6)	13.1 (9.0–13.7)	
1-year OS, % (95%CI)	42.8% (32.3–52.8)	57.2% (43.30–68.9)	
HR (95%CI)	0.73 (0.51–1.06)		
p-value^1^	0.101		
**PFS**			
Median PFS, months (95%CI)	5.9 (4.2–7.1)	7.2 (5.7–8.6)	
HR (95%CI)	0.79 (0.57–1.10)		
p-value	0.169		
	Gemcitabine arm (N = 171)		
	Male (n = 105)		Female (n = 66)
Deaths	92 (87.6)		55 (83.3)
**OS**			
Median OS, months (95%CI)	6.4 (5.4–7.2)		7.6 (5.3–9.4)
1-year OS, % (95%CI)	19.7% (12.2–28.5)		22.0% (12.3–33.5)
HR (95%CI)	0.80 (0.57–1.11)		
p-value	0.182		
**PFS**			
Median PFS, months (95%CI)	2.96 (2.04–3.65)		3.48 (1.91–4.11)
HR (95%CI)	0.98 (0.71–1.34)		
p-value	0.876		
	ITT trial population (N = 342)		
	Male (n = 211)	Female (n = 131)	
Deaths	173 (82.0)	100 (76.3)	
**OS**			
Median OS, months (95%CI)	7.7 (6.8–9.0)	9.4 (8.0–11.3)	
1-year OS, % (95%CI)	31.1% (24.5–38.0)	39.1% (30.1–48.0)	
HR (95%CI)	0.79 (0.62–1.02)		
p-value	0.068		
**PFS**			
Median PFS, months (95%CI)	3.9 (3.5–4.9)	4.3 (3.6–5.7)	
HR (95%CI)	0.87 (0.69–1.10)		
p-value	0.245		

^1^ p-value corresponds to HR

The results of the univariate Cox analysis in FOLFIRINOX arm is showed in Table 3. From the initially fourteen factors under study, and, based on p-values ≤ 0.20, eight clinical variables and the three QLQ-C30 domains were considered to the inclusion in the multivariable analysis. Variables (with their associated HR and p-value at univariate Cox analysis) were the following: gender (HR = 0.73, p = 0.101), ECOG performance status (HR = 1.43, p = 0.058); primary tumor location (HR = 1.38, p = 0.086); level of albumin (HR = 1.57, p = 0.024), synchronous metastases (HR = 2.81, p = 0.002); number of metastatic sites (HR = 1.15, p = 0.125); hepatic metastases (HR = 2.19, p = 0.014); CA19-9 level (HR = 1.47, p = 0.049), physical function (HR = 0.98, p<0.001), constipation (HR = 1.01, p = 0.003), and dyspnea (HR = 1.01, p = 0.001). Additionally, in order to adjust for the presence of lymph node metastases, this variable was also considered (HR = 0.98, p = 0.900).

**Table 3:** Prognostic factors for overall survival in FOLFIRINOX arm. Univariate analysis.

Variable	HR	IC95%	p-value
**Gender**			
Male	1.00		
Female	0.73	[0.51–1.06]	0.101
**Age (years)**			
≤ 65 yr	1.00		
>65 yr	0.95	[0.64–1.41]	0.795
**ECOG performance status**			
0	1		
1	1.43	[0.99–2.08]	0.058
**Primary tumor location**			
Head of pancreas	1		
Other	1.38	[0.96–1.98]	0.086
**Level of Albumin**			
Normal	1.00		
Abnormal	1.57	[1.06–2.33]	0.024
**Metastases**			
Metachronous	1.00		
Synchronous	2.81	[1.45–5.45]	0.002
**No. of metastatic sites**	1.15	[0.96–1.37]	0.125
**Hepatic metastases**			
No	1.00		
Yes	2.19	[1.18–4.08]	0.014
**Pulmonary metastases**			
No	1.00		
Yes	0.91	[0.58–1.44]	0.696
**Lymph node metastases**			
No	1		
Yes	0.98	[0.66–1.45]	0.900
**Level of CA 19–9**			
Normal	1.00		
Abnormal	1.55	[0.89–2.72]	0.123
**Quality of Life domains at baseline:**			
**Physical Function**	0.98	[0.97–0.99]	<0.001
**Constipation**	1.01	[1.00–1.01]	0.003
**Dyspnea**	1.01	[1.00–1.02]	0.001

First, a multivariable Cox model was performed including nine clinical variables (that is, the eight clinical variables mentioned above, plus the variable: presence of lymph node metastases) as well as a term testing for the interaction between gender and lymph node metastases (Table 4). This model suggest that females would be associated with a better prognosis than males (HR = 0.53; 95% CI, 0.32–0.88; p = 0.015); moreover, a non-significant interaction between gender and lymph node metastases was observed (HR = 1.90; 95% CI, 0.66–5.49; p = 0.234).

**Table 4:** Prognostic factors for overall survival in FOLFIRINOX arm. Multivariable analysis.

**Model with clinical variables and an interaction term (n = 133).**			
**Variable**	**HR**	**IC95%**	**p-value**
**Gender**			
Male	1.00		
Female	0.53	[0.32–0.88]	0.015
**ECOG performance status**			
0	1		
1	1.52	[0.98–2.36]	0.063
**Primary tumor location**			
Head of pancreas	1		
Other	1.30	[0.85–1.99]	0.233
**Level of Albumin**			
Normal	1.00		
Abnormal	1.93	[1.26–2.97]	0.003
**Metastases**			
Metachronous	1.00		
Synchronous	2.14	[0.98–4.70]	0.056
**No. of metastatic sites**	1.00	[0.78–1.30]	0.964
**Hepatic metastases**			
No	1.00		
Yes	1.66	[0.84–3.31]	0.147
**Level of CA 19–9**			
Normal	1.00		
Abnormal	1.90	[0.95–3.80]	0.069
**Lymph node metastases**			
No	1		
Yes	0.33	[0.08–1.33]	0.118
**Interaction gender/Lymph node metastases**	1.90	[0.66–5.49]	0.234
**Model with clinical variables (selected if p<0.10) and quality of life dimensions (n = 127).**			
**Variable**	**HR**	**IC95%**	**p-value**
**Gender**			
Male	1.00		
Female	0.65	[0.41–1.04]	0.073
**Level of Albumin**			
Normal	1.00		
Abnormal	1.37	[0.90–2.10]	0.146
**Metastases**			
Metachronous	1.00		
Synchronous	1.44	[0.65–3.20]	0.368
**Level of CA 19–9**			
Normal	1.00		
Abnormal	2.73	[1.29–5.80]	0.009
**Quality of Life domains at baseline:**			
Physical Function	0.98	[0.97–0.99]	0.001
Constipation	1.01	[1.00–1.01]	0.035
Dyspnea	1.01	[1.00–1.02]	0.043

Therefore, significant clinical variables were kept using a more stringent selection criteria (at p < 0.10) and quality of life dimensions were added, for a second multivariable Cox model (Table 4). After the addition of quality of life dimensions, the effect of gender is diminished (HR = 0.65; 95% CI, 0.41–1.04; p = 0.073). In this model, an abnormal CA19-9 level was associated with a worse prognosis (HR = 2.73; 95% CI, 1.29–5.80; p = 0.009); with respect to quality of life domains, even if the p-values associated to HR are under the significance level (0.05), HR remain around 1: a higher score of dyspnea (HR = 1.01; 95% CI, 1.00–1.02; p = 0.043) and constipation (HR = 1.01; 95% CI, 1.00–1.01; p = 0.035) were associated with a worse prognosis; whereas a higher physical function score was associated with a better prognosis (HR = 0.98; 95% CI, 0.97–0.99; p = 0.001).

## Discussion

In this study, despite the non-significant differences observed in the univariate analysis, the OS and PFS rates were higher for females than for males in FOLFIRINOX group (HR = 0.73 and HR = 0.79 respectively). When the ITT population was considered, a survival benefit for females was also suggested (HR = 0.79). One of the major biases is that the percentage of patients with lymph node metastasis is higher for males than for females. It could be one explanation of the differences between genders for the OS and the PFS, nevertheless a non-significant interaction between gender and lymph node metastases was showed by the model.

In the MPACT study comparing nab-paclitaxel plus gemcitabine to gemcitabine alone, age > 65 years, poorer Karnofsky performance status score, presence of liver metastasis, and higher number of metastatic sites were associated with an increased risk of death [4]. Gender was not included as a variable in the analysis, but a trend to a median superior survival in female was reported: 9.0 months (8.05–10.48) for female patients versus 8.1 months (6.93–9.30) for males in the combination arm, and 7.2 months (6.34–9.03) for gemcitabine alone in female patients versus 6.2 (5.45–6.97) for males.

It is important to highlight that all analysis conducted were not previously planned into the study protocol and were conducted only in an exploratory manner. The study has therefore several limitations inherent to unplanned subgroup analysis; moreover, multiplicity has not been taken into account. Finally, the limited sample size is one of the major limitation of this analysis by definition.

Our exploratory analysis did not permit to definitively conclude about a possible effect of gender on the prognosis of patients under FOLFIRINOX. Even if some results could numerically suggest a trend for a better prognosis in female patients with FOLFIRINOX as first-line option for patients with metastatic pancreatic cancer who are younger than 76 years and who have a good performance status (ECOG 0 or 1), no cardiac ischemia and normal or nearly normal bilirubin levels, no statistically significant differences between genders were found. This subject deserves further evaluation.

## Supporting information

S1 File PRODIGE 4/ ACCORD 11 randomized trial protocol. Including the statistical analysis plan, from NEJM.org.
(PDF)

S2 File CONSORT 2010. Checklist of information to include when reporting a randomized trial.
(PDF)
