# Supplementary material for: Response to FOLFIRINOX by gender in patients with metastatic pancreatic cancer: Results from the PRODIGE 4/ ACCORD 11 randomized trial
Source: PLoS One. 2017 Sep 20;12(9):e0183288. doi: 10.1371/journal.pone.0183288 (PMC5606928; doi:10.1371/journal.pone.0183288)
Supplement: S1 File — Including the statistical analysis plan, from NEJM.org. (PDF) [file pone.0183288.s001.pdf]

Protocol

This trial protocol has been provided by the authors to give readers additional information about their work.

Protocol for: Conroy T, Desseigne F, Ychou M, et al. FOLFIRINOX versus gemcitabine for metastatic pancreatic cancer. N Engl J Med 2011;364:1817-25.

PAC ACCORD
Actions concertées dans les Cancers COloRectaux et Digestifs

PROTOCOLE ACCORD 11/0402

N° EudraCT 2004-001985-42

ETUDE RANDOMISEE DE PHASE II / III COMPARANT L'ASSOCIATION FOLFIRINOX [OXALIPLATINE / IRINOTECAN / LV5FU] A LA GEMCITABINE EN PREMIERE LIGNE DE CHIMIOTHERAPIE DE PATIENTS ATTEINTS D'UN CANCER DU PANCREAS METASTATIQUE

Essai OXIPAN

Version n°9 contenant les amendements n°1, 2, 3, 5, 7, 8, 9 et 10 approuvée par le CPP EST-III
Le 07/05/2009

Investigateur Coordonnateur : Professeur Thierry CONROY
Département d'oncologie médicale
Centre Alexis Vautrin
6, avenue de Bourgogne
54511 Vandoeuvre-lès-Nancy cedex
Tel : 03.83.59.84.60
Fax : 03.83.59.83.59
e-mail : t.conroy@nancy.fnclcc.fr

Comité de rédaction : Thierry CONROY, Elisabeth LUPORSI (Nancy), Sophie GOURGOU-BOURGADE (Montpellier)
Christine MONTOTO-GRILLOT (Paris)
Pascal PIEDBOIS (Créteil), Michel DUCREUX (Villejuif), Olivier BOUCHE (Reims), Antoine ADENIS (Lille)
Jean Louis LEGOUX (Bordeaux), Yves BECOUARN (Bordeaux)
Marc YCHOU (Montpellier), Marc GIOVANNINI (Marseille)

Promoteur : **Fédération Nationale des Centres de Lutte Contre le Cancer (FNCLCC)**
101 rue de Tolbiac - 75654 PARIS CEDEX 13
Tél. : 01 44 23 04 04 - Fax : 01 44 23 55 69

APPROBATION ET SIGNATAIRES
DU PROTOCOLE ACCORD 11/0402 CONTENANT LES AMENDEMENTS N°1, 2, 3, 5, 7, 8, 9 et 10

N° EudraCT 2004-001985-42

ETUDE RANDOMISEE DE PHASE II / III COMPARANT L'ASSOCIATION FOLFIRINOX [OXALIPLATINE / IRINOTECAN / LV5FU] A LA GEMCITABINE EN PREMIERE LIGNE DE CHIMIOTHERAPIE DE PATIENTS ATTEINTS D'UN CANCER DU PANCREAS METASTATIQUE

Responsables	Nom	Coordonnées	Date (jj-mm-aa)	Signature
**Directeur scientifique du BECT (FNCLCC)**	**Dr Jocelyne BERILLE**	FNCLCC / BECT<br>101, rue de Tolbiac<br>75654 Paris cedex 13<br>Tél. : 01 44 23 04 19<br>Fax : 01 44 23 55 69<br>e-mail : j-berille@fnclcc.fr		
**Chef de Projet (FNCLCC)**	**Christine MONTOTO-GRILLOT**	FNCLCC / BECT<br>101, rue de Tolbiac<br>75654 Paris cedex 13<br>Tél. : 01 44 23 55 67<br>Fax : 01 44 23 55 69<br>e-mail : c-montoto-grillot@fnclcc.fr		
**Investigateur Coordonnateur**	**Pr. Thierry CONROY**	Centre Alexis Vautrin<br>6, avenue de Bourgogne<br>54511 Vandoeuvre-lès-Nancy cx<br>Tél : 03.83.59.84.60<br>Fax : 03.83.59.83.59<br>e-mail: t.conroy@nancy.fnclcc.fr		
**Biostatisticien**	**Sophie GOURGOU-BOURGADE**	Centre Val-D'Aurelle<br>Parc Euromédecine, Rue croix verte<br>34298 Montpellier cedex 5<br>tel :04.67.61.37.75<br>fax : 04.67.61.37.18<br>email :<br>Sophie.Gourgou@valdorel.fnclcc.fr		

Je reconnais avoir pris connaissance de l'ensemble du protocole ACCORD 11/0402 et je m'engage à conduire ce protocole conformément aux Bonnes Pratiques Cliniques, à la Loi Huriet et tel qu'il est décrit dans ce document.

J'assume les responsabilités qui m'incombent en tant qu'investigateur principal et dont notamment :

- le recueil du consentement éclairé, daté et signé par les patients avant toute procédure de sélection dans le protocole,
- la validation des cahiers d'observation complétés pour chacun des patients inclus dans l'étude,
- l'accès direct aux documents-source pour les vérifications effectuées par l'attaché de recherche clinique (ARC) mandaté par le promoteur,
- l'archivage des documents essentiels de l'étude pendant une durée de 15 ans.

<u>INVESTIGATEUR PRINCIPAL DE L'ETABLISSEMENT CONCERNE :</u>

Nom et adresse de l'établissement :

Nom de l'investigateur principal :

Date : Signature :

SYNOPSIS – PROTOCOLE ACCORD 11/0402

**PROMOTEUR**	Fédération Nationale des Centres de Lutte Contre le Cancer (FNCLCC).
**COORDONNATEUR**	Pr. Thierry CONROY<br>Département d'Oncologie Médicale - Centre Alexis Vautrin<br>6, avenue de Bourgogne<br>54511 Vandoeuvre-lès-Nancy cedex
**TITRE**	Etude randomisée de phase II / III comparant l'association Folfirinox [oxaliplatine / irinotecan / LV5FU] à la gemcitabine en première ligne de chimiothérapie de patients atteints d'un cancer du pancréas métastatique.
**PATHOLOGIE**	Adénocarcinomes pancréatiques métastatiques
**OBJECTIFS**	**Objectif principal de la phase II** :<br>▪ Comparer les taux de réponse objective entre les 2 bras de traitement.<br><br>**Objectif principal de la phase III** :<br>▪ Comparer la survie<br><br>**Objectif secondaire de la phase II** :<br>▪ Comparer la toxicité de ces traitements.<br><br>**Objectifs secondaires de la phase III** :<br>▪ Comparer la survie sans progression dans les 2 bras.<br>▪ Comparer la qualité de vie dans les 2 bras.<br>▪ Comparer le taux de réponse<br>▪ Comparer la toxicité de ces traitements
**METHODOLOGIE**	Etude randomisée de phase II / III comparant l'association Folfirinox [oxaliplatine / irinotecan / LV5FU] à la gemcitabine en première ligne de chimiothérapie de patients atteints d'un cancer du pancréas métastatique.
**CRITERES D'INCLUSION**	▪ Adénocarcinome du pancréas histologiquement ou cytologiquement prouvé,<br>▪ Maladie métastatique,<br>▪ Maladie mesurable en dehors d'un territoire irradié,<br>▪ Une lésion est mesurable si elle peut être mesurée avec précision dans au moins une dimension (le plus grand diamètre étant reporté) soit > à 20 mm avec un scanner conventionnel, soit > à 10 mm avec un scanner spiralé.<br>▪ Absence de chimiothérapie antérieure,<br>▪ Absence de radiothérapie abdominale antérieure (à l'exception d'une radiothérapie antalgique si elle n'a pas été effectuée sur les cibles mesurables),<br>▪ Age compris entre 18 et 75 ans inclus,<br>▪ Etat général : OMS 0-1,<br>▪ Fonction hématologique satisfaisante (PNN ≥ 1500/mm3, plaquettes ≥ 100 000/mm3),<br>▪ Fonction hépatique satisfaisante (bilirubine ≤ 1,5 fois la limite supérieure de la normale (un drainage biliaire est autorisé)),<br>▪ Fonction rénale satisfaisante: créatininémie < 120 µmol/L, soit 13.6 mg/L,<br>▪ Absence d'insuffisance cardiaque ou d'angine de poitrine non médicalement controlée ou d'infarctus dans les 12 mois précédents l'inclusion,<br>▪ Information du patient et signature du consentement éclairé.

CRITERES DE NON INCLUSION

- Autres types de tumeurs du pancréas, en particulier tumeur endocrine ou à cellules acineuses,
- Présence de métastases cérébrales ou méningées,
- Contre-indications spécifiques au traitement étudié,
- Antécédents de diarrhée chronique ou de maladie inflammatoire du côlon ou du rectum, ou d'occlusion ou de sub-occlusion non résolues sous traitement symptomatique,
- Infection évolutive active ou autre pathologie grave sous-jacente susceptible d'empêcher le patient de recevoir le traitement,
- Autre cancer concomitant ou antécédent de cancer en dehors d'un cancer in situ du col utérin ou d'un épithélioma baso ou spinocellulaire,
- Patient déjà inclus dans un autre essai thérapeutique avec une molécule expérimentale,
- Femme enceinte, susceptible de l'être ou en cours d'allaitement,
- Personnes privées de liberté ou sous tutelle,
- Impossibilité de se soumettre au suivi médical de l'essai pour des raisons géographiques, sociales ou psychiques.

DESCRIPTION SUCCINTE DES PRODUITS ET DEROULEMENT DU TRAITEMENT

Bras A : Folfirinox

- Oxaliplatine 85 mg/m^2 IV en perfusion de 2 heures,
- Irinotecan 180 mg/m^2 IV en perfusion de 90mn.
- Acide folinique 400 mg/m^2 IV en perfusion de 2 heures
- 5-FU bolus 400 mg/m^2 IV en 5 minutes.
- 5-FU continu 2,4 g/m^2 IV en perfusion continue sur 46 heures.

Reprise de cycle à J15.

Bras B : gemcitabine

- Gemcitabine 1000 mg/m^2 à J1, J8, J15, J22, J29, J36, J43.

Reprise à J57 et poursuite du traitement 3 semaines sur 4.

CRITERES D'EVALUATION

Pour la phase II
L'efficacité
Critère principal : taux de réponses objectives (RECIST).
Critère secondaire : Toxicités de grade 3-4

Pour la phase III

Critère principal : la survie
Critères secondaires : Qualité de vie (échelle EORTC QLQ-C30 version 3.0 tous les 14 jours).
Toxicités selon l'échelle NCI-CTC version 3.0
Survie sans progression, taux de réponse

NOMBRE DE PATIENTS NECESSAIRES

Pour la Phase II :

Les patients seront inclus en 3 paliers avec à chaque étape une évaluation du taux de réponse dans le bras A (Folfirinox)

Paliers	1er	2e	3e
**N**	20	30	40
**R**	2	4	6
**A**	8	11	-

N = nombre de patients prévus dans le bras A Folfirinox
R = nombre de patients ayant répondu au Folfirinox
A = nombre de patients ayant répondu au-delà duquel la phase II est interrompue. On passe alors directement en Phase III car on considère que le Folfirinox donne au moins 20% de réponses objectives.

◆ A la fin de la 1ère étape (20 malades inclus) :
- si l'on a observé 2 réponses objectives (RO) ou moins, on arrêtera

	l'essai en concluant que le Folfirinox n'est pas efficace.<br>- si l'on a observé 8 réponses objectives ou plus, on passera en phase III.<br>- si l'on a observé entre 3 et 7 réponses objectives, on continuera la phase II : inclusion de 10 malades supplémentaires.<br><br>◆ A la fin de la 2ème étape (30 malades en tout) :<br>- si l'on a observé 4 réponses objectives ou moins, on conclura que le Folfirinox n'est pas efficace.<br>- si l'on a observé 11 réponses objectives ou plus, on conclura que le Folfirinox est efficace et on passera à la phase III.<br>- si l'on a observé entre 5 et 10 réponses objectives, on continuera la phase II : inclusion de 10 malades supplémentaires.<br><br>◆ A la fin de la 3ème étape (40 malades en tout) :<br>- si l'on a observé 6 réponses objectives ou moins, on conclura que le Folfirinox n'est pas suffisamment efficace, on ne passe donc pas en phase III.<br>- si l'on a observé 12 réponses objectives ou plus, on conclura que le traitement est efficace et on passera à la phase III<br>- Entre 7 et 11, on ne peut pas conclure et on passe à la phase III pour avoir plus de patients.<br><br>Si nous considérons que 10 % des patients seront inévaluables, pour la réponse, il est donc nécessaire d'inclure **un total de 88 patients** répartis en **2 groupes** équivalents de **44 patients** sur une période de 2 ans<br><br>**<u>Pour la phase III :</u>**<br>Pour mettre en évidence une différence dans les médianes de survie de 3 mois (passage de 7 à 10 mois de médiane), soit un risque relatif de 0.70, il faudra inclure **360 patients** pour maintenir un risque global alpha de 5 %, en acceptant un risque beta de 20 % (puissance de l'essai = 80%).<br>Cette puissance sera obtenue lorsque **250** événements auront été observés (calcul effectué avec le logiciel East 5).<br><br>Les patients inclus dans la phase II seront pris en compte pour l'étude de phase III.
**NOMBRE DE CENTRES ESTIMES**	15 centres pour la phase II<br>50 centres pour la phase III
**DUREE DE L'ETUDE**	début des inclusions phase II : juin 2004<br>fin des inclusions phase II : juin 2006<br><br>début des inclusions phase III : juin 2006<br>fin des inclusions phase III : juin 2009<br><br>fin de l'étude : juin 2010
**ANALYSE STATISTIQUE**	L'analyse statistique sera effectuée sur la totalité des patients randomisés en intention de traiter. La survie globale des patients éligibles est calculée à partir de la date de randomisation, la survie sans progression (DFS) est calculée depuis la date de randomisation jusqu'à la date de rechute, les taux de survie sans progression seront estimés par la technique de Kaplan Meier et les comparaisons seront réalisées au moyen d'un test de Log-rank.<br>Un modèle de Cox sera utilisé pour l'analyse multifactorielle, prenant en compte les facteurs pronostiques majeurs de survie en maladie métastatique (état général et plus accessoirement, l'âge, la différenciation tumorale, l'albuminémie, les LDH et les phosphatases alcalines).

TABLE DES MATIERES

1 . INTRODUCTION ET RATIONNEL DE L'ESSAI 8

1.1 Faut-il traiter par chimiothérapie un cancer du pancréas exocrine métastatique ? 8
1.2 La gemcitabine est la chimiothérapie de référence 8
1.3 Résultats obtenus avec le 5-FU, l'irinotecan et l'oxaliplatine 8
1.4 Résultats de l'étude de phase II Folfirinox 9

2 . OBJECTIFS DE L'ESSAI 10

2.1 Objectif principal de la Phase II 10
2.2 Objectifs secondaires de la Phase II 10
2.3 Objectif principal de la Phase III 10
2.4 Objectifs secondaires de la Phase III 10

3 . METHODOLOGIE 10

4 . SELECTION DES PATIENTS 11

4.1 Critères d'inclusion 11
4.2 Critères de non inclusion 12

5 . RANDOMISATION / ENREGISTREMENT DES PATIENTS 12

6 . TRAITEMENTS 13

6.1 Description des traitements à l'essai 13
6.2 Déroulement du traitement 14
6.2.1 Bras A : Folfirinox : association oxaliplatine + irinotecan + acide folinique + 5 fluorouracile 14
6.2.2 Bras B : gemcitabine 14
6.2.3 Durée du traitement (pour les deux bras) 14
6.3 Adaptation des doses 14
6.3.1 Toxicité hématologique 15
6.3.1.1 Selon le bilan biologique à J15 15
6.3.1.2 Selon la toxicité hématologique de l'intercure (nadir) dans le bras Folfirinox 16
6.3.2 Toxicités digestives dans le bras Folfirinox 16
6.3.3 Mucites ou syndrome « main-pied » dans le bras Folfirinox 16
6.3.4 Toxicité cardiaque 16
6.3.5 Neuropathie périphérique 16
6.3.6 Adaptation de dose pour élévation de la bilirubine 17
6.3.7 Autres toxicités 17
6.4 Traitements symptomatiques des toxicités 17
6.4.1 Le syndrome cholinergique 17
6.4.2 La diarrhée tardive 17
6.4.3 La neutropénie 18
6.4.4 L'extravasation 18
6.4.5 Nausées et vomissements 18
6.4.6 L'alopécie 19
6.5 Traitements concomitants 19

7 . BILAN D'INCLUSION ET DE SUIVI DES PATIENTS 19

7.1 Bilan d'inclusion 19
7.2 Bilans de suivi 20
7.2.1 Bras A- FOLFIRINOX 20
7.2.2 Bras B- gemcitabine 20
7.3 Bilans à 8 semaines de traitement (autour de J 50) pour les 2 bras puis toutes les deux mois 20
7.4 Bilans de suivi après reprise du traitement (à partir de J57) 21
7.5 Bilans de suivi après les 6 mois de traitement 21
7.6 suivi à long terme 21

8 ARRET PREMATURE DU TRAITEMENT 22

9 . CRITERE D'EVALUATION 22

9.1 Critère principal de la phase II 22

9.2 Critères secondaires de la phase II 22
9.2.1 Toxicité 22
9.3 Critère principal de la phase III 22
9.3.1 Efficacité 22
9.4 Critères secondaires de la phase III 22
9.4.1 Survie sans progression 22
9.4.2 Taux de réponse 22
9.4.3 Toxicité 23
9.4.4 Qualité de vie 23

10 EVENEMENTS INDESIRABLES GRAVES 23

10.1 Définition Générale 23
10.2 Définition d'un événement indésirable grave attendu (EIG-A) 24
10.3 Définition d'un événement indésirable grave inattendu (EIG-I) 24
10.4 Critère d'intensité 24
10.5 Conduite à tenir en cas d'événement indésirable grave 24
10.6 Suivi des EIG 25

11 . DETERMINATION DU NOMBRE DE PATIENTS ET ANALYSE STATISTIQUE 26

11.1 Nombre de sujets nécessaires en phase II 26
11.2 Nombre de sujets nécessaires en phase III 26
11.3 Analyse statistique 26
11.4 Non respect du protocole 27
11.5 Analyses intermédiaires et surveillance de l'essai 27

12 . COUT ET SURCOUT DE LA RECHERCHE 27

13 . ASSURANCE QUALITE ET COMITE INDEPENDANT 28

14 . REGLES DE PUBLICATIONS PRODIGE 28

15 . ASPECTS ETHIQUES ET REGLEMENTAIRES 29

15.1 Comité Consultatif pour la Protection des Personnes dans la Recherche Biomédicale (CCPPRB) 30
15.2 Information et Consentement des participants 30
15.3 Responsabilités des investigateurs 30
15.4 Responsabilités du promoteur 31
15.5 Comité de Patients 31

16 . REFERENCES BIBLIOGRAPHIQUES 32

ANNEXES........ Erreur ! Signet non défini.

ANNEXE 1 : Modalités de préparation et d'administration des produits .... ***Erreur ! Signet non défini.***
ANNEXE 2 : Tableau récapitulatif des investigations .... ***Erreur ! Signet non défini.***
ANNEXE 3 : Evaluation de l'état général en fonction de l'échelle de l'ECOG ou de l'OMS ***Erreur ! Signet non défini.***
ANNEXE 4 : Classification des évaluations tumorales .... ***Erreur ! Signet non défini.***
ANNEXE 5 : Extrait des critères de toxicité (CTC–NCI) .... ***Erreur ! Signet non défini.***
ANNEXE 6 : Formulaire de notification d'un événement indésirable grave .... ***Erreur ! Signet non défini.***
ANNEXE 6 bis: Formulaire complémentaire d'information d'un EIG inattendu .... ***Erreur ! Signet non défini.***
ANNEXE 7 : Formulaire d'information destiné au patient(1) .... ***Erreur ! Signet non défini.***
ANNEXE 7 : Formulaire de consentement de participation du patient(1) .... ***Erreur ! Signet non défini.***
ANNEXE 8 : Conseils pour l'utilisation du questionnaire EORTC QLQ-30 pour mesurer la qualité de vie ***Erreur ! Signet non défini.***
ANNEXE 9 : EORTC QLQ-C30 (version 3.0) .... ***Erreur ! Signet non défini.***
ANNEXE 10 : recommandations pour l'utilisation du filgrastim : (NEUPOGEN®) .... ***Erreur ! Signet non défini.***
ANNEXE 11 : recommandations pour l'utilisation de la darbepoetin alfa (ARANESP®) ***Erreur ! Signet non défini.***
ANNEXE 12 : Liste des EIG attendus .... ***Erreur ! Signet non défini.***

1 . INTRODUCTION ET RATIONNEL DE L'ESSAI

1.1 Faut-il traiter par chimiothérapie un cancer du pancréas exocrine métastatique ?

Le cancer du pancréas métastatique reste une maladie incurable, associée à une médiane de survie de 2 à 4 mois en l'absence de chimiothérapie (1-2). Sept études ont comparé une chimiothérapie palliative à un traitement purement symptomatique (3-9). Quatre études datant des années 1980 ne montrent pas de différence de survie entre les deux groupes (3-6). Deux des 3 études les plus récentes ont montré une amélioration de la survie et de la qualité de vie en faveur du groupe recevant la chimiothérapie (7,8). Dans la première étude (7), les patients traités par chimiothérapie sont moins déprimés (échelle HADS). Dans la seconde étude (8), 38 % des patients traités par chimiothérapie ont une amélioration de leur qualité de vie (jugée par deux observateurs indépendants à partir des réponses au questionnaire EORTC QLQ-C30) pendant plus de quatre mois, contre 13 % des patients recevant un traitement symptomatique. Le groupe traité par chimiothérapie a un meilleur indice de Karnofsky, un meilleur état psychologique, et moins d'anorexie, de douleurs, de fatigue et de dyspnée.

Toutefois, les quelques études ouvertes qui ont étudié l'impact sur la régression des symptômes et la qualité de vie indiquent qu'au mieux 20 à 35 % des patients tirent bénéfice d'une chimiothérapie, le bénéfice étant habituellement de courte durée (10). En analyse multifactorielle, les facteurs pronostiques de survie en maladie métastatique sont surtout l'état général et plus accessoirement, l'âge, la différentiation tumorale, l'existence d'une anorexie, l'albuminémie, les LDH et les phosphatases alcalines (11-15-19-47-51) Une étude portant sur 3023 patients traités par gemcitabine montre également une médiane de survie particulièrement courte chez les patients dont l'indice de Karnofsky est inférieur à 70 (schématiquement équivalent à OMS 2), la médiane de survie étant de 2,4 mois et la survie sans progression de 1,7 mois (11). La décision de traiter les patients OMS 2 doit donc être prise au cas par cas.

1.2 La gemcitabine est la chimiothérapie de référence

A la suite d'une étude randomisée portant sur 126 patients (12), la gemcitabine 1000 mg/m^2 en administration hebdomadaire est considérée comme la monochimiothérapie de référence. Elle a été marginalement plus efficace que le 5-FU en bolus administré 5 jours toutes les quatre semaines. Cependant, avec la gemcitabine, seulement trois réponses partielles ont été observées parmi 56 patients présentant une maladie mesurable (5,4 %) alors qu'aucun des patients traités par 5-FU n'a répondu. La médiane de survie a été améliorée de 1,2 mois, passant de 4,4 mois pour les patients traités par 5-FU versus 5,6 mois pour les patients recevant la gemcitabine (p = 0,0025). Dans d'autres études randomisées, la gemcitabine en monothérapie est associée à une médiane de survie de 4.4 à 6.6 mois (13-17). Aucune étude randomisée n'a montré à ce jour une amélioration significative de la survie et/ou de la qualité de vie par rapport à la gemcitabine en monothérapie (17-40-41-42-44-46-47-48-49). La gemcitabine constitue actuellement le traitement de référence de la plupart des études randomisées (EORTC, FFCD, groupes coopérateurs américains).

1.3 Résultats obtenus avec le 5-FU, l'irinotecan et l'oxaliplatine

Le taux de réponse historique attribué au 5 Fluorouracile (15 % à 36 %) est très surestimé, comme le montrent 3 études randomisées récentes. Dans une étude portant sur 281 patients, le 5-FU en perfusion continue à la dose de 225 mg/m^2/j n'a amené un taux de réponse que de 1 % et une médiane de survie de 5,1 mois (18). A la dose de 300 mg/m^2/j, le taux de réponse observé chez 105 patients a été de 8,6 % et la médiane de survie de 5,1 mois (19). Dans l'étude de la FNCLCC, le taux de réponse du 5-FU en bolus était de 0 % versus 12 % pour une association de 5-FU en perfusion continue et cisplatine (20), sans avantage de survie significatif en faveur de l'association.

L'irinotecan est un médicament actif dans le cancer du pancréas, associé à des taux de réponse de 6 à 9 %, réponses d'une durée médiane de 5,7 mois avec une médiane de survie de 5,2 mois (21-22). Une efficacité en troisième ou quatrième ligne a également été décrite (45). L'oxaliplatine en monothérapie

n'est pas efficace, mais associé au 5 Fluorouracile et à l'acide folinique, un taux de réponse de 12 % a été décrit sur 28 patients. La médiane de survie était de 8,5 mois (23). Une synergie in vitro entre le SN-38, métabolite actif de l'Irinotecan, et l'oxaliplatine a été décrite (24).

Le SN-38, le métabolite principal de l'irinotecan, est plus cytotoxique sur des cultures d'adénocarcinome pancréatique que le cisplatine, la mitomycine C et le 5-FU (31). D'autres études pré-cliniques ont montré une synergie entre SN-38 et 5-FU lorsque le SN-38 précède l'exposition au 5-FU (32). D'autres études en laboratoire ont confirmé l'effet antitumoral de l'irinotecan sur des cultures de cellules tumorales pancréatiques et sur des xénogreffes de cancers du pancréas (34).

L'effet cytotoxique de l'oxaliplatine a été décrit sur trois lignées cellulaires pancréatiques différentes et sur des métastases hépatiques de cancers du pancréas (29). In vitro, la cytotoxicité est accrue lorsqu'on ajoute de l'oxaliplatine aux cultures avant ou en même temps qu'un dérivé de la camptothécine (35). Cette synergie semble liée à la stabilisation des adduits de l'oxaliplatine sur l'ADN lorsque les cellules tumorales sont exposées d'abord à l'oxaliplatine, puis à l'inhibiteur de topoisomérase. De ce fait, l'irinotecan pourrait réduire la résistance à l'oxaliplatine. En dehors de la toxicité hématologique, ces deux médicaments n'ont par ailleurs pas de toxicité croisée.

Dans l'étude de phase I de l'association Folfirinox (oxaliplatine, irinotecan, acide folinique, 5-FU en bolus, 5-FU en perfusion continue), il a été observé une réponse complète et une réponse partielle parmi cinq cas de cancers du pancréas (25).

1.4 Résultats de l'étude de phase II Folfirinox

Du fait des résultats encourageants observés en phase I, une étude de phase II de l'association Folfirinox a été mise en place dans 9 centres. Quarante-sept patients OMS 0-1, présentant une maladie localement avancée ou une maladie métastatique ont été inclus. Quarante-six patients ont été traités. Trente-quatre patients étaient en maladie métastatique et 12 présentaient une maladie localement avancée inopérable. Le nombre moyen de cures a été de 8 (1-24). La tolérance a été excellente. Sur 356 cycles, 14 % ont fait l'objet d'une réduction de dose. Quatre patients ont reçu du G-CSF. Deux épisodes de neutropénie fébrile sont survenues. Trente-cinq pour cent des patients ont présenté une neutropénie de grade 3 (15 % des cycles) et 17 % une neutropénie de grade 4 (7 % des cycles). Une diarrhée de grade 3 est survenue chez 11 patients et 3 % des cycles. Une diarrhée de grade 4 est survenue chez 2 % des patients et moins de 1 % des cycles. La neurotoxicité de l'oxaliplatine a été tolérable, avec 15 % de neuropathie de grade 3 (échelle de Lévi) et 3 % des cycles. Il n'y a pas eu de décès toxique.

En termes de taux de réponses, après relecture extérieure indépendante de tous les scanners, il a été observé deux réponses complètes, dix réponses partielles, vingt stabilisations tumorales, soit un taux de réponse de 26 % (intervalle de confiance : 13-39 %), et 61 % de contrôle tumoral. Le taux de réponse a été identique en maladie métastatique et en maladie localement avancée.

Une progression a été observée dans un délai moyen de 5,1 mois. La médiane de survie a été de 10,2 mois (9,5 mois en maladie métastatique et 15,5 mois dans les cancers localement avancés). Quarante trois pour cent des patients étaient en vie à un an. La durée moyenne de réponse objective a été de 9,3 mois (8-14 mois) et la durée moyenne de stabilité de 6,2 mois (26-27).

La qualité de vie était évaluée à l'inclusion et avant chaque cycle. La compliance au questionnaire a été de 66 %. Trente huit questionnaires sont disponibles à l'inclusion et 36 lors du dernier cycle. La comparaison entre les scores initiaux de qualité de vie et ceux du dernier cycle montre une amélioration de tous les domaines du QLQ-C30 (sauf domaine cognitif, difficultés financières et diarrhée). L'amélioration est surtout constatée chez les patients répondeurs ou stables. Une amélioration de plus de 10 points des scores est observée pour les domaines d'activités, psychologique, social, alors qu'une réduction des scores de symptômes est observée, surtout pour la douleur, l'insomnie, la perte d'appétit et la constipation. Chez les répondeurs, l'amélioration de qualité de vie globale est en moyenne de 25 points sur une échelle de 100 (soit une « grande » amélioration

selon les critères d'OSOBA (28)). Ces résultats encourageants justifient la réalisation d'une étude randomisée Folfirinox versus traitement de référence, la gemcitabine en monothérapie.

2 . OBJECTIFS DE L'ESSAI

2.1 Objectif principal de la Phase II

Evaluer l'efficacité de l'association oxaliplatine, irinotecan, 5 fluorouracile et acide folinique (Folfirinox – bras A) dans le traitement du cancer du pancréas métastatique par rapport au traitement de référence par gemcitabine (bras B), en comparant les taux de réponses objectives entre les 2 bras.

2.2 Objectifs secondaires de la Phase II

- Comparer la tolérance aux traitements, en particulier les toxicités de grade 3-4.

2.3 Objectif principal de la Phase III

L'objectif principal est de comparer les effets sur la survie globale de deux schémas thérapeutiques, l'association Folfirinox – bras A par rapport au traitement de référence par gemcitabine bras B.

2.4 Objectifs secondaires de la Phase III

- Comparer la survie sans progression dans les 2 bras
- Comparer la qualité de vie dans les 2 bras.
- Comparer le taux de réponse
- Comparer la toxicité aux traitements.

3 . METHODOLOGIE

Essai de phase II / III avec Bénéfice Individuel Direct (BID), randomisé, ouvert, multicentrique, comparant le traitement A (Folfirinox) au traitement B (gemcitabine).

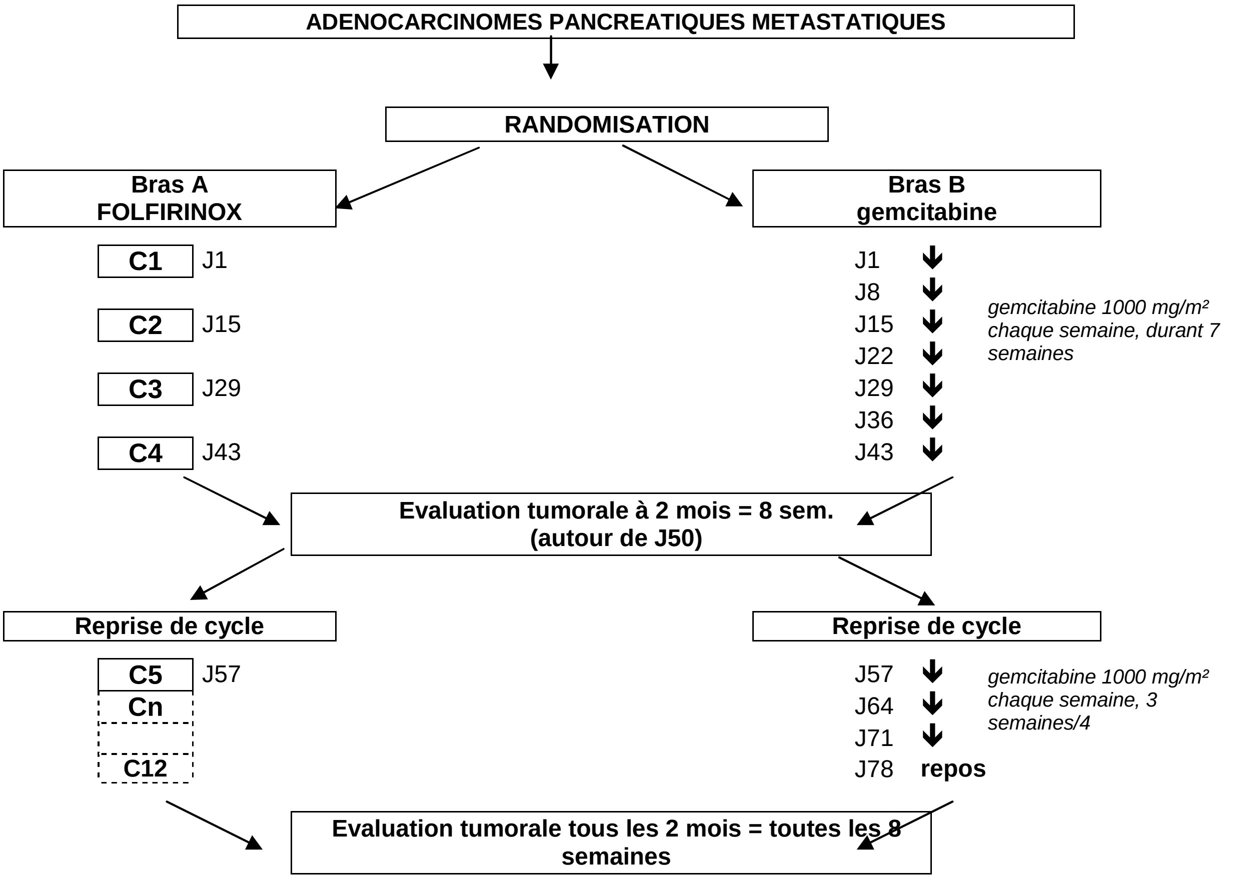

C1 = Cn = oxaliplatine 85 mg/m^2 J1+ irinotecan 180 mg/m^2 J1+ acide folinique 400 mg/m^2 J1, 5-FU bolus 400 mg/m² J1, 5-FU continu 2,4 g/m^2 sur 46 heures J1/ J2

Les patients seront traités pour un maximum conseillé de 6 mois.

4 . SELECTION DES PATIENTS

4.1 Critères d'inclusion

1. Adénocarcinome du pancréas histologiquement ou cytologiquement prouvé,
2. Maladie métastatique,
3. Maladie mesurable en dehors d'un territoire irradié,

   Une lésion est mesurable si elle peut être mesurée avec précision dans au moins une dimension (le plus grand diamètre étant reporté) soit > à 20 mm avec un scanner conventionnel, soit > à 10 mm avec un scanner spiralé.
4. Absence de chimiothérapie antérieure,
5. Absence de radiothérapie abdominale antérieure (à l'exception d'une radiothérapie antalgique si elle n'a pas été effectuée sur les cibles mesurables),
6. Age compris entre 18 et 75 ans inclus,
7. Etat général : OMS 0-1,
8. Fonction hématologique satisfaisante (PNN ≥ 1500/mm^3, plaquettes ≥ 100 000/mm^3),
9. Fonction hépatique satisfaisante (bilirubine ≤ 1,5 fois la limite supérieure de la normale (un drainage biliaire est autorisé)),

10. Fonction rénale satisfaisante: créatininémie < 120 µmol/L, soit 13.6 mg/L,
11. Absence d'insuffisance cardiaque ou d'angine de poitrine non médicalement controlée ou d'infarctus dans les 12 mois précédents l'inclusion,
12. Information du patient et signature du consentement éclairé.

4.2 Critères de non inclusion

1. Autres types de tumeurs du pancréas, en particulier tumeur endocrine ou à cellules acineuses,
2. Présence de métastases cérébrales ou méningées,
3. Contre-indications spécifiques au traitement étudié,
4. Antécédents de diarrhée chronique ou de maladie inflammatoire du côlon ou du rectum, ou d'occlusion ou de sub-occlusion non résolues sous traitement symptomatique,
5. Infection évolutive active ou autre pathologie grave sous-jacente susceptible d'empêcher le patient de recevoir le traitement,
6. Autre cancer concomitant ou antécédent de cancer en dehors d'un cancer in situ du col utérin ou d'un épithélioma baso ou spinocellulaire,
7. Patient déjà inclus dans un autre essai thérapeutique avec une molécule expérimentale,
8. Femme enceinte, susceptible de l'être ou en cours d'allaitement,
9. Personnes privées de liberté ou sous tutelle,
10. Impossibilité de se soumettre au suivi médical de l'essai pour des raisons géographiques, sociales ou psychiques.

5 . RANDOMISATION / ENREGISTREMENT DES PATIENTS

Après signature du formulaire de consentement et validation des résultats du bilan initial d'inclusion, les patients éligibles seront randomisés auprès du centre de randomisation de l'essai.
L'investigateur devra faxer la fiche de randomisation complétée et signée auprès du centre de gestion de la société EURAXI Pharma, Service de biométrie. En retour, le Centre de gestion des données faxera la confirmation de randomisation en précisant le bras de traitement et le numéro du patient.

Les coordonnées du centre de randomisation sont :

Gestion des Randomisations et Data-management

Sébastien LOUVEAU
EURAXI PHARMA
Service de Biométrie

du lundi au vendredi de 9h à 16h
Fax : 02.47.74.30.82 / Tél. : 02.47.74.30.47

Le tirage au sort est stratifié stratification classique sur :

- **le centre**
- **l'état général OMS (0 versus 1)**
- **localisation (tête versus autre)**

Le traitement doit débuter dans les 7 jours suivant la randomisation.

6 . TRAITEMENTS

6.1 Description des traitements à l'essai

A l'issue du tirage au sort, les sujets reçoivent soit le traitement A (FOLFIRINOX), soit le traitement B (gemcitabine) :

<u>Traitement A: Folfirinox</u>

- oxaliplatine (Eloxatine®)85 mg/m^2 J1 en 2h, puis
- irinotecan (Campto ®)180 mg/m^2 J1 en 90mn
- acide folinique 400 mg/m^2, J1 en 2h (pendant la perfusion d'irinotécan)
- 5-FU bolus 400 mg/m² J1 suivi de 5-FU continu 2,4 g/m^2 **au total** sur 46 heures, **soit 1,2 g/m^2 à J1 et 1,2 g/m^2 à J2.**.

Le traitement sera poursuivi jusqu'à progression et un maximum de 12 cycles est conseillé.

L'acide folinique peut être remplacé par du lévofolinate de calcium pour les centres qui le souhaitent. La dose devient alors 200mg/m^2.

<u>Traitement B: gemcitabine</u>

- gemcitabine 1000 mg/m^2 en 30 mn **par voie intra veineuse stricte**, à J1, J8, J15, J22, J29, J36, J43.

Reprise de la gemcitabine à J57, 3 semaines sur 4 (J57, J64, J71 suivi d'une semaine de pause).

Le traitement par gemcitabine sera poursuivi selon le même schéma (3 semaines sur 4) jusqu'à progression et un maximum de 6 mois est conseillé.

Les produits seront préparés selon les Bonnes Pratiques en Chimiothérapie et selon les modalités qui sont décrites en annexe 1.

L'irinotécan et l'oxaliplatine seront fournis par le Promoteur. Les autres produits seront pris dans le stock habituel de la pharmacie.

L'oxaliplatine et l'irinotécan seront fournis par le promoteur :

➔ ces produits seront étiquetés conformément à l'article R.5123 du Code de la Santé Publique et aux Recommandations de l'Annexe 1 des Bonnes Pratiques de Fabrication européennes.

➔ ces produits seront distribués aux différentes pharmacies des établissements de soins selon les Bonnes Pratiques de Distribution (BPD).

Une traçabilité de tous les produits utilisés dans le cadre de cette étude clinique, qui sont fournis soit par le promoteur ou soit par la pharmacie de l'établissement de soins, devra être assurée pendant toute la durée de l'étude.

6.2 Déroulement du traitement

6.2.1 Bras A : Folfirinox : association oxaliplatine + irinotecan + acide folinique + 5 fluorouracile

Le traitement débutera par l'administration d'oxaliplatine à la dose de 85 mg/m^2 IV en perfusion de 2 heures, puis sera suivi de l'administration simultanée de l'acide folinique à la dose de 400 mg/m^2 IV en perfusion de 2 heures et de l'irinotecan à la dose de 180 mg/m^2 IV en perfusion de 1H30, à débuter immédiatement après la fin de la perfusion d'oxaliplatine.
Le 5-FU sera administré immédiatement après la fin de la perfusion d'acide folinique et consistera en une dose de 400 mg/m^2 en bolus IV 5 minutes, puis en une perfusion continue sur 46 heures de 2,4 g/m².

Le cycle sera administré toutes les 2 semaines. Le traitement peut se faire en hôpital de jour.

Le traitement sera poursuivi jusqu'à rechute et un maximum de 12 cures est conseillé

6.2.2 Bras B : gemcitabine

L'administration de la gemcitabine se fera à la dose de 1000mg/m^2, en 30mn par intraveineuse stricte.

L'administration doit être répétée **une fois par semaine pendant 7 semaines suivie de 14 jours de repos**. Puis, à partir du cycle suivant, l'administration doit être répétée une fois par semaine pendant **3 semaines consécutives, suivie d'une semaine de repos**. Les doses pourront être réduites avant chaque administration de la chimiothérapie, en fonction de la tolérance individuelle des patients à la gemcitabine.

6.2.3 Durée du traitement (pour les deux bras)

Il est conseillé 6 mois de chimiothérapie. Au delà de cette période de traitement protocolaire, la prise en charge médicamenteuse ou non du patient, est laissé au libre choix de l'investigateur.

De même en cas de progression, la prise en charge thérapeutique du patient est laissé au choix de l'investigateur.

6.3 Adaptation des doses

En cas de réduction de doses nécessaire, les posologies réduites seront maintenues ultérieurement.
Toute toxicité de grade 4 récurrente à l'identique malgré une diminution de doses, amène le patient à arrêter le traitement.

**LES PATIENTS NE SORTENT JAMAIS DE L'ETUDE.<br>ILS SONT SUIVIS JUSQU'AU DECES.**

6.3.1 Toxicité hématologique

6.3.1.1 Selon le bilan biologique à J15

TRAITEMENT A : Folfirinox

NFS à J15	RETARD DE CYCLE	RÉDUCTION DE DOSE		
		**Irinotecan (CPT-11)**	**Oxaliplatine (L-OHP)**	**LV5FU**
PNN ≥ 1.5 x 10^9 /l<br>et plaq ≥ 75x10^9 /l	Pas de retard de cycle	Pas de réduction de dose		
PNN < 1.5 x 10^9 /l	Retarder le traitement jusqu'à PNN ≥ 1500 (jusqu'à J22 ou J29 si nécessaire).<br><br>En cas de non récupération à J29, **arrêt du traitement** *	1er épisode : réduction de dose à 150 mg/m^2<br><br>2ème épisode : maintien de la dose à 150 mg/m^2<br><br>3ème épisode : **arrêt du traitement**	1er épisode : pas de réduction de dose<br><br>2ème épisode : réduire la dose à 60 mg/m^2<br><br>3ème épisode : **arrêt du traitement**	1er épisode : supprimer le bolus de J1
Plaq < 75x10^9 /l	Retarder le traitement jusqu'à récupération (plaq ≥75x10^9 /l).<br><br>En cas de non récupération à J29, **arrêt du traitement**	1er épisode : pas de réduction de dose<br><br>2ème épisode : diminuer la dose à 150 mg/m^2<br><br>3ème épisode : **arrêt du traitement**	1er épisode : diminuer la dose à 60 mg/m²<br><br>2ème épisode : maintien de la dose réduite<br><br>3ème épisode : **arrêt du traitement**	1er épisode : diminuer la dose du bolus et de la perfusion continue de 25%

* *si pas de récupération après 2 retards de cure, arrêt du traitement sauf en cas de bénéfice clinique évident : le cas sera alors discuté avec le coordinateur de l'étude et le promoteur.*

TRAITEMENT B : GEMCITABINE

NFS avant chaque administration	RETARD DE CYCLE	RÉDUCTION DE DOSE
Granulocytes > 1 x 10^9 /l<br>**et** plaq > 100 x 10^9 /l	Pas de retard de cycle	Administrer 100 % de la dose totale
0,5 < Granulocytes ≤ 1 x 10^9 /l<br>**ou** 50 < plaq ≤ 100 x 10^9 /l	Pas de retard de cycle	Administrer 75 % de la dose totale (réduction de dose de 25%)
Granulocytes ≤ 0,5 x 10^9 /l<br>**ou** plaq ≤ 50 x10^9 /l	Retarder la cure jusqu'à récupération de 500 granulocytes et 50 000 plaq	Pas de réduction de dose

6.3.1.2 Selon la toxicité hématologique de l'intercure (nadir) dans le bras Folfirinox

ÉVENEMENTS	REDUCTION DE DOSE AU CYCLE SUIVANT
-Neutropénie fébrile isolée<br>-Neutropénie G4 de plus de 7 jours<br>-Infection avec neutropénie de grade 3-4 concomitante	1er épisode : réduire la dose de CPT-11 à 150 mg/m^2 et supprimer le bolus de 5-FU à J1<br>2ème épisode : malgré la réduction de dose de CPT-11 et la suppression du bolus de 5-FU, réduire la dose de L-OHP à 60 mg/m^2<br>3ème épisode : **arrêt du traitement**
Thrombopénie grade 3-4	1er épisode : réduire la dose L-OHP à 60 mg/m^2 et la dose de 5-FU continu de 25 %<br>2ème épisode : malgré la réduction de dose à 60 mg/m^2, réduire la dose de CPT-11 à 150 mg/m^2 et la dose de 5-FU continu de 25 % supplémentaires<br>3ème épisode : **arrêt du traitement**

6.3.2 Toxicités digestives dans le bras Folfirinox

ÉVENEMENTS	REDUCTION DE DOSE AU CYCLE SUIVANT
-Diarrhée grade **3-4** isolée ou<br>-Diarrhée + fièvre et/ou neutropénie grade 3-4	1er épisode : réduire la dose de CPT-11 à 150 mg/m^2 et supprimer le bolus de 5FU à J1<br>2ème épisode : malgré la diminution de dose du CPT-11 à 150 mg/m^2, réduire la dose de L-OHP à 60 mg/m^2 et réduire la dose de 5FU continu de 25 %<br>3ème épisode : **arrêt du traitement**
Diarrhée résistante (> 48 h) en dépit des hautes doses de lopéramide	Pas de réduction de dose du CPT-11 ni de L-OHP ni de 5-FU après récupération sauf si diarrhée grade 3-4, ou diarrhée + fièvre et/ou neutropénie grade 3-4

6.3.3 Mucites ou syndrome « main-pied » dans le bras Folfirinox

Ces toxicités sont le fait du 5-FU.
En cas de toxicité grade 3-4, une réduction de posologie de 25 % du 5-FU bolus et du 5-FU continu sera réalisée pour les cures suivantes.

6.3.4 Toxicité cardiaque

En cas d'angine de poitrine ou d'infarctus du myocarde, le traitement sera arrêté.

6.3.5 Neuropathie périphérique

Pour jugés la toxicité évaluables, les patients doivent avoir reçu au moins une cure ou une prise de traitement. L'échelle spécifique modifiée de Lévi et al. (37) n'étant pas assez précise, la toxicité neurologique sera évaluée selon l'échelle de toxicité NCI version 3.0 (annexe 5).

La toxicité neurologique de l'oxaliplatine demande une attention particulière et il faut signaler que sa cotation est complexe avec les critères CTC NCI version 3.0. Les différentes toxicités neurologiques peuvent être classées ainsi:

- Acro-dysesthésies au froid, sans douleurs ou dysesthésies (picotements, fourmillements) : sera coté en "Neuropathy, Sensory"
- Troubles moteurs objectifs découverts par l'examen clinique ou symptomatique : sera coté en "Neuropathy, Motor"
- Douleurs des mâchoires, crampes, neuropathies douloureuses : ne pas oublier de coter en plus en "Pain"

Si l'oxaliplatine est arrêté pour neurotoxicité, l'irinotécan et le 5-FU seront poursuivis.

6.3.6 Adaptation de dose pour élévation de la bilirubine

En cas d'élévation de la bilirubine il est nécessaire de prévoir une adaptation de dose de l'irinotécan.

ÉVENEMENTS	REDUCTION DE DOSE AU CYCLE SUIVANT
35 < Bilirubine ≤ 50 µmol/l	réduire la dose de CPT-11 à 75%
Bilirubine > 50 µmol/l	réduire la dose de CPT-11 à 50%

6.3.7 Autres toxicités

Toute autre toxicité ≥ grade 2, exceptées l'anémie et l'alopécie, pourra justifier une réduction de dose de chacun des protocoles si indiqué médicalement :

- Traitement A Folfirinox : réduction du CPT11 à 150 mg/m^2 et/ou L-OHP à 60 mg/m^2 et/ou le 5-FU de 25 % en fonction du type de toxicité
- Traitement B gemcitabine : réduction de dose de 25%

6.4 Traitements symptomatiques des toxicités

6.4.1 Le syndrome cholinergique

En cas de syndrome cholinergique aigu (hypersudation, hypersalivation, troubles visuels, larmoiements, myosis, crampes abdominales, diarrhée précoce) une injection par voie sous cutanée de 0,25mg d'atropine sera réalisée à titre curatif puis ensuite à titre préventif aux cures suivantes, sauf contre indication pour les patients traités par CPT-11.

6.4.2 La diarrhée tardive

Traitement prophylactique :

Aucun traitement prophylactique ne doit être donné, en particulier le lopéramide ne doit pas être administré de façon prophylactique. Cependant les patients doivent arrêter tout traitement laxatif et éviter les aliments et les boissons qui sont connus pour accélérer le transit intestinal.

Traitement curatif :

- dès la 1ère selle liquide ou molle le patient doit prendre immédiatement 2 capsules de lopéramide per os puis 1 capsule toutes les 2 h pendant au moins 12 h après la dernière selle liquide, sans dépasser un traitement total d'une durée de 48 h. La prise de boissons riches en électrolytes sera indiquée aussi au patient pendant tout l'épisode de diarrhée.

- En cas de persistance de la diarrhée plus de 48 h en dépit du traitement recommandé par lopéramide, une antibiothérapie à large spectre (fluoroquinolone) sera entreprise pour une durée systématique de 7 jours et après avis médical.

- En cas de diarrhée persistante et/ou sévère, le patient sera hospitalisé pour réhydratation parentérale et le lopéramide sera remplacé par un autre traitement antidiarrhéique laissé au choix du médecin investigateur.

- L'antibiothérapie orale par fluoroquinolone doit aussi être prescrite en cas de diarrhée grade 4 ou de diarrhée associée à une neutropénie de grade 3-4 ou à une fièvre.

- Les patients qui présentent des vomissements ou une fièvre ou un performance status > 2 concomitant à la diarrhée seront hospitalisés rapidement pour support parentéral. Le lopéramide et la fluoroquinolone doivent être prescrits au patient dès sa sortie d'hôpital afin qu'il ait l'un et l'autre à sa disposition dès l'apparition d'une diarrhée.

6.4.3 La neutropénie

En cas de neutropénie sévère c'est-à-dire grade 3-4, les patients sont à haut risque de neutropénie fébrile et d'infection notamment en cas de diarrhée concomitante. En cas d'apparition de ces symptômes, des adaptations posologiques sont prévues au cycle suivant (cf § 6.3.2).

L'administration de facteurs de croissance hématopoïétiques n'est pas recommandée au 1er cycle mais cependant ils peuvent être indiqués au cas par cas selon l'état clinique du patient. Un traitement par filgrastim (Neupogen®) est alors conseillé selon les recommandations jointes en annexe 10.

En cas d'anémie (par ex. taux d'hémoglobine ≤ 11 g/dl), un traitement par darbepoetin alfa (Aranesp®) sera mis en route selon les recommandations jointes en annexe 11.
Il est recommandé de ne pas administrer l'Aranesp® si le taux d'hémoglobine est > à 11 g/dL. Il est nécessaire de veiller à ce que ce taux ne dépasse pas 13 g/dL. En cas d'augmentation de taux d'hémoglobine de plus de 2 g/dL en quatre semaines, réduire la posologie de 25 à 50 %.

6.4.4 L'extravasation

Des réactions sévères liées à une extravasation de l'irinotecan ou d'oxaliplatine ont été rapportées (36). Les recommandations d'ordre général en cas d'extravasation sont les suivantes :
- arrêter immédiatement la perfusion,
- ne pas retirer l'aiguille ou le cathéter,
- aspirer par la même aiguille le maximum de produit infiltré,
- appliquer de la glace sur la zone infiltrée pendant 15 à 20 minutes toutes les 4 à 6 heures pendant 72 h,
- corticothérapie locale,
- vérifier régulièrement le site infiltré pendant les jours suivants, afin de vérifier si quelque traitement est nécessaire. Ne pas hésiter à prendre un avis chirurgical en cas de doute.

6.4.5 Nausées et vomissements

Avant l'administration de l'oxaliplatine une association de corticoïdes (30mn avant) et d'anti 5HT3 (sétron, 15mn avant) est recommandée.

Une prévention des nausées et vomissements retardés est également recommandée, utilisant du métoclopramide (ou un sétron) et éventuellement des corticoïdes.
ATTENTION : **En cas de diabète, l'utilisation des corticoïdes doit être faite avec la plus grande prudence.**

6.4.6 L'alopécie

Les casques réfrigérants du cuir chevelu sont autorisés, sauf en cas de métastases osseuses de la voûte du crane.

6.5 Traitements concomitants

Aucun traitement à visée antitumorale (chimiothérapie, hormonothérapie, modificateurs de la réponse biologique) ne sera utilisé.
Tous les traitements symptomatiques nécessaires au confort du patient (antiémétiques, antidiarrhéiques) sont autorisés et leur nature, leur posologie et leur durée d'administration, de même que tout autre traitement justifié par une indication médicale.
Les corticoïdes sont proscrits sauf en cas d'indication en urgence ou à visée antiémétique. Leur utilisation sera faite dans la plus grande prudence pour les patients diabétiques.
Une radiothérapie à visée antalgique est aussi autorisée (moelle osseuse irradiée < 20 %). Si la seule cible mesurable devait être irradiée, le patient arrêterait le traitement.

L'association de la warfarine (Coumadine®) avec un protocole FOLFOX est déconseillée (50). Il vaut mieux avoir recours à l'héparine et à ses dérivés. Si la warfarine ne peut être évitée, il faut contrôler plus fréquemment le taux de prothrombine et surveiller l'INR.

Le métronidazole et l'ornidazole augmentent la toxicité du 5-fluorouracile par diminution de sa clairance.

Risque de survenue de convulsion par interaction entre la phénytoïne et l'oxaliplatine. La phénytoïne est donc contre indiquée.

7 . BILAN D'INCLUSION ET DE SUIVI DES PATIENTS

La surveillance des patients depuis la date de tirage au sort et le calendrier des évaluations des patients sont décrits en annexe 2.

7.1 Bilan d'inclusion

Les patients éligibles pour l'essai et ayant signé leur consentement de participation devront subir un bilan initial **dans les 8 jours** précédant le début du traitement, hormis les examens du bilan paraclinique qui pourront être réalisés **dans les 3 semaines** précédant la randomisation.

- **Examen clinique**
  Examen clinique avec détermination du poids, de la taille et de la surface corporelle,
  Indice de performance ECOG (annexe 3),
  Recueil des traitements concomitants.

- **Examen paraclinique**
  Scanner thoraco-abdominopelvien,
  ECG

- **Examens biologiques**
  Hématologie et Coagulation : NFS, plaquettes, TP, TCK,
  Ionogramme sanguin, calcémie
  Bilan hépatique (bilirubine totale, libre et conjuguée, ALAT, ASAT, Phosphatases alcalines, LDH),
  Créatininémie, urée
  Glycémie
  Protidémie, albuminémie, glycosurie
  CA 19-9, ACE
  Test de grossesse si femme en âge de procréer et n'utilisant pas de procédé contraceptif

- **Questionnaire qualité de vie**
  Un questionnaire QLQ - C30 devra être rempli par le patient le jour de la signature du consentement à l'hôpital. Il peut donc être renseigné le jour même ou **dans les 15 jours** précédant la randomisation.

7.2 Bilans de suivi

Les patients seront revus toutes les semaines ou toutes les 2 semaines selon le bras de traitement

7.2.1 Bras A- FOLFIRINOX

A J 8 du premier cycle

- NFS plaquettes, Créatininémie, Kalièmie

Avant chaque cycle : tous les 14 jours
Les patients traités par FOLFIRINOX seront revus avant chaque reprise de cycle pour un bilan clinique afin d'évaluer les toxicités et de déterminer la reprise du traitement.

- évaluation de la tolérance pendant l'intercure
- vérification de la récupération d'éventuelles toxicités au niveau initial ou grade ≤ 1 (sauf alopécie)
- examen clinique complet, avec poids et OMS
- NFS, plaquettes
- Questionnaire de qualité de vie
- Protidémie, ionogramme, créatininémie, urée, glycémie
- Bilirubine totale, libre et conjuguée (au moins tous les mois)
- Questionnaire de qualité de vie EORTC QLQ-C30

Cependant en fonction de la tolérance, des visites de consultation supplémentaires seront possibles.

Tous les examens révélant une toxicité liée au traitement doivent être répétés périodiquement jusqu'à réversion de la toxicité ou jusqu'à ce quelle soit présumée irréversible

7.2.2 Bras B- gemcitabine

Avant chaque administration : tous les 7 jours jusqu'à J43
Les patients traités par gemcitabine seront revus avant chaque administration pour un bilan clinique afin d'évaluer les toxicités et décider de la poursuite du traitement.

- évaluation de la tolérance
- vérification de la récupération d'éventuelles toxicités au niveau initial ou grade ≤ 1 (sauf alopécie)
- NFS, plaquettes

Tous les 15 jours

- vérification de la récupération d'éventuelles toxicités au niveau initial ou grade ≤ 1 (sauf alopécie)
- examen clinique complet, avec poids et OMS
- NFS, plaquettes,
- Bilirubine totale, libre et conjuguée, phosphatases alcalines, ASAT, ALAT
- Urée, Créatininémie
- Ionogramme sanguin, Calcémie, protéines totales
- Questionnaire de qualité de vie EORTC QLQ-C30

7.3 Bilans à 8 semaines de traitement (autour de J 50) pour les 2 bras puis toutes les deux mois

- examen clinique complet, avec appréciation du poids , de l'état général OMS et des différentes toxicités
- **Bilan biologique avec :**
  NFS, plaquettes,

Bilan hépatique complet (bilirubine totale, libre et conjuguée, phosphatases alcalines, ASAT, ALAT)
Ionogramme sanguin, glycémie
Créatininémie, urée
Protidémie

- **Marqueurs tumoraux :**
  ACE, CA 19-9 (uniquement le marqueur le plus significatif lors du bilan initial : choisir le marqueur qui a la valeur initiale la plus importante par rapport à la normale)

- **Evaluation comparative des lésions tumorales :**
  Scanner thoracoabdomino-pelvien (selon les cibles initiales)

- Questionnaire de qualité de vie EORTC QLQ-C30

7.4 Bilans de suivi après reprise du traitement (à partir de J57)

Les patients seront suivis après reprise du traitement selon les mêmes modalités que décrites dans le § 7.2, avec remise d'un questionnaire de qualité de vie tous les 15 jours, un examen scanographique, un bilan hépatique, une créatininémie, et un dosage de ACE ou Ca 19-9 tous les 2 mois.

7.5 Bilans de suivi après les 6 mois de traitement

Les patients sans progression ayant eu les 6 mois de traitements seront suivis tous les 2 mois. Seront évalués la qualité de vie avec remise du questionnaire EORTC QLQ-C30 et la durée de réponse ou de stabilisation, grâce à une imagerie.

7.6 suivi à long terme

Les patients ayant progressé seront suivis tous les 6 mois jusqu'au décès. Seront évalués : les effets toxiques à long terme et la survie. Si un autre traitement est établi, il devra être rapporté.

8 ARRET PREMATURE DU TRAITEMENT

Les patients peuvent arrêter prématurément le traitement pour les raisons suivantes :

- toxicité,
- progression de la maladie,
- retrait de consentement,
- perdu de vue,
- violation majeure de protocole.

Dans la mesure du possible, les patients ayant arrêté prématurément leur traitement seront suivis selon les mêmes modalités que les autres patients.

9 . CRITERE D'EVALUATION

9.1 Critère principal de la phase II

Sera prise en compte la meilleure réponse enregistrée entre le début du traitement et la progression de la maladie ou la dernière évaluation (meilleure réponse globale optimale).
La réponse est définie selon les critères RECIST (annexe 4).

Toutes les réponses objectives doivent être confirmées 4 semaines après leur observation par un nouvel examen.

9.2 Critères secondaires de la phase II

9.2.1 Toxicité

Pour être jugés évaluables pour la toxicité, les patients doivent avoir reçu au moins perfusion de chimiothérapie

- La toxicité est évaluée selon l'échelle de toxicité NCI version 3.0 (annexe 5)

9.3 Critère principal de la phase III

9.3.1 Efficacité

Le critère principal de cette phase sera la durée de survie globale.

9.4 Critères secondaires de la phase III

9.4.1 Survie sans progression

La survie sans progression est calculée de la date de randomisation à la date de première mise en évidence d'une progression documentée, la date du décès, ou la date des dernières nouvelles.

9.4.2 Taux de réponse

Sera prise en compte la meilleure réponse enregistrée entre le début du traitement et la progression de la maladie ou la dernière évaluation (meilleure réponse globale optimale).
La réponse est définie selon les critères RECIST (annexe 4).

Toutes les réponses objectives doivent être confirmées 4 semaines après leur observation par un nouvel examen.

Un comité indépendant de 2 radiologues experts relira toutes les évaluations tumorales radiologiques pour confirmer les réponses enregistrées.

9.4.3 Toxicité

Pour être jugés évaluables pour la toxicité, les patients doivent avoir reçu au moins une cure ou une injection de traitement.

- La toxicité est évaluée selon l'échelle de toxicité NCI version 3.0 (annexe 5).

9.4.4 Qualité de vie

La qualité de vie sera mesurée grâce au questionnaire EORTC QLQ-C30 (39) (annexe 9), et analysée conformément aux spécifications du Groupe Qualité de Vie de l'EORTC (43)

L'analyse principale portera sur les domaines de qualité de vie qui sont habituellement les plus altérés. Les échelles les plus détériorées sont « santé globale / Qualité de vie » (questions 29 et 30), fatigue, douleur, forme physique, état psychologique, activités quotidiennes (38). Les changements sous traitement de la « santé globale/ Qualité de vie » seront considérés comme l'objectif principal et les autres critères comme des objectifs secondaires. Les autres domaines du QLQ-C30 seront analysés uniquement à visée exploratoire. (43)

Le questionnaire EORTC-PAN 24, spécifique de cancer du pancréas ne sera pas utilisé car ce questionnaire n'est pas encore validé et il n'a pas fait l'objet d'une adaptation transculturelle, ce qui le rend inadapté à des patients de langue française.

10 EVENEMENTS INDESIRABLES GRAVES

10.1 Définition Générale

N'est pas considéré comme un événement indésirable grave (EIG) :

- Une hospitalisation < à 24 heures,
- Une hospitalisation programmée préalablement au début de l'essai et/ou prévue par le protocole (biopsie, chimiothérapie..).

Est considéré comme un événement indésirable grave (EIG) tout évènement :

- Entraînant le décès,
- Mettant en jeu le pronostic vital,
- Entraînant une hospitalisation ou une prolongation d'hospitalisation,
- Provoquant une invalidité permanente ou une incapacité temporaire grave,
- Provoquant une anomalie congénitale, une malformation fœtale ou un avortement,
- Médicalement significatif.

  Les termes *invalidité et incapacité* correspondent à tout handicap physique ou psychique temporaire ou permanent, cliniquement significatif et retentissant sur l'activité physique et/ou la qualité de vie du patient.

  Est considéré comme *médicalement significatif* tout événement clinique ou résultat de laboratoire considéré comme grave par l'investigateur et ne correspondant pas aux critères de gravité définis ci-dessus. Ils peuvent faire courir un risque au patient et nécessitent une intervention médicale pour prévenir une issue correspondant à l'un des critères de gravité mentionné précédemment *(exemples : surdosages, seconds cancers, grossesses et faits nouveaux peuvent être considérés comme médicalement significatifs*).

10.2 Définition d'un événement indésirable grave attendu (EIG-A)

Un EIG-A est un événement déjà mentionné dans la version la plus récente de la brochure investigateur ou dans le résumé des caractéristiques du produit (RCP) pour les médicaments *ayant déjà une autorisation de mise sur le marché (AMM)*. Cette définition s'applique également au médicament de l'essai lorsqu'il est administré *pour une même population hors indication de l'AMM*.

10.3 Définition d'un événement indésirable grave inattendu (EIG-I)

Un EIG-I est un événement non mentionné ou différent par sa nature, son intensité, son évolution par rapport à la brochure investigateur ou au résumé des caractéristiques du produit (RCP) pour les médicaments ayant une autorisation de mise sur le marché (AMM).

10.4 Critère d'intensité

Le critère d'intensité ne doit pas être confondu avec le critère de gravité qui sert de guide pour définir les obligations de déclaration.

L'intensité des événements sera estimée selon la classification NCI-CTC version 3.0 (toxicité de grade 1 à 5). L'intensité des évènements indésirables non listés dans cette classification sera appréciée selon les qualificatifs suivants :

Légère (grade 1) : n'affecte pas l'activité quotidienne habituelle du patient
Modérée (grade 2) : perturbe l'activité quotidienne habituelle du patient
Sévère (grade 3) : empêche l'activité quotidienne habituelle du patient
Très Sévère (grade 4) : impose des mesures de réanimation/ menace le pronostic vital
Décès (grade 5)

10.5 Conduite à tenir en cas d'événement indésirable grave

L'investigateur informe la pharmacovigilance du BECT (PV-BECT) de tous les **Evènements Indésirables Graves Attendus (EIG-A) et inattendus (EIG-I),** qu'ils soient imputables ou non à la recherche, qui se produisent durant l'étude ou dans les 30 jours suivant la dernière administration du traitement.

Tous les Événements Indésirables Graves retardés (survenant après cette période de 30 jours) considérés comme raisonnablement liés au(x) traitement(s) protocolaire ou à la recherche doivent être déclarés sans limitation de délai.

La déclaration se fait par envoi par fax à la PV du BECT du formulaire de "***notification d'un événement indésirable grave***" (cf annexe n° 6) documenté le plus précisément poss ible, daté et signé, dans les **48 heures** ouvrées suivant leur constatation au :

Bureau d'Etudes Cliniques et Thérapeutiques
Pharmacovigilance
Tél. : 01 44 23 04 16 – Fax : 01 44 23 55 70
Courriel : pv-bect@fnclcc.fr

L'investigateur notera pour chaque évènement :

- Sa description aussi clairement que possible selon la terminologie médicale,
- Si l'évènement est attendu ou inattendu,
- L'intensité,
- La date de début et de fin de l'événement,
- Les mesures entreprises et la nécessité ou non d'un traitement correcteur,
- Si le traitement de l'essai a été interrompu,

- Son évolution. En cas d'évènement non fatal, l'évolution devra être suivie jusqu'à la guérison ou le retour à l'état antérieur ou à la stabilisation d'éventuelles séquelles,
- La relation de causalité entre cet évènement et le traitement à l'essai ou une contrainte liée à la recherche (période sans traitement, examens complémentaires demandés dans le cadre de la recherche etc ..),
- La relation de causalité avec le(s) médicaments de l'essai, la pathologie traitée, une autre pathologie ou un autre traitement.

L'investigateur doit également joindre au rapport d'évènement indésirable grave, à chaque fois que possible :

- Une copie du compte-rendu d'hospitalisation ou de prolongation d'hospitalisation,
- Une copie du rapport d'autopsie ,
- Une copie de tous les résultats d'examens complémentaires réalisés, y compris les résultats négatifs pertinents en y joignant les valeurs normales du laboratoire,
- Tout autre document qu'il juge utile et pertinent.

Tous ces documents doivent être anonymisés.

Des compléments d'informations pourront être demandés (par fax, par téléphone ou lors d'une visite) par le moniteur.

En cas d'**EIG inattendu et raisonnablement relié à un des traitements de l'étude, un complément d'information sera demandé par la pharmacovigilance à l'investigateur.** Celui-ci devra adresser au promoteur dans les **48 heures** le formulaire ***"complément d'information d'un EIG inattendu"*** (cf annexe n°6 bis) dûment renseigné.

Une liste des EIG attendus, établie à partir des RCP des produits est annexée à ce protocole (cf annexe 12).

D'autre part, une liste synthétique des EIG attendus, établie à partir du RCP et reclassée selon l'échelle de toxicité NCI-CTC V3, sera jointe au cahier d'observation afin de faciliter le remplissage du formulaire EIG par l'investigateur.

Néanmoins, tout événement attendu mais différent par son intensité, son évolution ou sa fréquence sera considéré comme inattendu par la pharmacovigilance.

10.6 Suivi des EIG

L'investigateur est responsable du suivi médical approprié des patients jusqu'à la résolution ou la stabilisation de l'effet ou jusqu'au décès du patient. **Cela peut impliquer parfois que ce suivi se prolonge après la sortie du patient de l'essai.**

Il transmet les informations complémentaires à la PV du BECT à l'aide d'un formulaire de déclaration des EIG (en cochant la case Suivi n° X pour préciser qu'il s'agit d'un follow-up et non d'un rapport initial) dans les 48 heures suivant leur obtention. Il transmet également le dernier suivi à la résolution ou à la stabilisation de l'EIG.

Il conserve les documents concernant l'effet indésirable présumé afin de permettre, en cas de nécessité de compléter les informations précédemment transmises.

Il répond aux demandes d'informations complémentaires de la PV du BECT afin de documenter l'observation initiale.

11 . DETERMINATION DU NOMBRE DE PATIENTS ET ANALYSE STATISTIQUE

11.1 Nombre de sujets nécessaires en phase II

On décide de conclure à l'efficacité du Folfirinox si le taux de réponses objectives est supérieur ou égal à 24 % et de conclure à son inefficacité si ce taux est inférieur ou égal à 10 %. Le calcul du nombre de sujets est effectué en utilisant la procédure de Fleming multi-étapes (27). On décide d'inclure au plus 44 sujets en 3 étapes par bras, soit 88 patients en tout (puissance de 92% formulation bilatérale, au seuil 5 %).

Dans le bras expérimental (Folfirinox):

◆ A la fin de la 1ère étape (20 malades inclus) :
- si l'on a observé 2 réponses objectives (RO) ou moins, on arrêtera l'essai en concluant que le Folfirinox n'est pas efficace.
- si l'on a observé 8 réponses objectives ou plus, passera à la phase III.
- si l'on a observé entre 3 et 7 réponses objectives, on continuera la phase II : inclusion de 10 malades supplémentaires.

◆ A la fin de la 2ème étape (30 malades en tout) :
- si l'on a observé 4 réponses objectives ou moins, on conclura que le Folfirinox n'est pas efficace.
- si l'on a observé 11 réponses objectives ou plus, on conclura que le Folfirinox est efficace et on passera en phase III.
- si l'on a observé entre 5 et 10 réponses objectives, on continuera l'essai : inclusion de 10 malades supplémentaires.

◆ A la fin de la 3ème étape (40 malades en tout) :
- si l'on a observé 6 réponses objectives ou moins, on conclura que le Folfirinox n'est pas suffisamment efficace et on ne passe pas en phase III.
- s'il y a 12 réponses, on poursuivra l'étude en phase III.
- si après 40 malades il n'y a pas de conclusion, on passera alors en phase III en incluant le nombre de patients supplémentaires.

Ce plan a une puissance égale à 0.92 (on a 92 chances sur 100 de conclure à l'efficacité si le taux de réponses objectives est égal à 60%). Le risque alpha est égal à 0.05 (on a 5 chances sur 100 de conclure à l'efficacité si le taux de non progression est égal à 35%).

11.2 Nombre de sujets nécessaires en phase III

Pour mettre en évidence une différence dans les médianes de survie de 3 mois (passage de 7 à 10 mois de médiane), soit un risque relatif de 0.70, il faudra **inclure 360 patients** pour maintenir un risque global alpha de 5 %, en acceptant un risque beta de 20 % (puissance de l'essai = 80%).
Cette puissance sera obtenue lorsque **250 événements** auront été observés (calcul effectué avec le logiciel East 5).

11.3 Analyse statistique

L'analyse statistique finale devra être réalisée lorsque **250 événements** auront été observés

Les données seront présentées sous forme de :

1. pourcentages (variables qualitatives),
2. moyenne et écart type ou médiane et extrêmes (variables quantitatives),

3. courbes de survie estimées par la méthode de Kaplan Meier (données de survie) (27). La date d'origine sera la date du tirage au sort.

Les résultats concernant le critère principal et les critères secondaires seront présentés avec un intervalle de confiance à 95% (Rothman pour les données de survie).

On comparera :

1. les variables qualitatives par un test du χ^2 ou par un test de Fisher,
2. les variables quantitatives par un test t de Student ou par un test non paramétrique (Wilcoxon),
3. les données de survie par un test du log-rank.

Ces comparaisons seront ajustées sur les facteurs de stratification.
Tous les tests seront bilatéraux au seuil 5 %.

Les analyses statistiques seront réalisées avec le logiciel Stata v10.

11.4 Non respect du protocole

On n'acceptera aucune exclusion. Les sujets inclus à tort et les sujets qui ne respecteront pas le protocole seront pris en compte dans l'analyse selon leur groupe de randomisation (analyse en intention de traiter).
Afin de limiter le nombre de perdus de vue, l'investigateur de chaque centre se chargera de reconvoquer les malades ne s'étant pas présentés aux consultations de surveillance.

11.5 Analyses intermédiaires et surveillance de l'essai

Une analyse intermédiaire d'efficacité sera réalisée lorsque 2/3 des événements (décès) auront été observés soit **167 événements**.
Afin de maintenir un risque global de 5%, cette analyse intermédiaire ne sera considérée comme significative que si la p-value est inférieure ou égale à 0,001 et **p≤0,049** à l'analyse finale.

Le Comité de Surveillance (IDMC) pourra proposer l'arrêt prématuré de l'essai s'il le juge nécessaire et si l'ensemble des données disponibles provenant de l'essai ou d'autres sources est suffisamment convaincant pour influencer les pratiques thérapeutiques de la majorité des médecins.

12 . COUT ET SURCOUT DE LA RECHERCHE

Les éventuels frais supplémentaires visés à l'article R.1121-1 du Code de la Santé Publique font l'objet d'une convention négociée entre la FNCLCC et le représentant de l'établissement en tenant compte des moyens financiers dont dispose la FNCLCC dans le cadre de son activité de promotion publique.

Cependant, la FNCLCC assure l'organisation de l'essai et la prise en charge de la fourniture du matériel suivant : protocole, cahier d'observation, dossier investigateur ; ainsi que la fourniture des produits à l'étude irinotécan et oxaliplatine.

Le matériel, les traitements, ou une prestation fournis par d'autres partenaires, doivent être précisés dans la convention de l'essai.

13 . ASSURANCE QUALITE ET COMITE INDEPENDANT

Le promoteur est responsable de la mise en place d'un système d'assurance qualité décrit dans les procédures fédérales, afin que l'essai soit réalisé conformément au protocole et aux BPC.
Une revue centralisée du critère d'efficacité sera réalisée par un comité d'experts indépendants.

14 . REGLES DE PUBLICATIONS PRODIGE

La publication des essais PRODIGE dans un délai rapide par une revue de qualité est un objectif essentiel pour le progrès de la thérapeutique. Cette publication se fait sous la responsabilité du Comité de Coordination du PRODIGE (CCP), qui décide :

- du moment de la publication des résultats préliminaires et des résultats définitifs d'une étude. .
- de la composition d'un Comité de Rédaction (5 membres au maximum).

Toutes les informations résultant d'essais sont considérées comme confidentielles, au moins jusqu'à ce que l'analyse appropriée et le contrôle par le promoteur, l'investigateur coordonnateur et le statisticien de l'essai soient achevés.

Le Comité de Coordination (CCP) peut déléguer ces fonctions au coordonnateur de l'essai.

En tout état de cause, le CCP valide les choix faits et veille à ce que les délais soient tenus. L'absence de réponse du CCP dans un délai d'un mois après soumission du Comité de Rédaction vaut acceptation.

1) Le Comité de Rédaction comprend :

- Le coordinateur (ou les coordinateurs s'ils sont deux) qui a écrit le premier projet. Il sera sauf exception le rédacteur principal
- Le(s) statisticien(s) ayant effectué l'analyse des données
- Les contributeurs les plus importants
- Eventuellement un spécialiste ayant fourni une contribution essentielle à l'analyse des données (biologiste, anatomopathologiste,…)

2) Le rédacteur principal s'engage à soumettre pour publication dans un délai déterminé par le CCP. Ce délai ne devrait pas dépasser une année après la clôture d'un essai. S'il n'est pas en mesure de le faire, le CCP désigne un nouveau rédacteur qui deviendra le premier auteur. Pour aider à l'écriture des articles issus des essais, il pourra être fait appel à un rédacteur médical et organisé des ateliers d'écriture pour le rédacteur principal, en collaboration avec le statisticien.
Avant toute publication la liste des inclusions par centre et la liste des investigateurs de chaque centre pour l'essai considéré sera mise à la disposition des investigateurs de l'essai.

3) Les auteurs de la publication sont dans l'ordre en fonction du travail fourni et du nombre de patients inclus :

- Le rédacteur principal
- Les membres du Comité de Rédaction de l'article (voir ci-dessus)
- Un nombre limité d'investigateurs (1 par centre) dans l'ordre de leur participation, et en règle 1 seul par centre mais pour certains centres le Comité Directeur peut décider 2 investigateurs. Cette règle pourra être pondérée pour permettre à certains centres petits et moyens ayant fait un gros effort d'inclusion de figurer parmi les auteurs. Le CCP validera cette pondération afin que personne ne soit lésé.
- Le nombre maximum d'auteurs autorisés par les revues sera utilisé.
- Quel que soit le nombre de malades inclus, il y aura au moins un auteur représentant un des deux partenaires (FFCD ou FNCLCC)
- Dans le cas d'une publication dérivée ou d'un travail annexe, les auteurs pourront être différents de ceux de l'article princeps et refléter la spécialité intéressée par l'article Ex :

dans les essais de RCT un article dédié à la radiothérapie peut être signé par des radiothérapeutes co-investigateurs des centres ayant inclus. Le dernier auteur de cette publication dérivée (éventuellement en « equally contributed ») est le premier signataire de l'article princeps.

- Le partenariat Prodige est cité dans le titre ou après les auteurs. En cas d'essai coopératif, la première association citée est celle qui a initié l'essai et les autres sont mentionnées à condition qu'elles aient inclus au moins 5 % des patients, dans l'ordre de leur participation.
- Sauf exception, pour les essais promus ou gérés par la FFCD un membre de l'unité Inserm U 866 sera dernier auteur, s'il n'est pas rédacteur principal, afin d'assurer la prise en compte de ce travail par l'Inserm. Dans ce cas l'avant-dernier auteur pourra être signalé comme ayant « equally contributed », si c'est applicable à la revue concernée.
- Le statisticien sera dans les auteurs, en règle générale au delà de la 3e place. Il pourra être le 1er ou 2e auteur d'une publication spécifique.

Tous les participants ne figurant pas dans les auteurs sont cités en fin d'article. Le gestionnaire de l'étude (Data manager) est également cité. Il peut être dans les auteurs si le CCP l'estime justifié.
Les partenaires sont remerciés.
Les auteurs et le promoteur reçoivent un manuscrit pour critique avant l'envoi à une revue. Ils s'engagent à répondre dans les 15 jours ouvrables pour que leur avis soit pris en compte (30 jours en période estivale).

4) <u>Communication orale à partir des résultats de l'essai :</u>

Un investigateur peut, avec l'accord du CCP et du Comité Directeur, présenter en son nom tout ou partie des résultats en communication orale. Les auteurs sont en règle générale les mêmes que pour l'article écrit, mais l'ordre des auteurs pour les articles et les communications peut varier, et varier aussi selon les congrès où la communication est faite. Dans certains cas (études multidisciplinaires, ou études pathologiques, biologiques, écho-endoscopiques parallèles à un essai thérapeutique par exemple) d'autres auteurs pourront être choisi en fonction de leur travail. Le partenariat PRODIGE et les autres associations, le cas échéant, doivent être cités.

15 . ASPECTS ETHIQUES ET REGLEMENTAIRES

L'essai clinique doit être conduit conformément aux principes éthiques de la déclaration d'Helsinki de 1964 révisée à Edimbourg en 2000, aux Bonnes Pratiques Cliniques de la Conférence Internationale d'Harmonisation (ICH–E6, 17/07/96), à la Directive Européenne (2001/20/CE) sur la conduite des essais cliniques, la loi Huriet modifiée (20/12/98) relative à la Protection des Personnes se prêtant à la Recherche Biomédicale ainsi qu'aux dispositions prévues par la Commission Nationale Informatique et Libertés (loi n°94-548 du 1/07/94 complétant la loi n°78-17 du 6/01/78).

15.1 Comité Consultatif pour la Protection des Personnes dans la Recherche Biomédicale (CCPPRB)

Le protocole d'essai clinique ainsi que les différents amendements ou toute information ou document jugé nécessaire par le promoteur sont soumis par l'investigateur coordonnateur de l'essai à l'avis d'un CCPPRB de la région où il exerce son activité (art. L. 1123-6). Les différents originaux des réponses écrites du CCPPRB doivent être transmis par le coordonnateur au promoteur.

15.2 Information et Consentement des participants

Préalablement à la réalisation d'une recherche biomédicale sur une personne, le consentement libre, éclairé et exprès de celle-ci doit être recueilli après qu'elle ait été informée, par l'investigateur ou son représentant, de l'objectif de la recherche, du déroulement et de la durée de l'étude, des bénéfices, risques potentiels et contraintes de l'essai ainsi que de la nature du produit étudié et de l'avis donné par le CCPPRB (art. L. 1122-1).

Le formulaire de consentement sera daté et signé personnellement par le patient et l'investigateur ou le médecin qui le représente (original archivé par l'investigateur, une copie sera remise au patient ou à son représentant légal).

Le formulaire d'information et de consentement éclairé (annexe 7) destiné au patient doit être associé sur un même document afin d'éviter tout risque de contestation sur le contenu de l'information donnée.

15.3 Responsabilités des investigateurs

L'investigateur principal de chaque établissement concerné s'engage à conduire l'essai clinique conformément au protocole qui a été approuvé par le CCPPRB. L'investigateur ne doit apporter aucune modification au protocole sans l'autorisation du promoteur et sans que le CCPPRB ait donné un avis favorable sur les modifications proposées.

Il est de la responsabilité de l'investigateur principal :

- de fournir au promoteur son curriculum vitae ainsi que ceux des co-investigateurs,
- d'identifier les membres de son équipe qui participent à l'essai et de définir leurs responsabilités,
- de démarrer le recrutement des patients après autorisation du promoteur,
- de faire le maximum pour inclure le nombre requis de patients dans les limites de la période de recrutement établie.

Il est de la responsabilité de chaque investigateur :

- de recueillir le consentement éclairé daté et signé personnellement par le patient avant toute procédure de sélection spécifique à l'essai,
- de compléter régulièrement les cahiers d'observation (CRF) pour chacun des patients inclus dans l'essai et de laisser à l'Assistant de Recherche Clinique (ARC) un accès direct aux documents-source afin que ce dernier puisse valider les données du CRF,
- de dater, corriger et de signer les corrections des CRF pour chacun des patients inclus dans l'étude,
- d'accepter les visites régulières de l'ARC et éventuellement celles des auditeurs mandatés par le promoteur ou des inspecteurs des autorités de tutelle.

Toute la documentation relative à l'étude (protocole, consentements, cahiers d'observation, dossier investigateur, etc…), ainsi que les documents originaux (résultats de laboratoire, radiologies, comptes-rendus de consultations, rapports d'examens cliniques pratiqués, etc.) doivent être détenus dans un lieu sûr et considérés comme du matériel confidentiel. L'archivage des données sera sous la responsabilité de l'investigateur et selon la législation en vigueur.

Ce dernier devra conserver les données ainsi qu'une liste d'identification des patientes pendant une durée minimale de 15 ans après la fin de l'étude.

15.4 Responsabilités du promoteur

Conformément à la loi Huriet, il est de la responsabilité du promoteur de :

- souscrire une assurance garantissant sa responsabilité civile en cas de conséquences dommageables de la recherche pour la personne qui s'y prête (art. L.1121-7),

- s'acquitter auprès de la Direction Régionale des Affaires Sanitaires et Sociales (DRASS) compétente du versement du droit fixe pour la consultation d'un comité de protection des personnes (CCPPRB) (Arrêté du 7/5/91 et du 13/12/01),

- transmettre à l'Agence Française de Sécurité Sanitaire des Produits de Santé (AFSSAPS), la déclaration d'intention initiale décrivant les données essentielles de la recherche accompagnée de l'avis du CCPPRB (art. L.1123-8),

- d'informer les Directeurs (art. L.1123-10, R.5124) et les Pharmaciens (art. L.5125-19, R.5124-1) des établissements de santé,

- communiquer aux investigateurs toutes les informations nécessaires pour la conduite de la recherche (art. R.5122),

- d'informer, dès qu'il en a connaissance, l'AFSSAPS de tout Evénement Indésirable Grave susceptible d'être dû à la recherche ainsi que tout arrêt prématuré de l'étude (art. L.1123-8).

Le promoteur doit également effectuer une déclaration des données médicales nominatives et informatisées, auprès du Comité Consultatif et de la Commission Nationale Informatique et Libertés (CNIL), conformément aux conditions d'application de la procédure simplifiée (Loi n°94-548 du 1/07/94 complétant la Loi n°78-17 du 6/01/78).

Le promoteur doit assurer l'archivage des documents essentiels sur la conduite de l'étude dans des conditions assurant leur sécurité, pendant la durée minimale prévue par les BPC, soit 15 ans après la fin de la recherche.

15.5 Comité de Patients

Le Comité de Patients s'engage dans le cadre des essais cliniques en cancérologie promus par la FNCLCC à relire le protocole et à proposer des améliorations portant notamment sur la qualité de la lettre d'information, la mise à disposition d'un plan de traitement et de surveillance, la suggestion de mesures visant à améliorer le confort des patients et selon la charte qui a été définie entre le Comité de Patients de la Ligue Nationale Contre le Cancer (LNCC) et le BECT de la FNCLCC.

16 . REFERENCES BIBLIOGRAPHIQUES

1. KELLY DM, BENJAMIN IS. Pancreatic carcinoma. *Ann Oncol* 1995; 6:19-28.

2. DUCREUX M, FRANCOIS E, N'GUYEN TD et al. Standards Options et recommandations pour la prise en charge des patients atteints de cancer du pancréas. In: Standards, Options et Recommandations- Recommandations pour la pratique clinique en cancérologie, vol.2. Fédération Nationale des Centres de Lutte Contre le Cancer (FNCLCC) ed. CD Rom, John Libbey Eurotext 1998.

3. MALLINSON CN, RAKE MO, COCKING JB et al. Chemotherapy in pancreatic cancer: results of a controlled, prospective, randomized multicentre trial. *Br Med J* 1980;281:1589-91.

4. ANDERSEN JR, FRIIS-MOLLER A, HANCKE S et al. A controlled trial of combination chemotherapy with 5-FU and BCNU in pancreatic cancer. *Scand J Gastroenterol* 1981;16:973-5.

5. FREY C, TWONEY P, KEEHN R et al. A randomized study of 5-FU and CCNU in pancreatic cancer: report of the Veterans Administration Surgical Adjuvant Cancer Chemotherapy Study Group. *Cancer* 1981;47:27-31.

6. ANDREN-SANDBERG A, HOLMBERG JT, IHSE I. Treatment of unresectable pancreatic carcinoma with 5-fluorouracil, vincristine and CCNU. *Scand J Gastroenterol* 1983;18:609-12.

7. PALMER KR, KERR M, KNOWLES G et al. Chemotherapy prolongs survival in inoperable pancreatic carcinoma. *Br J Surg* 1994;81:882-5.

8. GLIMELIUS B, HOFFMAN K, SJ.DEN P-O et al. Chemotherapy improves survival and quality of life in advanced pancreatic and biliary cancer. *Ann Oncol* 1996;7:593-600.

9. HUGUIER M, BARRIER A, VALINAS R et al. Randomized trial of 5-fluorouracil, leucovorin and cisplatin in advanced pancreatic cancer. *Hepatogastroenterology* 2001;48:875-8.

10. PERMERT J, HAFSTRÖM L, NYGREN P et al. A systematic overview of chemotherapy effects in pancreatic cancer. *Acta Oncol* 2001;40:361-70.

11. STORNIOLO AM, ENAS NH, BROWN CA et al. An investigational new drug treatment program for patients with gemcitabine : results for over 3000 patients with pancreatic carcinoma. *Cancer* 1999;85:1261-8.

12. BURRIS AH, MOORE MJ, ANDERSEN J et al. Improvements in survival and clinical benefit with gemcitabine as first-line therapy for patients with advanced pancreas cancer: a randomized trial. *J Clin Oncol* 1997;15:2403-13.

13. MOORE MJ, HAMM J, EISENBERG P et al. A comparison between gemcitabine (GEM) and the matrix metalloproteinase (MMP) inhibitor BAY12-9566 (9566) in patients (Pts) with advanced pancreatic cancer. *Proc Am Soc Clin Oncol* 2000;19:240a.

14. BRAMHALL SR, ROSEMURGY A, BROWN PD et al. Marimastat as first-line therapy for patients with unresectable pancreatic cancer: a randomized trial. *J Clin Oncol* 2001;19:3447-55.

15. BERLIN JD, CATALANO P, THOMAS JP et al. Phase III study of gemcitabine in combination with Fluorouracil versus Gemcitabine alone in patients with advanced pancreatic carcinoma : Eastern Cooperative Oncology Group trial E2297. *J Clin Oncol 2002 ; 20 : 3270-5.*

16. COLUCCI G, GIULANI F, GEBBIA V et al. Gemcitabine alone or with cisplatin for the treatment of patients with locally advanced and/or metastatic pancreatic carcinoma : a prospective, randomized phase III study of the Gruppo Oncologia del l'Italia Meridionale. *Cancer* 2002;94:902-10.

17. VAN CUTSEM E, KARASEK P, OETTLE H et al. Phase III trial comparing Gemcitabine + R115777 (Zarnestra) versus gemcitabine + placebo in advanced pancreatic cancer (PC). *Proc Am Soc Clin Oncol* 2002;21:130a.

18. ROY A, JACOBS R, BUKOWSKI R et al. Phase III trial of SMS 201-995 pa LAR (SMS PA LAR) and continuous infusion (CI) 5-FU in unresectable stage II, III, and IV pancreatic cancer. *Proc Am Soc Clin Oncol* 1998;17:257a.

19. MAISEY N, CHAU I, CUNNINGHAM D et al. Multicenter randomized phase III trial comparing protracted venous infusion (PVI) fluorouracil (5-FU) with PVI 5-FU plus mitomycin in inoperable pancreatic cancer. *J Clin Oncol 2002 ; 20 : 3130-6.*

20. DUCREUX M, ROUGIER P, PIGNON JP et al. A randomised trial comparing 5-FU plus cisplatin in advanced pancreatic carcinoma. *Ann Oncol* 2002;13:1185-91.

21. WAGENER TDJ, VERDONK HER, DIRIX LY et al. Phase II trial of CPT-11 in patients with advanced pancreatic cancer, an EORTC early clinical trials group study. *Ann Oncol* 1995;6:129-32.

22. SAKATA Y, SHIMADA Y, YOSHINO M et al. A late phase II study of CPT-11, Irinotecan hydrochloride, in patients with advanced pancreatic cancer. *Jpn J Cancer Chemother* 1994;21:1039-46.

23. DUCREUX M, MITRY E, OULD-KACI M, BOIGE V, SEITZ JF, BUGAT R, BREAU JL, BOUCHE O, ETIENNE PL, TIGAUD JM, MORVAN F, CVITKOVIC E, ROUGIER P. Randomized phase II study evaluating oxaliplatin alone, oxaliplatin combined with infusional 5-FU, and infusional 5-FU alone in advanced pancreatic carcinoma patients. *Ann Oncol*. 2004 ;15:467-73.

24. ZEGHARI-SQUALLI N, MISSET JL, CVITKOVIC E et al. Mechanism of the in vitro synergism between SN38 and oxaliplatin. *Proc Am Assoc Cancer Res* 1997;38:3.

25. HÉRON JF, FERVERS B, LATOUR JF, SOMMELET D, CHAUVERGNE J, PHILIP T. Standards, options et recommandations pour une bonne pratique en chimiothérapie. *Bull Cancer* 1995;82:823-34.

26. HÉRON JF, FERVERS B, LATOUR JF, SOMMELET D, CHAUVERGNE J, PHILIP T. Standards, options et recommandations pour une bonne pratique en chimiothérapie. In : Fédération Nationale des Centres de Lutte Contre le Cancer, ed. Recommandations pour la pratique clinique en cancérologie [cédérom]. Paris FNCLCC, John Libbey EUROTEXT, 1998.

27. FLEMING TR. One sample multiple testing procedure for phase II trials. *Biometrics* 1982 ;38 :143-15

28. BISSERY MC, VRIGNAUD P, LAVELLE F, et al: Experimental antitumor activity and pharmacokinetics of the camptothecin analog irinotecan (CPT-11) in mice. *Anticancer Drugs* 7:437-460, 1996

29. KORNMANN M, FAKLER H, BUTZER U, et al: Oxaliplatin exerts potent in vitro cytotoxicity in colorectal and pancreatic cancer cell lines and liver metastases. *Anticancer Res* 20:3259-3264, 2000

30. MANS DR, GRIVICICH I, PETERS GJ, et al: Sequence-dependent growth inhibition and DNA damage formation by the irinotecan-5-fluorouracil combination in human colon carcinoma cell lines. *Eur J Cancer* 35:1851-1861, 1999

31. MATSUOKA H, YANO K, Seo y, et al: Cytotoxicity of CPT-11 for gastrointestinal cancer cells cultured on fixed-contact-sensitive plates. *Anti-Cancer Drugs* 6:413-418, 1995

32. MULLANY S, SVINGEN PA, KAUFMANN SH, et al: Effect of adding the topoisomerase I poison 7-ethyl-10-hydroxycamptothecin (SN-38) to 5-fluorouracil and folinic acid in HCT-8 cells: elevated dTTP pools and enhanced cytotoxicity. Cancer *Chemother Pharmacol* 42:391-399, 1998

33. PAVILLARD V, FORMENTO P, ROSTAGNO P, et al: Combination of irinotecan (CPT11) and 5-fluorouracil with an analysis of cellular determinants of drug activity. *Biochem Pharmacol* 56:1315-1322, 1998

34. TAKEDA S, SHIMAZOE T, KUGA H, et al: Camptothecin analog (CPT-11)-sensitive human pancreatic tumor cell line QGP-1N shows resistance to SN-38, an active metabolite of CPT-11. *Biochem Biophys Res Commun* 188:70-77, 1992

35. ZEGHARI-SQUALLI N, RAYMOND E, CVITKOVIC E, et al: Cellular pharmacology of the combination of the DNA topoisomerase I inhibitor SN-38 and the diaminocyclohexane platinum derivative oxaliplatin. *Clin Cancer Res* 5:1189-1196, 1999

36. KRETZSCHMAR A, PINK D, THUSS-PATIENCE P, DORKEN B, REICHERT P, ECKERT R., Extravasations of oxaliplatin.*J Clin Oncol*. 21:4068-92, 003.

37. CAUSSANEL JP, LEVI F, BRIENZA S, MISSET JL, ITZHAKI M, ADAM R, MILANO G, HECQUET B, MATHÉ G. Phase I trial of 5-day continuous venous infusion of oxaliplatin at circadian rhythm-modulated rate compared with constant rate. *J Natl Cancer Inst*. 1990; 82 :1046-50.

38. FAYERS P, WEEDEN S, CURRAN D. EORTC QLQ-C30 reference values. Brussels: EORTC Quality of Life Study Group, 1998

39. AARONSON NK, AHMEDZAI S, BERGMAN B, et al: The European Organization for Research and Treatment of Cancer QLQ-C30: a quality-of-life instrument for use in international clinical trials in oncology. *J Natl Cancer Inst* 85:365-376, 1993

40. BERLIN JD, CATALANO P, THOMAS JP, et al: Phase III study of gemcitabine in combination with Fluorouracil versus Gemcitabine alone in patients with advanced pancreatic carcinoma : Eastern Cooperative Oncology Group trial E2297. *J Clin Oncol* 20:3270-3275, 2002

41. BRAMHALL SR, SCHULZ J, NEMUNAITIS J, et al: A double-blind placebo-controlled, randomised study comparing gemcitabine and marimastat with gemcitabine and placebo as first line therapy in patients with advanced pancreatic cancer. *Br J Cancer* 87:161-167, 2002

42. COLUCCI G, GIULANI F, GEBBIA V, et al: Gemcitabine alone or with cisplatin for the treatment of patients with locally advanced and/or metastatic pancreatic carcinoma : a prospective, randomized phase III study of the Gruppo Oncologia del l'Italia Meridionale. *Cancer* 94:902-910, 2002

43. FAYERS P, AARONSON N, BJORDAL K, et al: EORTC QLQ-C30 Scoring Manual Brussels: EORTC 1995

44. HEINEMANN V, QUIETZSCH D, GIESELER F, et al: A phase III trial comparing gemcitabine plus cisplatin vs gemcitabine alone in advanced pancreatic carcinoma. Proc Am Soc *Clin Oncol* 22:250, 2003 (abstr)

45. KLAPDOR R, FENNER C. Irinotecan (Campto R): efficacy as third/fourth line therapy in advanced pancreatic cancer. *Anticancer Res* 20:5209-5212, 2000

46. LOUVET CH, LABIANCA R, HAMMEL P, et al: Gemcitabine versus GEMOX (Gemcitabine + Oxaliplatin) in non resectable pancreatic adenocarcinoma: a GERCOR/GISCAD Intergroup Phase III. *Eur J Cancer 1 Suppl5*:S18, 2003 (abstr).

47. MOORE MJ, HAMM J, DANCEY J, et al: Comparison of gemcitabine versus the matrix metalloproteinase inhibitor BAY 12-9566 in patients with advanced or metastatic adenocarcinoma of the pancreas: a phase III trial of the National Cancer Institute of Canada clinical trials group. *J Clin Oncol* 21:3296-3302, 2003

48. ROCHA LIMA CMS, ROTCHE R, JEFFERY M, et al: A randomized phase 3 study comparing efficacy and safety of gemcitabine (GEM) and irinotecan (I), to GEM alone in patients (pts) with locally advanced or metastatic pancreatic cancer who have not received prior systemic therapy. *Proc Am Soc Clin Oncol* 22:251, 2003 (abstr)

49. SCHEITHAUER W, SCHULL B, ULRICH-PUR H, et al.Biweekly high-dose gemcitabine alone or in combination with capecitabine in patients with metastatic pancreatic adenocarcinoma: a randomized phase II trial. *Ann Oncol*.14:97-104, 2003

50. MAGAGNOLI M, MASCI G, CARNAGHI C, ZUCALI PA, CASTAGNA L, MORENGHI E, SANTORO A : Minidose warfarin is associated with a high incidence of International Normalized Ratio elevation during chemotherapy with FOLFOX regimen. *Ann Oncol*. 14:959-60, 2003

51. TAS F, AYKAN F, ALICI S, KAYTAN E, AYDINER A, TOPUZ E : Prognostic factors in pancreatic carcinoma : serum LDH levels predict survival in metastatic disease. *Am J Clin Oncol*. 24 : 547-50 , 2001.

52. KAPLAN EL, MEIER P : Nonparametric estimation from incomplete observations. *J Am Statist Ass.* 53 : 457-481, 1958.

ANNEXES

ANNEXE 1 :
Modalités de préparation et d'administration des produits

1 – IRINOTECAN (CAMPTO®)

L'irinotécan est un dérivé hémisynthétique de la camptothécine. Il s'agit d'un agent antinéoplasique qui agit comme inhibiteur spécifique de l'ADN topo-isomérase I. L'irinotécan est métabolisé par la carboxylestérase dans la plupart des tissus en un métabolite actif, le SN-38 qui s'est révélé plus actif que l'irinotécan sur la topo-isomérase I purifiée et plus cytotoxique sur plusieurs lignées de cellules tumorales murines ou humaines. L'inhibition de l'ADN topo-isomérase I par l'irinotécan ou le SN-38 induit des lésions simple-brin de l'ADN qui bloquent la fourche de réplication de l'ADN et sont responsables de l'activité cytotoxique. Celle-ci est fonction du temps du contact avec les cellules et est spécifique de la phase S.

Présentation
Solution à diluer pour perfusion, concentrée dans des flacons contenant 40 mg et 100 mg dans 2 ml et 5 ml respectivement, fournissant une solution à 20 mg/ml.

Préparation
Pour préparer la solution de Campto®, prélever, à l'aide d'une seringue graduée, dans le flacon la quantité voulue de la solution de Campto® en veillant à respecter les conditions d'asepsie et l'injecter dans une poche ou un flacon de perfusion de 250 ml contenant soit une solution de chlorure de sodium à 0,9 %, soit une solution glucosée à 5 %. Mélanger soigneusement la solution à perfuser par rotation manuelle.

Conservation
Le produit doit être conservé à l'abri de la lumière.
Après reconstitution, la solution de Campto® ne contenant pas de conservateur, elle doit être utilisée immédiatement après dilution. Cependant, si la dilution est réalisée sous stricte condition aseptique, la solution de Campto® pour perfusion peut être utilisée (temps de perfusion compris) dans les 12 heures, conservée à température ambiante, ou dans les 24 heures, conservée au réfrigérateur (+ 2 ^{o} à + 8 ^{o}C) après la préparation.

2 – OXALIPLATINE (ELOXATINE®)

L'oxaliplatine est un agent antinéoplasique, appartenant à une nouvelle classe de platine dans laquelle l'atome de platine est complexé avec un 1,2 diaminocyclohexane (DACH) et un groupe oxalate. L'oxaliplatine est un énantiomère unique, le cis-[oxalato (trans 1-1-1,2-DACH) platine]. L'oxaliplatine présente un large spectre d'activité cytotoxique in vitro et antitumoral in vivo dans divers systèmes de modèles tumoraux, y compris des modèles de cancer colorectal de l'homme. L'oxaliplatine s'est aussi montré efficace, in vitro et in vivo, dans différentes lignées résistant au cisplatine. Une action cytotoxique synergique avec le 5-fluorouracile a été mise en évidence in vitro et in vivo. Les études sur le mécanisme d'action, bien que celui-ci ne soit pas totalement élucidé, montrent que les dérivés hydratés résultant de la biotransformation de l'oxaliplatine interagissent avec l'ADN pour former des ponts intra- et inter-brins entraînant une interruption de la synthèse de l'ADN, cause de l'activité cytotoxique et antitumorale.

Présentation
solution concentrée pour perfusion dosée à 50 mg et à 100 mg,

Préparation
Reconstitution dans de l'eau ppi ou une solution de glucose à 5%, d'une solution contenant 5 mg d'oxaliplatine par ml.
Pour obtenir une concentration en oxaliplatine de 5 mg/ml, ajouter 10 ml de solvant à la solution concentrée à 50 mg, et 20 ml de solvant à la solution concentrée à 100 mg.

Conservation
Dans les conditions d'utilisation, la stabilité physicochimique a été démontrée pendant 48 heures à une température comprise entre + 2 °C et + 8 °C, et à + 30 °C. D'un point de vue microbiologique, la solut ion reconstituée doit être diluée immédiatement dans une solution de glucose à 5 %. Si la dilution n'est pas effectuée immédiatement, les durées et conditions de conservation avant utilisation sont sous la responsabilité de l'utilisateur ; la durée de conservation ne doit normalement pas excéder 24 heures à une température comprise entre + 2 °C et + 8 °C, sauf si la reconst itution a été réalisée dans des conditions aseptiques contrôlées et validées.

3 – 5-FLUOROURACILE

Le Fluorouracile est un antinéoplasique cytostatique de la classe des antimétabolites (antipyrimidine). Afin de mieux comprendre l'activité du fluorouracile, il faut rappeler que l'uracile joue un double rôle fondamental dans les tissus à croissance rapide : d'une part, en étant le précurseur (via la thymidilate synthétase) de la thymine, base nécessaire à la synthèse d'ADN qui préside à la division cellulaire ; d'autre part, en entrant dans la composition des ARNs qui président à la synthèse des protéines et des enzymes cellulaires.

Présentation
Solution à diluer pour perfusion concentrée dans des flacons contenant 250 mg, 500 mg, 1g et 5 g, dans 5 ml, 10ml, 20 ml et 100 ml respectivement, fournissant une solution à 50 mg/ml.

Mode d'administration
Voie intraveineuse. Ne pas administrer par voie intramusculaire. En cas d'extravasation, l'administration sera interrompue immédiatement.
Dilutions : 15 ml de solution injectable peuvent être mélangées à 250 ml des solutions suivantes :
- chlorure de sodium à 0,9%
- glucose à 5%
- glucose à 10%
- glucose à 2,5% + chlorure de sodium à 0,45%
- solution de Ringer
- solution de Hartmann

Conservation
A conserver à une température comprise entre + 15℃ et +25℃. Ce médicament est sensible à la lumière. Conserver le conditionnement primaire dans l'emballage extérieur.
Après dilution, une utilisation immédiate est recommandée. Toutefois, la stabilité a été démontrée pendant 8 heures à une température comprise entre + 15℃ et + 25℃.

4 - ACIDE L-FOLINIQUE

Antianémique (B : sang et organes hématopoïétiques). L'acide folinique sous sa forme lévogyre représente la forme active du DL-acide folinique. Une dose d'isomère L correspond à la moitié de la dose du composé racémique DL. L'efficacité et les effets indésirables de l'isomère L sont identiques à ceux du composé racémique.
L'acide folinique est un facteur antianémique dérivé de l'acide folique, dont il représente le métabolite actif. C'est un antagoniste biochimique des agents antifoliques tels que le méthotrexate (dont il est l'inhibiteur spécifique), mais aussi de la pyriméthamine et, dans une moindre mesure, la salazopyrine.

Présentation
Le produit est fourni sous forme de lyophilisat pour usage parentéral dosé à 25 mg, et sous forme de solution injectable par voie IM ou IV dosée à 25 mg/2,5 ml.

Conservation
Le lyophilisat est à conserver à une température inférieure à 30 ℃, à l'abri de la lumière et de l'hu midité. Après reconstitution : la solution peut être conservée 24 heures à une température inférieure à 30 ℃. La solution se conserve au réfrigérateur à une température comprise entre 2℃ et 8℃ et à l'abri de la lumière.

5 – GEMCITABINE (GEMZAR®)

Présentation
La gemcitabine est fournie sous forme de poudre lyophilisée conditionnée dans des flacons stériles contenant 200 mg ou 1 g d'hydrochlorure de gemcitabine (exprimée sous forme de base libre), du mannitol et de l'acétate de sodium.

Préparation
Le diluant recommandé pour la reconstitution du Gemzar® est une solution de chlorure de sodium à 0,9%.
Les flacons seront reconstitués grâce à l'ajout de sérum salé isotonique pour aboutir à une solution contenant idéalement 10 mg/ml au maximum. La concentration pour les flacons de 200mg et de 1g ne doit pas dépasser 40 mg/ml.

Administration
Une quantité appropriée du produit sera préparée avec du sérum salé isotonique et administrée en perfusion continue pendant 30 minutes.

Conservation
Le produit lyophilisé doit être stocké à moins de 3℃. Une fois le produit reconstitué, il doit être s tocké à température ambiante et utilisé dans les 24 heures. Ne pas stocker le produit reconstitué dans le réfrigérateur.

ANNEXE 2 :
Tableau récapitulatif des investigations

Plan de traitement													
**Date**	**J1**	**J8**	**J15**	**J22**	**J29**	**J36**	**J43**	**J50**	**J57**	**J64**	**J71**	**J78**	**Jn**
**Bras A : FOLFIRINOX**	**C1**		**C2**		**C3**		**C4**		**C5**		**C6**		**Cn**
	oxaliplatine 85 mg/m² J1+ irinotecan 180 mg/m² J1+ acide folinique 400 mg/m² J1, 5-FU bolus 400 mg/m² J1, 5-FU continu 2,4 g/m² sur 46 heures J1/ J2 de chaque cycle								*Reprise du traitement selon le schéma précédent, toutes les 2 semaines*				
**Bras B : gemcitabine**	↑	↑	↑	↑	↑	↑	↑		↑	↑	↑		↑
	gemcitabine 1000 mg/m² de chaque semaine pendant 7 semaines								*Reprise de la gemcitabine 3 semaines sur 4*				

12 cycles prévus pour le bras A = 6 mois de traitement

Plan de surveillance BRAS A Folfirinox									
**Visite**	**Bilan inclusion**	**Bilans de suivi**				**Bilan à 8 semaines**	**Bilans de suivi après reprise**		
**N° de visite**	**V0**	**V1**	**V2**	**V3**	**V4**	**V5**	**V6**	**V7**	**Vn**
**Date**	**J0**	**J1**	**J15**	**J29**	**J43**	**Autour J50**	**J57**	**J71**	**Jn**
Consentement éclairé signé	**X**								
Critères d'inclusion / non inclusion	**X**								
Randomisation	**X**								
**Examen clinique**									
Poids	**X**		**X**	**X**	**X**	**X**	**X**	**X**	**X**
Taille Surface.corporelle	**X**								
Etat général / OMS	**X**		**X**	**X**	**X**	**X**	**X**	**X**	**X**
Traitements concomitants	**X**								
**Toxicités (2)**									
Evaluation de la tolérance			**X**	**X**	**X**	**X**	**X**	**X**	**X**
**Examen biologique** (4)									
NFS, plaquettes	**X**	**X**	**X**	**X**	**X**	**X**	**X**	**X**	**X**
TP,TCK	**X**								
Ionogramme, Ca	**X**		**X**	**X**	**X**	**X**	**X**	**X**	**X**
Protidémie	**X**		**X**	**X**	**X**	**X**	**X**	**X**	**X**
Albuminémie, LDH	**X**								
Bilan hépatique	**X**		**X****	**X****	**X****	**X**	**Examen tous les deux mois**		
Creatininémie	**X**	**X**	**X**	**X**	**X**	**X**	**Examen tous les deux mois**		
Glycémie	**X**		**X**	**X**	**X**	**X**	**X**	**X**	**X**
ACE, Ca 19-9 *	**X**					**X**	**Examen tous les deux mois**		
Test de grossesse	**X**(1)								
**Bilan paraclinique**(3)									
Scanner Thoracoabdominopelvien	**X**					**X**	**Examen scanographique tous les 2 mois**		
ECG	**X**								
**Questionnaire de Qualité de Vie**	**X**		**X**	**X**	**X**	**X**	**X**	**X**	**X**

6 mois de traitement prévus pour le bras B

Plan de surveillance BRAS B GEMCITABINE													
**Visite**	**Bilan inclusion**	**Bilans de suivi**							**Bilan à 8 semaines**	**Bilans de suivi après reprise**			
**N°de visite**	**V0**	**V1**	**V2**	**V3**	**V4**	**V5**	**V6**	**V7**	**V8**	**V9**	**V10**	**V11**	**Vn**
**Date**	**J0**	**J1**	**J8**	**J15**	**J22**	**J29**	**J36**	**J43**	**Autour J50**	**J57**	**J64**	**J71**	**Jn**
Consentement éclairé signé	**X**												
Critères d'inclusion / non inclusion	**X**												
Randomisation	**X**												
**Examen clinique**	**X**												
Poids	**X**	**X**		**X**		**X**		**X**	**X**	**X**		**X**	
Taille Surface.corporelle	**X**												
Etat général / OMS	**X**	**X**		**X**		**X**		**X**	**X**	**X**		**X**	
Traitements concomitant	**X**												
**Toxicités (2)**													
Evaluation de la tolérance			**X**	**X**	**X**	**X**	**X**	**X**	**X**	**X**	**X**	**X**	**X**
**Examen biologique** (4)													
NFS, plaquettes	**X**	**X**	**X**	**X**	**X**	**X**	**X**	**X**	**X**	**X**	**X**	**X**	**X**
TP,TCK	**X**												
Ionogramme, Ca	**X**			**X**		**X**		**X**	**X**	**X**		**X**	
Protidémie	**X**								**X**				
Albuminémie, LDH	**X**												
Bilan hépatique	**X**			**X**		**X**		**X**	**X**	**Examen tous les deux mois**			
Créatininémie	**X**			**X**		**X**		**X**	**X**	**Examen tous les deux mois**			
Glycémie	**X**			**X**		**X**		**X**	**X**	**X**		**X**	
ACE, Ca 19-9*	**X**								**X**	**Examen tous les deux mois**			
Test de grossesse	**X**(1)												
**Bilan paraclinique**(3)													
Scanner Thoracoabdomino pelvien	**X**								**X**	**Examen scanographique tous les 2 mois**			
ECG	**X**												
**Questionnaire de Qualité de Vie**	**X**			**X**		**X**		**X**	**X**	**X**		**X**	

**seul le marqueur le plus performant sera dosé ultérieurement, choisir le marqueur qui a la valeur initiale la plus importante par rapport à la normale*
*** Bilan hépatique avec Bilirubine totale, libre et conjuguée doit être fait au moins tous les mois*
(1) pour les femmes en âge de procréer sans moyen contraceptif efficace
(2) Evénements Indésirables Graves (EIG) à déclarer dans les 48 heures au promoteur jusqu'à 30 jours après la fin de la dernière cure de chimiothérapie. Au delà de cette période seuls les EIG susceptibles d'être dus à la recherche seront déclarés au promoteur dès lors qu'aucune autre cause que la recherche ne peut raisonnablement être attribuée.
(3) Le bilan radiologique peut être effectué au cours des 3 semaines précédant l'inclusion.
(4) Le bilan biologique doit être effectué au plus tard dans la semaine précédant l'inclusion.

Les patients sans progression ayant eu les 6 mois de traitements seront suivis tous les 2 mois. Seront évalués la qualité de vie avec remise du questionnaire EORTC QLQ-C30 et la progression de la maladie, grâce à l'imagerie.

Les patients ayant progressé seront suivis tous les 6 mois jusqu'au décès. Seront évalués : les effets toxiques à long terme et la survie. Si un autre traitement est établi, il devra être rapporté.

ANNEXE 3 :
Evaluation de l'état général en fonction de l'échelle de l'ECOG ou de l'OMS

**ETAT GENERAL**<br>**ECOG-ZUBROD/OMS**	**ECHELLE**
Activité normale, sans restriction.	**0**
Restreint pour des activités physiques importantes mais patient ambulatoire et capable de fournir un travail léger.	**1**
Ambulatoire et capable de se prendre en charge, mais incapable de fournir un travail pendant plus 50% de son temps.	**2**
Capacité de prise en charge propre beaucoup plus limitée. Passe plus de 50 % de son temps au lit ou dans une chaise.	**3**
Complètement grabataire.<br>Incapable de se prendre en charge. Le patient reste totalement couché au lit ou sur une chaise.	**4**

ANNEXE 4 :
Classification des évaluations tumorales
Classification RECIST (JNCI, 2/2/2000)

1. Mesure des lésions tumorales

1.1 Définitions

Les lésions tumorales seront réparties en deux grandes catégories :

Lésions mesurables : lésions qui peuvent être mesurées avec précision dans au moins une dimension (le plus grand diamètre étant reporté) soit ≥ à 20 mm avec un scanner conventionnel soit ≥ à 10 mm avec un scanner spiralé.

Lésions non mesurables : toutes les autres lésions comprenant les petites lésions de plus grands diamètres < à 20 mm avec un scanner conventionnel ou < à 10 mm avec un scanner spiralé et les véritables lésions non mesurables.

Toutes les mesures doivent être reportées dans le système métrique en utilisant une règle ou les calipers. Les évaluations de départ doivent être réalisées avec un délai le plus court possible (sans excéder 4 semaines) par rapport au début du traitement.

Les lésions qui sont considérées comme véritablement non mesurables sont les suivantes :

- Lésions osseuses
- Atteinte méningée
- Ascite
- Epanchement pleural et/ou péricardique
- Sein inflammatoire
- Lymphangite cutanée et/ou pulmonaire
- Masses abdominales qui ne sont pas confirmées ou suivies par une technique d'imagerie
- Lésion kystique

Les lésions tumorales situées en territoire irradié peuvent être considérées comme mesurables ou non selon les conditions définies dans le protocole.

1.2 Méthodes de mesure

La méthode de mesure et la technique utilisées pour les mesures des lésions doivent être identiques au début de l'étude et pendant le suivi. Les évaluations par imagerie sont préférées aux évaluations cliniques quand les deux méthodes ont été utilisées pour déterminer l'activité anti-tumorale du traitement.

Lésions cliniques
Les lésions cliniques seront considérées comme mesurables lorsqu'elles sont superficielles (nodule cutané, ganglion lymphatique palpable). Pour les lésions cutanées, une documentation par photographie couleur comportant une règle pour évaluer la taille de la lésion est recommandée.

Radiographie pulmonaire
Les lésions visibles sur les radiographies pulmonaires sont considérées comme mesurables lorsqu'elles sont clairement identifiées et cernées par de l'air pulmonaire. Toutefois le scanner est préférable.

Scanner et IRM
Scanner et IRM sont actuellement les techniques les plus reproductibles pour mesurer les lésions cibles et évaluer une réponse. Scanner conventionnel et IRM sont réalisés avec des coupes jointives d'au moins 10 mm d'épaisseur. Les scanners spiralés doivent être réalisés en utilisant des coupes jointives de 5 mm.
Cela s'applique au thorax, abdomen et pelvis. Tête et cou et les extrémités nécessitent des protocoles spécifiques.

Echographie
Lorsque le but de l'étude est l'évaluation d'une réponse objective, l'échographie ne doit pas être utilisée pour mesurer les lésions tumorales qui ne sont pas cliniquement d'accès facile.
L'échographie peut être une alternative à l'examen clinique pour mesurer des ganglions superficiels palpables, des lésions sous-cutanées et des nodules thyroïdiens. L'échographie peut être utilisée pour confirmer une disparition complète de lésions superficielles évaluées par examen clinique.

Endoscopie, laparoscopie
L'utilisation de ces techniques pour évaluer une réponse objective n'est pas complètement validée. Leur utilisation dans ce contexte spécifique nécessite des équipements sophistiqués et un niveau d'expertise qui n'est disponible que dans quelques centres.
Leur utilisation pour évaluer des réponses objectives doit être restreinte à la validation d'études dans des centres de référence. Toutefois de telles techniques peuvent être utilisées pour confirmer une réponse totale sur le plan pathologique lorsque des biopsies sont réalisées.

Marqueurs tumoraux
Les marqueurs tumoraux ne peuvent être utilisés seuls pour évaluer une réponse.
Si les marqueurs sont initialement au-dessus de leur valeur normale, ils doivent se normaliser lorsque le patient est considéré en réponse complète et lorsque toutes les lésions tumorales ont disparu.
Des critères additionnels de standardisation de l'utilisation du PSA et du CA-125 sont actuellement en cours de validation et peuvent être utilisés dans les essais cliniques.

Cytologie et histologie
Dans de rares cas, ces techniques peuvent être utilisées pour différencier une réponse partielle et une réponse complète. Par exemple dans les tumeurs germinales où un résidu bénin peut persister.
La confirmation cytologique de l'origine néoplasique d'un épanchement qui apparaît ou s'aggrave durant le traitement est nécessaire pour différencier réponse, stabilité et progression tumorale lorsqu'une tumeur mesurable est un critère nécessaire pour apprécier la réponse tumorale (un épanchement peut être un effet secondaire du traitement).

2. Evaluation de la réponse tumorale

2.1 Evaluation à l'inclusion

- **Détermination de la réponse tumorale globale et des lésions mesurables**

Pour évaluer une réponse objective**,** il faut apprécier la masse tumorale au début et en faire des mesures comparatives dans le suivi.
Seuls les patients présentant une maladie mesurable à l'inclusion peuvent être inclus dans des protocoles où la réponse tumorale est le critère principal d'évaluation. Une maladie mesurable est définie par la présence d'au moins une lésion mesurable. Si la maladie mesurable se limite à une lésion unique, sa nature néoplasique doit être confirmée par cytologie/histologie.

- **Documentation des lésions cibles/non cibles à l'inclusion**

Toutes les lésions mesurables et jusqu'à un maximum de 5 lésions par organe et de 10 au total, représentatives de tous les organes atteints doivent être considérées comme des lésions cibles, être notées et mesurées à l'inclusion. Les lésions cibles doivent être sélectionnées en fonction de leur taille (plus grand diamètre) et de la possibilité d'être mesurées avec précision de façon reproductible (soit par imagerie soit cliniquement). La somme des plus grands diamètres de toutes les lésions cibles calculées à l'inclusion sera utilisée comme référence pour objectiver une réponse tumorale. Toutes les autres lésions ou sites de la maladie doivent être notés et identifiés comme lésions non cibles à l'inclusion. Il n'est pas nécessaire de les mesurer. Dans le suivi, elles seront notées présentes ou absentes.

2.2 Critères de réponse

- **Evaluation des lésions cibles**

Réponse complète (RC) : disparition de toutes les lésions cibles.
Réponse partielle (RP) : diminution d'au moins 30% de la somme des plus grands diamètres des lésions cibles par rapport à la somme des plus grands diamètres des lésions cibles à l'inclusion.
Progression (MP) : augmentation d'au moins 20% de la somme des plus grands diamètres des lésions cibles en prenant comme référence la plus petite valeur de la somme des plus grands diamètres reportés depuis le début du traitement ou bien apparition d'une ou de plusieurs nouvelles lésions.
Maladie stable (MS) : pas de diminution ou d'augmentation suffisante pour entrer dans le cadre d'une réponse partielle ou d'une progression en référence à la plus petite somme des plus grands diamètres depuis le début du traitement.

- **Evaluation des lésions non cibles**

Réponse complète : disparition de toutes les lésions non cibles et normalisation des marqueurs tumoraux.
Réponse incomplète et maladie stable : Persistance de une ou plusieurs lésions non cibles et/ou persistance des marqueurs tumoraux au-dessus des valeurs normales.
Progression : apparition de une ou plusieurs nouvelles lésions. Progression non équivoque des lésions non cibles existantes.

- **Evaluation de la meilleure réponse globale**

La meilleure réponse globale est la meilleure réponse enregistrée depuis le début du traitement jusqu'à l'apparition d'une récidive ou d'une progression (en prenant comme référence pour la progression la plus petite mesure rapportée depuis le début du traitement).

En général, la meilleure réponse du patient dépendra de l'ensemble des mesures et de la confirmation des critères définis dans le paragraphe 2.2.

Lésions cibles	Lésions non cibles	Nouvelles lésions	Réponse globale
RC	RC	non	RC
RC	Réponse incomplète et maladie stable	non	RP
RP	non - MP	non	RP
MS	non - MP	non	MS
Si une lésion cible ou non cible progresse ou s'il y a apparition d'une nouvelle lésion, l'évaluation de la réponse globale est la progression de la maladie.			

Les patients présentant une altération de l'état général nécessitant l'interruption du traitement sans progression objective évidente de la maladie, doivent à ce moment être notés détérioration symptomatique. Le maximum doit être fait pour objectiver la progression même après arrêt du traitement.

Dans certaines circonstances, il peut être difficile de distinguer une maladie résiduelle du tissu normal. Quand cette distinction intervient dans l'évaluation d'une réponse complète, il est recommandé d'explorer la lésion résiduelle par biopsie et/ou cytologie à l'aiguille avant de confirmer la réponse complète.

2.3 Confirmation de la mesure/ durée de la réponse

- **Confirmation**

Pour donner le statut de réponse partielle ou de réponse complète, les modifications de mesure des tumeurs doivent être confirmées par de nouvelles mesures 4 semaines au moins après que la première réponse ait été observée.

Dans le cas de maladie stable, les mesures ne peuvent répondre à ce critère qu'au moins une fois après l'entrée dans l'étude pendant un intervalle de temps minimum (en général pas moins de six à huit semaines).

- **Durée de la réponse globale**

La durée de la réponse globale est déterminée depuis le moment où une réponse complète / réponse partielle est obtenue (la première rapportée) jusqu'au moment où une récidive/progression est objectivement documentée.

La durée de la réponse complète est déterminée depuis le moment où la réponse complète est obtenue jusqu'au moment où une récidive objective est documentée.

- **Durée d'une maladie stable**

Une maladie stable est déterminée à partir du début du traitement jusqu'à ce qu'un critère de progression soit rencontré en prenant comme référence le plus petit total des mesures rapportées depuis le début du traitement.

La pertinence clinique de la durée d'une maladie stable varie selon les types de tumeur et leur grade. Toutefois, il est recommandé de spécifier dans le protocole l'intervalle de temps minimum requis entre deux mesures pour déterminer la stabilité de la maladie. Cet intervalle de temps doit prendre en compte le bénéfice clinique attendu qu'un tel statut peut apporter à la population étudiée.

2.4 Survie sans progression

Ce document concerne en premier lieu l'utilisation des réponses objectives. Dans certains cas (tumeurs cérébrales, essais de drogues anticancéreuses), l'évaluation de la réponse tumorale peut ne pas être la méthode optimale pour estimer l'activité potentielle anti-tumorale de nouveaux agents/régimes. Dans de tels cas, la survie sans progression peut être considérée comme une alternative valable pour fournir une estimation initiale de l'effet biologique de nouveaux agents qui peuvent agir par mécanisme non cytotoxique. Il est clair que dans un essai non contrôlé proposant d'utiliser la survie sans progression, il sera nécessaire de documenter avec prudence les données pour estimer la survie sans progression attendue en l'absence de traitement efficace. Il est aussi recommandé que l'analyse soit tout à fait prudente en fonction de la probabilité de confusion des biais concernant par exemple la sélection ou l'estimation. Des essais non contrôlés utilisant la survie sans progression comme objectif primaire doivent être envisagés sur la base du cas par cas et la méthodologie à appliquer doit être décrite parfaitement dans le protocole.

2.5 Comité de relecture

Pour les études où le taux de réponse est l'objectif primaire, il est fortement recommandé que toutes les réponses soient revues par un expert indépendant de l'étude. Une revue simultanée du dossier des patients et des images radiologiques est la meilleure approche.

ANNEXE 5 :
Extrait des critères de toxicité (CTC–NCI)
Version du 31/05/2003

ANNEXE 6 :
Formulaire de notification d'un événement indésirable grave

NOTIFICATION D'UN EVENEMENT INDESIRABLE GRAVE
A faxer à la pharmacovigilance du BECT n°+ 33 (0)1 44 23 55 70

beCT F.N.C.L.C.C. Centre d'Investigation Clinique en Cancérologie

N°EudraCT : … … … … … … … … … … … … **N°Protocole :** … … … … … … … … … **Pays :**… … … … … … … … … … .

❒ **Evénement Indésirable Grave ATTENDU**	❒ **Evénement Indésirable Grave INATTENDU**
❒ **Rapport initial** ❒ **Rapport de suivi n°**: |__|	**Centre investigateur :** ....

(1) Selon le RCP (Vidal) ou BI (version la plus récente)

1. INFORMATIONS PATIENT

N° inclusion : |___________|	Nom (3 lettres) : |__|__|__|	Prénom (2 lettres) : |__|__|	Date de naissance : |__|__| / |__|__| / |__|__|__|__|
Sexe : ❒ F ❒ M	Poids (kg) : |_______|	Taille (cm) : |_______|	Bras de traitement : |____|

2. INFORMATIONS SUR L'EVENEMENT

Date de survenue événement : \|__\|__\| / \|__\|__\| / \|__\|__\| **Toxicité (grade NCI – CTC V3):** □ 1 □ 2 □ 3 □ 4 □ 5

Diagnostic ou principaux symptômes… … .....
… ..

3. TYPE D'EVENEMENT

- ❒ Décès … date \|__\|__\| / \|__\|__\| / \|__\|__\|
- ❒ Mise en jeu du pronostic vital
- ❒ Hospitalisation (> 24h) ou prolongation d'hospitalisation) date \|__\|__\| / \|__\|__\| / \|__\|__\|
- ❒ Médicalement significatif, préciser : …
- ❒ Invalidité / Incapacité temporaire ou permanente
- ❒ Autre cancer : ....
- ❒ Anomalie congénitale ou malformation fœtale

4. ÉVOLUTION

- ❒ Evénement en cours
- ❒ Résolution sans séquelle, date \|__\|__\| / \|__\|__\| / \|__\|__\|
- ❒ Résolution avec séquelles, date \|__\|__\| / \|__\|__\| / \|__\|__\|

Nature des séquelles : ... …

- ❒ Décès en relation avec l'événement
- ❒ Décès sans rapport avec l'événement
- ❒ Inconnu

Date de fin d'hospitalisation : \|__\|__\| / \|__\|__\| / \|__\|__\|

5. TRAITEMENTS

		Dates		Doses et Unites		Imputabilite
**Chimiotherapie, radiotherapie..**	**Voie**	**Dates de traitement avant apparition de l'événement**		**Dernière dose administrée**	**Dose cumulative depuis la 1ère administration**	**1 : Exclu<br>2 : Douteux<br>3 : Plausible<br>4 : Vraisemblable<br>5 : Très vraisemblable<br>6 : Ne peut conclure**
1.		Du|__|__||__|__||__|__|	au|__|__||__|__||__|__|			|__|
2.		Du|__|__||__|__||__|__|	au|__|__||__|__||__|__|			|__|
3.		Du|__|__||__|__||__|__|	au|__|__||__|__||__|__|			|__|
4.		Du|__|__||__|__||__|__|	au|__|__||__|__||__|__|			|__|
5.		Du|__|__||__|__||__|__|	au|__|__||__|__||__|__|			|__|

Un ou des traitements ont-ils été arrêtés ?

❒ Oui N° \|__\| N° \|__\| N° \|__\| N° \|__\| N°\|__\| ❒ Non ❒ NA

Disparition de l'événement après arrêt d'un ou des produits ?

❒ Oui ❒ Non ❒ NA

Un ou des traitements ont-ils été réintroduits ?

❒ Oui N° \|__\| N° \|__\| N° \|__\| N° \|__\| N°\|__\| ❒ Non ❒ NA

Réapparition de l'événement après réintroduction ?

❒ Oui ❒ Non ❒ NA

6. IMPUTABILITE GLOBALE *(Selon vous, cet événement est plutôt lié)*

- ❒ au(x) traitement(s) de l'essai (préciser le(s) nom(s) des traitements)… .... … … … ..... … … … … … … … …..... .
- ❒ au protocole de l'essai
- ❒ autre(s) traitement(s) concomitant(s)
- ❒ à la progression de la maladie
- ❒ autre(s) maladie(s) concomitante(s)
- ❒ autre(s) :… … … … … … … … … … … … … … … … … … .... … …

7. NOTIFICATEUR

Nom et fonction du notificateur :

Etablissement :
Adresse :
Tél. :
Fax :
E-mail :

Date \|__\|__\| / \|__\|__\| / \|__\|__\|

Signature de l'investigateur/co-investigateur

ANNEXE 6 bis:
Formulaire complémentaire d'information d'un EIG inattendu

COMPLEMENT D'INFORMATION
D'UN EVENEMENT INDESIRABLE GRAVE INATTENDU
A faxer à la pharmacovigilance du BECT n° + 33 (0)1 44 23 55 70

beCT F.N.C.L.C.C. Centre d'Investigati[illegible] Clinique e[illegible] Cancérolo[illegible]

N° EudraCT : … … … … … … … … … … … … … **N° Protocole :** … … … … … … … … … .. **Pays :**… … … … … … … … … …

❐ **Rapport de suivi n° :** |__| **Centre investigateur :** … … … … … … … … … …. … … …………

(1) Selon le RCP (Vidal) ou BI (version la plus récente)

1. INFORMATIONS PATIENT

N° inclusion : |___________|	Nom (3 lettres) : |__|__|__|	Prénom (2 lettres) : |__|__|	Date de naissance : |__|__| / |__|__| / |__|__|__|[illegible]
Sexe : ❐ F ❐ M	Poids (kg) : |_______|	Taille (cm) : |_______|	Bras de traitement : |____|

2. INFORMATIONS SUR L'EVENEMENT

Date de survenue événement : |__|__| / |__|__| / |__|__| **Toxicité (grade NCI – CTC V3):** ☐ 1 ☐ 2 ☐ 3 ☐ 4 ☐ 5

Diagnostic ou principaux symptômes…….. …

… …

3. NARRATIF

……

……

……

4. ÉVOLUTION

❐ Evénement en cours	❐ Décès en relation avec l'événement
❐ Résolution sans séquelle, date |__|__| / |__|__| / |__|__|	❐ Décès sans rapport avec l'événement
❐ Résolution avec séquelles, date |__|__| / |__|__| / |__|__|	❐ Inconnu
Nature des séquelles : …	Date de fin d'hospitalisation : |__|__| / |__|__| / |__|__|

5. TRAITEMENTS *(compléter le tableau ci-après en page 2/2)*

6. TRAITEMENT DE L'ÉVÉNEMENT INDÉSIRABLE

Traitement	Dose/Unité	Voie	Indication	Date début	Date fin	En cour[illegible]
… … … … … … … … … … .			… … … … … … … … …	|__|__| / |__|__| / |__|__|	|__|__| / |__|__| / |__|__|	❐
… … … … … … … … … … .			… … … … … … … … …	|__|__| / |__|__| / |__|__|	|__|__| / |__|__| / |__|__|	❐
… … … … … … … … … … .			… … … … … … … … …	|__|__| / |__|__| / |__|__|	|__|__| / |__|__| / |__|__|	❐

7. MÉDICATION CONCOMITANTE PERTINENTE *(à l'exclusion de celle utilisée pour traiter l'événement)*

Traitement	Dose/Unité	Voie	Indication	Date début	Date fin	En cours	Relatio[illegible] causal[illegible]	
							oui	non
… … … … … … … … … …			… … … … … … … … … …	|__|__| / |__|__| / |__|__|	|__|__| / |__|__| / |__|__|	❐	❐	❐
… … … … … … … … … …			… … … … … … … … … …	|__|__| / |__|__| / |__|__|	|__|__| / |__|__| / |__|__|	❐	❐	❐
… … … … … … … … … …			… … … … … … … … … …	|__|__| / |__|__| / |__|__|	|__|__| / |__|__| / |__|__|	❐	❐	❐

8. ANTÉCÉDENTS MÉDICAUX PERTINENTS ET/OU MALADIE(S) CONCOMITANTE(S)

… ….. … … .

… ….. … … .

9. IMPUTABILITE GLOBALE *(Selon vous, cet événement est plutôt lié)*

❐ au(x) traitement(s) de l'essai (*préciser le(s) nom(s) des traitements*)… ……… … … … ….. … … … … … … … ….. .	❐ à la progression de la maladie
❐ au protocole de l'essai	❐ autre(s) maladie(s) concomitante(s)
❐ autre(s) traitement(s) concomitant(s)	❐ autre(s)… … … … … … … … … … … … … … … … … ….. … …

10. NOTIFICATEUR

Nom et fonction du notificateur :<br>Etablissement :<br>Adresse :<br>Tél. :<br>Fax :<br>E-mail :	Date |__|__| / |__|__| / |__|__| Signature de l'investigateur/co-investigateur

COMPLEMENT D'INFORMATION D'UN EVENEMENT INDESIRABLE GRAVE INATTENDU (page 2/2)

A faxer à la pharmacovigilance du BECT n° + 33 (0)1 44 23 55 70

**N°Protocole :**	**N°EudraCT :**	**N°Patient :**

5. TRAITEMENTS

<table>
<tr><th></th><th>VOIE</th><th colspan="2">DATES</th><th colspan="2">DOSES ET UNITES</th><th>MODIFICATIONS DE TRAITEMENTS</th><th colspan="3">TRAITEMENTS</th></tr>
<tr><th></th><th></th><th colspan="2">Dates de traitement avant apparition de l'événement</th><th>Dernière dose administrée</th><th>Dose cumulative depuis la 1ère adm.</th><th>(1) Réduction de dose
(2) Interruption temporaire
(3) Interruption définitive</th><th>Disparition
EI après arrêt traitement
1 : Oui
2 : Non
3 : NA</th><th>Réapparition
EI après réintroduction
1 : Oui
2 : Non
3 : NA</th><th>Imputabilité :
1 : Exclu
2 : Douteux
3 : Plausible
4 : Vraisemblable
5 : Très vraisemblable
6 : Ne peut conclure</th></tr>
<tr><td>… … … … … … … … …</td><td></td><td>Du|__|__||__|__||__|__|</td><td>Au|__|__||__|__||__|__|</td><td>|__|__|__|__|</td><td>|__|__|__|__|</td><td>|__| |__|__||__|__||__|__|
Date</td><td>|__| |__|__||__|__||__|__|
Date</td><td>|__| |__|__||__|__||__|__|
Dose : |__|__|__|__|</td><td>|__|</td></tr>
<tr><td>… … … … … … … … …</td><td></td><td>Du|__|__||__|__||__|__|</td><td>Au|__|__||__|__||__|__|</td><td>|__|__|__|__|</td><td>|__|__|__|__|</td><td>|__| |__|__||__|__||__|__|
Date</td><td>|__| |__|__||__|__||__|__|
Date</td><td>|__| |__|__||__|__||__|__|
Dose : |__|__|__|__||__|</td><td>|__|</td></tr>
<tr><td>… … … … … … … … …</td><td></td><td>Du|__|__||__|__||__|__|</td><td>Au|__|__||__|__||__|__|</td><td>|__|__|__|__|</td><td>|__|__|__|__|</td><td>|__| |__|__||__|__||__|__|
Date</td><td>|__| |__|__||__|__||__|__|
Date</td><td>|__| |__|__||__|__||__|__|
Dose : |__|__|__|__|</td><td>|__|</td></tr>
<tr><td>… … … … … … … … …</td><td></td><td>Du|__|__||__|__||__|__|</td><td>Au|__|__||__|__||__|__|</td><td>|__|__|__|__|</td><td>|__|__|__|__|</td><td>|__| |__|__||__|__||__|__|
Date</td><td>|__| |__|__||__|__||__|__|
Date</td><td>|__| |__|__||__|__||__|__|
Dose : |__|__|__|__|</td><td>|__|</td></tr>
<tr><td>… … … … … … … … …</td><td></td><td>Du|__|__||__|__||__|__|</td><td>Au|__|__||__|__||__|__|</td><td>|__|__|__|__|</td><td>|__|__|__|__|</td><td>|__| |__|__||__|__||__|__|
Date</td><td>|__| |__|__||__|__||__|__|
Date</td><td>|__| |__|__||__|__||__|__|
Dose : |__|__|__|__|</td><td>|__|</td></tr>
<tr><td>… … … … … … … … …</td><td></td><td>Du|__|__||__|__||__|__|</td><td>Au|__|__||__|__||__|__|</td><td>|__|__|__|__|</td><td>|__|__|__|__|</td><td>|__| |__|__||__|__||__|__|
Date</td><td>|__| |__|__||__|__||__|__|
Date</td><td>|__| |__|__||__|__||__|__|
Dose : |__|__|__|__|</td><td>|__|</td></tr>
<tr><td>… … … … … … … … …</td><td></td><td>Du|__|__||__|__||__|__|</td><td>Au|__|__||__|__||__|__|</td><td>|__|__|__|__|</td><td>|__|__|__|__|</td><td>|__| |__|__||__|__||__|__|
Date</td><td>|__| |__|__||__|__||__|__|
Date</td><td>|__| |__|__||__|__||__|__|
Dose : |__|__|__|__|</td><td>|__|</td></tr>
<tr><td>… … … … … … … … …</td><td></td><td>Du|__|__||__|__||__|__|</td><td>Au|__|__||__|__||__|__|</td><td>|__|__|__|__|</td><td>|__|__|__|__|</td><td>|__| |__|__||__|__||__|__|
Date</td><td>|__| |__|__||__|__||__|__|
Date</td><td>|__| |__|__||__|__||__|__|
Dose : |__|__|__|__|</td><td>|__|</td></tr>
<tr><td>… … … … … … … … …</td><td></td><td>Du|__|__||__|__||__|__|</td><td>Au|__|__||__|__||__|__|</td><td>|__|__|__|__|</td><td>|__|__|__|__|</td><td>|__| |__|__||__|__||__|__|
Date</td><td>|__| |__|__||__|__||__|__|
Date</td><td>|__| |__|__||__|__||__|__|
Dose : |__|__|__|__|</td><td>|__|</td></tr>
</table>

ANNEXE 7 :
Formulaire d'information destiné au patient(1)

ETUDE RANDOMISEE DE PHASE II / III COMPARANT L'ASSOCIATION FOLFIRINOX [OXALIPLATINE / IRINOTECAN / LV5FU] A LA GEMCITABINE EN PREMIERE LIGNE DE CHIMIOTHERAPIE DE PATIENTS ATTEINTS D'UN CANCER DU PANCREAS METASTATIQUE

Promoteur : Fédération Nationale des Centres de Lutte Contre le Cancer (FNCLCC), 101 rue de Tolbiac, 75654 PARIS CEDEX 13
Investigateur Coordonnateur : Pr. Thierry CONROY, Département d'Oncologie Médicale, Centre Alexis Vautrin 6, avenue de Bourgogne, 54511 Vandoeuvre-lès-Nancy cedex

1. Quel est l'objectif de cette étude ?

Madame, Monsieur,

Votre médecin vous a informé que vous êtes atteint d'un cancer du pancréas qui nécessite un traitement. Il vous propose de participer à une étude dont le but est d'évaluer l'efficacité et la tolérance d'une chimiothérapie associant oxaliplatine, irinotecan et LV5FU (Folfirinox) par rapport à une chimiothérapie de référence par gemcitabine.

2. Combien de personnes participeront à cette étude ?

Cette étude nationale comparative de phase II est réalisée par plusieurs équipes de médecins en France. Elle doit inclure 88 patients sur une période de 2 ans. La moitié des patients recevront le traitement par l'association Folfirinox, l'autre moitié la gemcitabine. L'étude de phase III sera également réalisée par les mêmes médecins et inclura sur une période de 3 ans **360 patients**.

3. Quel est le déroulement de l'étude ?

Si vous acceptez de participer à cette étude, vous devrez être examiné par votre médecin et effectuer, une prise de sang, un scanner thoraco-abdominopelvien, un électrocardiogramme et compléter un questionnaire de qualité de vie. Après avoir réalisé ces examens et s'il n'y a pas de contre indication à l'un des traitements, vous recevrez l'un de ces traitements, le choix étant décidé par tirage au sort :

-Soit un traitement par **gemcitabine**, un médicament potentiellement efficace et bien toléré de chimiothérapie. Il s'agit d'un des traitements de référence de votre maladie.

-Soit un traitement plus nouveau, appelé **Folfirinox**, qui utilise des médicaments démontrés efficaces dans votre maladie, mais pas tous utilisés de façon courante. Ce traitement associe trois médicaments de chimiothérapie :

- l'association 5-fluorouracile (5FU) et acide folinique (LV5FU), traitement reconnu comme efficace dans votre maladie,
- l'irinotecan (Campto®) et l'oxaliplatine (Eloxatine®), médicaments de chimiothérapie commercialisés jusqu'ici que pour le traitement des cancers du gros intestin.

Cette association (Folfirinox) a déjà été administrée chez des patients atteints d'un cancer du pancréas et ce traitement a montré une efficacité encourageante.

L'attribution de l'une de ces options est déterminée par tirage au sort effectué par ordinateur central. Le médecin et le patient n'ont aucune influence sur l'attribution des traitements.

Traitement par Folfirinox :

L'oxaliplatine s'administre par voie intraveineuse sur une durée de deux heures en perfusion le premier jour du traitement. Elle sera suivie de l'administration simultanée de l'acide folinique en perfusion de deux heures et de l'irinotecan en perfusion de 1h30 à débuter immédiatement après la fin de la perfusion d'oxaliplatine.

Le 5-FU s'administre immédiatement après la fin de la perfusion d'acide folinique et consistera en une dose en intraveineuse sur 5 mn puis en une perfusion continue sur 46h.

Ce traitement sera renouvelé 14 jours plus tard et pour une durée maximale de 6 mois soit 12 cycles

Traitement par Gemcitabine :

La gemcitabine s'administre par voie intraveineuse le premier jour de traitement (J1) puis chaque semaine à J8, J15, J22, J29, J36, J43. Une cure dure 56 jours.
Cette administration sera ensuite reprise à J57 pendant 3 semaines sur 4, soit à J57, J64 et J71 et sera suivi d'une semaine de pause. Ce le même schéma (3 semaines sur 4) sera poursuivi pour une durée de 6 mois.

Avant chaque cycle de chimiothérapie un bilan biologique ainsi qu'un examen clinique seront réalisés afin de vérifier que vous tolérez bien le traitement. Tous les quinze jours avant la consultation, votre médecin vous demandera de remplir un questionnaire dit de « qualité de vie » pour savoir comment vous supportez les traitements et comment se passe votre vie au quotidien. Il faut environ dix minutes pour y répondre. Le remplissage de ce questionnaire est très important pour évaluer votre qualité de vie tout au long de votre maladie.

Un bilan à huit semaines vous sera fait. Votre médecin vous fera un examen clinique, un bilan biologique, un scanner et vous demandera de renseigner le questionnaire de qualité de vie.

Un scanner vous sera fait tous les deux mois à partir de l'inclusion. Votre participation à cette étude n'entraînera pas de contraintes supplémentaires.

A tout moment votre médecin pourra décider de suspendre le traitement si ce traitement ne s'avère pas efficace sur votre maladie, si les effets indésirables du traitement sont jugés trop dangereux pour vous, si de nouvelles informations concernant le traitement suggèrent une inefficacité ou un danger. Vous pouvez également choisir de vous retirer de l'essai à tout moment pour quelques raisons que ce soit sans qu'il n'y ait de préjudice pour la prise en charge de votre maladie. Dans les deux cas une thérapeutique vous serait proposée par l'équipe médicale qui vous prend en charge.

Le Folfirinox comme la gemcitabine peuvent être réalisés en ambulatoire

4. Quelle est la durée de participation du patient à l'étude ?

Dans tous les cas, deux mois de traitement par chimiothérapie sont initialement prévus puis, en fonction des résultats d'un bilan réalisé tous les deux mois et de la tolérance de votre traitement, vous serez suivi pendant les six mois, puis régulièrement pendant au moins 2 ans.

5. Quels sont les bénéfices attendus ?

Le but de cette étude est d'améliorer le traitement de votre maladie en évaluant l'efficacité et la tolérance de ces deux chimiothérapies.

Le bénéfice principal attendu est une réduction totale ou partielle du volume de vos lésions et une diminution des symptômes que vous ressentez. Le bénéfice pourrait être également une plus grande efficacité du traitement (plus longue durée sans évolution de la maladie) et/ou une toxicité moins importante.

6. Quels sont les risques possibles ?

Comme tous les médicaments de ce type, la chimiothérapie que vous allez recevoir peut être responsable d'effets secondaires indésirables. Ceux-ci sont connus par les médecins qui vous surveilleront et vous donneront soit des traitements préventifs (anti-nauséeux, anti-vomissements), soit des traitements curatifs. Signalez tout effet auprès de votre médecin.

- Des nausées et des vomissements transitoires seront atténués par la prescription d'anti-émétiques;
- Un risque d'infection par baisse du nombre de globules blancs sanguins, un risque accru d'hémorragies et donc d'apparition spontanée d'écchymoses ou d'hématomes, une anémie par baisse du nombre de globules rouges ainsi qu'une fatigue sont des événements assez fréquents : ils sont surveillés par des prises de sang régulières ;
- L'apparition d'une fièvre (température supérieure à 38°C) peut témoigner d'une infection sévère, notamment si elle est associée à une diarrhée. Une fièvre nécessite d'appeler votre médecin traitant qui prescrira une prise de sang,
- Des douleurs musculaires liées à l'administration de l'oxaliplatine, peuvent être cause de l'arrêt du traitement; en général, cette neurotoxicité disparaît en 6 à 8 mois .
- Avec l'oxaliplatine, une gêne à la perception fine peut gêner la vie quotidienne (bouton de chemise par exemple)et nécessiter une diminution du traitement.
- Une fatigue anormale, est également possible
- Pendant la perfusion d'irinotecan, vous pourrez ressentir une transpiration abondante, des crampes abdominales, une diarrhée, une augmentation de la salivation ou des troubles visuels. Lorsqu'ils apparaissent, ces symptômes sont en général modérés et sont traités efficacement par une injection d'atropine, si vous ne présentez pas de contre-indication à ce médicament. Si cette injection a été nécessaire, elle sera répétée à titre préventif lors des traitements suivants
- Pendant la perfusion d'oxaliplatine, il arrive très rarement une sensation de spasme dans la gorge. C'est une illusion liée à l'anesthésie de la gorge mais il n'y a aucun risque de troubles respiratoires. Cet évènement est bénin et disparaît tout seul. Cependant par la suite, il nécessitera d'allonger la perfusion d'oxaliplatine à 6 heures (au lieu de 2 heures).
- Une diarrhée qui, rarement, peut être sévère. Si c'est le cas, vous devrez prendre un traitement anti-diarrhéique, du Lopéramide, pour le traitement de cette diarrhée. En cas de diarrhée sévère et persistante, votre médecin y ajoutera des antibiotiques. Si la diarrhée persiste, il peut décider d'une éventuelle hospitalisation pour quelques jours. Une note spécifique de recommandations et une ordonnance de Lopéramide et d'antibiotiques vous sera remise à la sortie de l'hôpital. Il vous est demandé de lire attentivement et de suivre rigoureusement ces recommandations
- Une chute de cheveux peut se produire et vous pourrez avoir une prescription de perruque si vous le désirez. Il est également possible d'utiliser un casque réfrigérant, ce qui limite le risque de chute des cheveux.
- Des fourmillements des extrémités en touchant un objet froid pendant quelques jours. Il peut également arriver des crampes des mollets pendant un ou deux jours.
- Une fièvre modérée et un peu de douleurs dans les muscles dans la nuit qui suit la perfusion de gembitabine.
- Lors d'un traitement par Oxaliplatine et 5-FU, des élévations des enzymes du foie lors des examens sanguins de contrôle sont fréquemment constatés. Ces élévations sont généralement peu importantes et n'entraînent aucun symptôme. Dans quelques cas, des symptômes peuvent être associés avec ces élévations des enzymes du foie et consister en une jaunisse, une ascite (accumulation de liquide dans l'abdomen), ou une augmentation de volume du foie et/ou de la rate. Ces anomalies du foie et ces symptômes peuvent être liés à votre maladie ou, dans de rares cas, refléter un effet direct du traitement sur le tissu du foie, qui pourrait modifier les

vaisseaux sanguins dans le foie (maladie veino-occlusive). Pendant la durée de l'étude, la fonction de votre foie sera contrôlée de façon régulière par des examens sanguins et, si un mauvais fonctionnement du foie ou des symptômes sans rapport avec votre maladie venaient à survenir, des examens complémentaires vous seraient proposés.

- En cas d’anémie, votre médecin pourra vous prescrire un traitement par érythropoïétine (Aranesp). Dans la mesure où il a été observé chez certains patients traités par érythropoïétine une augmentation de fréquence d'accidents thromboemboliques notamment lorsque le taux d’hémoglobine est supérieur à 13 g/dl, une surveillance accrue de votre numération sanguine sera effectuée. D'autre part, comme avec tout facteur de croissance, on ne peut pas exclure totalement le risque de croissance tumorale avec les érythropoïétines.

Ces effets secondaires, dont nous tenons à vous donner le descriptif détaillé, sont généralement modérés et ne nécessitent pas, le plus souvent, l’arrêt du traitement. Le traitement sera adapté à votre susceptibilité individuelle. Informer impérativement votre médecin qui jugera avec vous de leur importance. En cas de toxicité importante, les doses des molécules pourront avoir leur posologie diminuée, retardée, voire supprimée.
Vous êtes en droit de demander à tout moment l’arrêt du traitement si vous le souhaitez.

Durant la chimiothérapie, il existe un risque important de malformation en cas de grossesse. Vous ou votre partenaire devez utiliser une contraception efficace pendant la période de chimiothérapie et dans les trois mois qui suivront. Avant de commencer le traitement, votre médecin s’assurera que vous disposez du meilleur moyen de contraception adapté à votre cas.

Il est important que vous informiez votre médecin de tout événement qui pourrait survenir entre deux traitements de chimiothérapie (cures), même si vous pensez que cet événement n’est pas relié au médicament administré. En particulier, vous devez informer votre médecin de toute diarrhée ou fièvre.

7. Quelles sont les alternatives de traitements ?

Ni la chirurgie ni la radiothérapie ne peuvent vous être proposés, sauf dans des cas très particuliers. Les rayons peuvent parfois aider à mieux contrôler les douleurs et la chirurgie est parfois nécessaire pour dériver les voies biliaires et le tube digestif dans certains cas. Le traitement habituel de votre maladie dans votre situation est une chimiothérapie en perfusion. L’alternative est le traitement uniquement de vos symptômes, sans y associer de chimiothérapie.

8. Quels sont vos droits en tant que participant à cette étude ?

Le promoteur de cet essai, la Fédération Nationale des Centres de Lutte Contre le Cancer (FNCLCC), a pris toutes les dispositions prévues par la loi pour la protection des personnes se prêtant à la recherche biomédicale (loi Huriet du 20/12/1988, modifiée). Le promoteur a également souscrit une assurance de recherche biomédicale, conformément à la législation en vigueur, auprès de la Société Gerling France (111-113 rue de Longchamp, 75016 Paris) par l’intermédiaire de la société de courtage d’assurances Biomedic Insure (73, rue du général Weygand, 56037 Vannes, tel. 02 97 69 19 19).
Les modalités de ce protocole ont été soumis à l’examen du Comité Consultatif de Protection des Personnes dans la recherche Biomédicale(CCPPRB) de Lorraine le 08 avril 2004 lequel a pour mission de vérifier si les conditions requises pour votre protection et le respect de vos droits ont été respectés. Le CCPPRB de Lorraine a rendu un avis favorable le 18 mai 2004
De plus selon les dispositions de la loi du 4 mars 2002, vous serez informé des résultats globaux de l’essai par l’investigateur.
Ce protocole a été examiné par le Comité de Patients de la Ligue Nationale Contre le Cancer.

Votre dossier médical restera naturellement confidentiel et ne pourra être consulté que sous la responsabilité du médecin s’occupant de votre traitement ainsi que par les autorités de santé et par les personnes dûment mandatées par l’organisateur de la recherche soumises au secret professionnel.

9. A qui devez-vous vous adresser en cas de questions ou de problèmes ?

En cas de problèmes ou de questions, vous pouvez, vous adresser aux personnes suivantes :

Vos contacts dans l'étude	Coordonnées du médecin traitant du patient
(titre, nom, prénom, adresse et téléphone) :<br>………………………………………………<br>………………………………………………<br>………………………………………………<br>………………………………………………<br>………………………………………………	………………………………………………<br>………………………………………………<br>………………………………………………<br>………………………………………………<br>………………………………………………

(1) toutes les pages doivent être paraphées par l'investigateur/co-investigateur et le patient

10. Quel est le calendrier de votre traitement et de vos examens ?

Plan de traitement													
**Date**	**J1**	**J8**	**J15**	**J22**	**J29**	**J36**	**J43**	**J50**	**J57**	**J64**	**J71**	**J78**	**Jn**
**Bras A : FOLFIRINOX**	**C1**		**C2**		**C3**		**C4**		**C5**		**C6**		**Cn**
	oxaliplatine 85 mg/m^2 J1+ irinotecan 180 mg/m^2 J1+ acide folinique 400 mg/m^2 J1, 5-FU bolus 400 mg/m² J1, 5-FU continu 2,4 g/m^2 sur 46 heures J1/ J2 de chaque cycle								*Reprise du traitement selon le schéma précédent, toutes les 2 semaines*				
**Bras B : gemcitabine**	↑	↑	↑	↑	↑	↑	↑		↑	↑	↑		↑
	gemcitabine 1000 mg/m² de chaque semaine pendant 7 semaines								*Reprise de la gemcitabine 3 semaines sur 4*				

12 cycles prévus pour le bras A = 6 mois de traitement

Plan de surveillance BRAS A Folfirinox									
**Visite**	**Bilan inclusion**	**Bilans de suivi**				**Bilan à 8 semaines**	**Bilans de suivi après reprise**		
**N° de visite**	**V0**	**V1**	**V2**	**V3**	**V4**	**V5**	**V6**	**V7**	**Vn**
**Date**	**J0**	**J1**	**J15**	**J29**	**J43**	**Autour J50**	**J57**	**J71**	**Jn**
Consentement éclairé signé	**X**								
Critères d'inclusion / non inclusion	**X**								
Randomisation	**X**								
**Examen clinique**									
Poids	**X**		**X**	**X**	**X**	**X**	**X**	**X**	**X**
Taille Surface.corporelle	**X**								
Etat général / OMS	**X**		**X**	**X**	**X**	**X**	**X**	**X**	**X**
Traitements concomitants	**X**								
**Toxicités (2)**									
Evaluation de la tolérance			**X**	**X**	**X**	**X**	**X**	**X**	**X**
**Examen biologique** (4)									
NFS, plaquettes	**X**	**X**	**X**	**X**	**X**	**X**	**X**	**X**	**X**
TP,TCK	**X**								
Ionogramme, Ca	**X**		**X**	**X**	**X**	**X**	**X**	**X**	**X**
Protidémie	**X**		**X**	**X**	**X**	**X**	**X**	**X**	**X**
Albuminémie, LDH	**X**								
Bilan hépatique	**X**		**X****	**X****	**X****	**X**	**Examen tous les deux mois**		
Creatininémie	**X**	**X**	**X**	**X**	**X**	**X**	**Examen tous les deux mois**		
Glycémie	**X**		**X**	**X**	**X**	**X**	**X**	**X**	**X**
ACE, Ca 19-9 *	**X**					**X**	**Examen tous les deux mois**		
Test de grossesse	**X**(1)								
**Bilan paraclinique**(3)									
Scanner Thoracoabdominopelvien	**X**					**X**	**Examen scanographique tous les 2 mois**		
ECG	**X**								
**Questionnaire de Qualité de Vie**	**X**		**X**	**X**	**X**	**X**	**X**	**X**	**X**

6 mois de traitement prévus pour le bras B

**Plan de surveillance BRAS B GEMCITABINE**													
**Visite**	**Bilan inclusion**	**Bilans de suivi**							**Bilan à 8 semaines**	**Bilans de suivi après reprise**			
**N° de visite**	**V0**	**V1**	**V2**	**V3**	**V4**	**V5**	**V6**	**V7**	**V8**	**V9**	**V10**	**V11**	**Vn**
**Date**	**J0**	**J1**	**J8**	**J15**	**J22**	**J29**	**J36**	**J43**	**Autour J50**	**J57**	**J64**	**J71**	**Jn**
Consentement éclairé signé	**X**												
Critères d'inclusion / non inclusion	**X**												
Randomisation	**X**												
**Examen clinique**	**X**												
Poids	**X**	**X**		**X**		**X**		**X**	**X**	**X**		**X**	
Taille Surface.corporelle	**X**												
Etat général / OMS	**X**	**X**		**X**		**X**		**X**	**X**	**X**		**X**	
Traitements concomitant	**X**												
**Toxicités (2)**													
Evaluation de la tolérance			**X**	**X**	**X**	**X**	**X**	**X**	**X**	**X**	**X**	**X**	**X**
**Examen biologique** (4)													
NFS, plaquettes	**X**	**X**	**X**	**X**	**X**	**X**	**X**	**X**	**X**	**X**	**X**	**X**	**X**
TP,TCK	**X**												
Ionogramme, Ca	**X**			**X**		**X**		**X**	**X**	**X**		**X**	
Protidémie	**X**								**X**				
Albuminémie, LDH	**X**												
Bilan hépatique	**X**			**X**		**X**		**X**	**X**	**Examen tous les deux mois**			
Créatininémie	**X**			**X**		**X**		**X**	**X**	**Examen tous les deux mois**			
Glycémie	**X**			**X**		**X**		**X**	**X**	**X**		**X**	
ACE, Ca 19-9*	**X**								**X**	**Examen tous les deux mois**			
Test de grossesse	**X**(1)												
**Bilan paraclinique**(3)													
Scanner Thoracoabdomino pelvien	**X**								**X**	**Examen scanographique tous les 2 mois**			
ECG	**X**												
**Questionnaire de Qualité de Vie**	**X**			**X**		**X**		**X**	**X**	**X**		**X**	

**seul le marqueur le plus performant sera dosé ultérieurement, choisir le marqueur qui a la valeur initiale la plus importante par rapport à la normale*

*** Bilan hépatique avec Bilirubine totale, libre et conjuguée doit être fait au moins tous les mois*

(1) pour les femmes en âge de procréer sans moyen contraceptif efficace

(2) Evénements Indésirables Graves (EIG) à déclarer dans les 48 heures au promoteur jusqu'à 30 jours après la fin de la dernière cure de chimiothérapie. Au delà de cette période seuls les EIG susceptibles d'être dus à la recherche seront déclarés au promoteur dès lors qu'aucune autre cause que la recherche ne peut raisonnablement être attribuée.

(3) Le bilan radiologique peut être effectué au cours des 3 semaines précédant l'inclusion.

(4) Le bilan biologique doit être effectué au plus tard dans la semaine précédant l'inclusion.

ANNEXE 7 :
Formulaire de consentement de participation du patient(1)

ETUDE RANDOMISEE DE PHASE II COMPARANT L'ASSOCIATION [OXALIPLATINE / IRINOTECAN / LV5FU (FOLFIRINOX)] A LA GEMCITABINE SEULE EN PREMIERE LIGNE DE TRAITEMENT DE PATIENTS ATTEINTS D'UN CANCER DU PANCREAS METASTATIQUE

Je soussigné(e) :

Nom :……………………………………….Prénom :……………………………………………..

Adresse :……………………………………………………………………………………………..

CONSENS EXPRESSEMENT A PARTICIPER A CETTE RECHERCHE DANS LES CONDITIONS QUI M'ONT ETE PRECISEES DANS LA FICHE D'INFORMATION.

J'ai reçu et j'ai bien compris les informations qui m'ont été remises par le Dr ……………….. qui m'a expliqué l'objectif, le déroulement, et la durée de cette recherche, ainsi que les bénéfices attendus et les risques éventuels, et qui m'a précisé que je suis libre d'accepter ou de refuser.

Mon consentement ne décharge par les organisateurs de la recherche de leurs responsabilités. Je conserve tous mes droits garantis par la loi.

Si je le désire, je serai libre à tout moment d'arrêter ma participation. J'en informerai alors le Dr ……………….. Il me proposera, si je le souhaite un autre traitement.

Dans le cadre d'éventuelles publications scientifiques, seules les informations ne faisant mention, ni de mon nom, ni de mon adresse, peuvent être utilisées. Les données qui me concernent resteront strictement confidentielles et je n'autorise leur consultation que par des personnes mandatées par l'organisateur de la recherche ou par un représentant des Autorités de Santé.

J'accepte également que les données enregistrées à l'occasion de cet essai puissent faire l'objet d'un traitement informatisé par le promoteur ou pour son compte conformément à la loi Informatique et Liberté du 6/1/1978 modifiée par les lois n°94-548 du 1/7/1994 et n° 2002-303 du 4/3/2002. J'ai bien noté que le droit d'accès (articles 34 et 40 modifié) et de rectification (article 36), que m'ouvrent les textes susvisés, pourra s'exercer à tout moment auprès du Dr…………………………………………………et que les données me concernant pourront m'être communiquées ou par l'intermédiaire d'un médecin de mon choix.

Nom du patient ou de son représentant légal	Date	Signature
Nom du médecin investigateur ou du médecin qui le représente (co-investigateur) :	Date	Signature

(1) (toutes les pages doivent être paraphées, un exemplaire cosigné doit être remis à la personne qui participe à la recherche)

ANNEXE 8 :
Conseils pour l'utilisation du questionnaire EORTC QLQ-30 pour mesurer la qualité de vie

1 – PASSATION DU QUESTIONNAIRE

- Fournir un environnement calme et confortable pour le remplissage du questionnaire.
- Remettre le questionnaire au malade en personne en pensant à emporter un stylo bille supplémentaire (le patient peut ne pas avoir de stylo avec lui).
- Prévoir une aide par une personne désignée (par exemple infirmière, assistante, technicienne de recherche clinique, secrétaire) au cas où le malade ait besoin d'explications.
- Expliquer verbalement les instructions ou les questions si celles-ci ne paraissent pas claires sans influencer les réponses. Lui montrer comment entourer (et non cocher) les réponses.
- Parfois remplir à la place du patient en lui posant les questions (patients âgés, fatigués ou ayant oublié ses lunettes …) et alors le mentionner.
- Vérifier lors de la récupération du questionnaire que les données non remplies le sont délibérément et ne résultent pas d'un oubli.
- Noter la cause du refus si le patient refuse de remplir l'ensemble du questionnaire.

2 – MOMENT DE REMISE DU QUESTIONNAIRE

a – Avant l'inclusion

- Le patient doit être au courant de sa situation avant le remplissage du questionnaire de qualité de vie.
- Le médecin doit avoir expliqué le but du questionnaire.
- Faire remplir le questionnaire avant randomisation pour que l'issue de la randomisation n'influe pas les résultats de qualité de vie.
- Le remplissage du questionnaire sera un critère d'inclusion, car disposer du questionnaire avant traitement permettra de détecter un biais de sélection si le suivi ultérieur vient à manquer (patients avec une qualité de vie médiocre à l'inclusion auxquels on n'ose plus ensuite présenter un questionnaire car la QdV s'est encore dégradée).

b – Avant chaque cure

- Le questionnaire est considéré comme acceptable s'il est rempli le jour du traitement ou dans les 3 jours qui précèdent celui-ci. Le remplissage par téléphone est déconseillé.
- Si le traitement est reporté, le remplissage du questionnaire le sera également.

c – A chaque remise de questionnaire

- Le questionnaire est remis au malade et rempli préférentiellement avant que celui-ci ait vu le médecin, à la fois pour réduire les biais liés au dialogue avec le médecin ou ceux dus aux résultats du traitement et aussi pour permettre au patient de discuter avec le médecin des symptômes ou des domaines de qualité de vie réduite.
- Déconseiller d'emmener le questionnaire à la maison car il n'y aura aucun contrôle sur le jour où le questionnaire sera rempli réellement et les réponses au questionnaire seront influencées par la famille ou les amis.
- En cas de symptômes sévères décrits sur le questionnaire de qualité de vie, la personne qui récupère le questionnaire peut rappeler au patient qu'il doit signaler les problèmes au médecin responsable de sa chimiothérapie.
- Il faut remettre le questionnaire à chaque cure prévue et aussi à la sortie d'étude, même si le patient ne va pas bien (c'est précisément à ce moment-là qu'il est intéressant d'étudier les altérations de la qualité de vie et qu'il est important de tenter de les corriger).

3 – ORGANISATION INTERNE DE CHAQUE CENTRE

- Nommer dans chaque centre un responsable de la remise des questionnaires et prévoir son remplacement en cas d'absence ou de vacances.
- Prévoir un échéancier de remise des questionnaires en même temps que la randomisation dans l'étude, en remettre un double au patient et le lui expliquer.
- Chaque centre gardera une copie des questionnaires avant de les envoyer avec les fiches de l'étude.
- Mettre un double du résumé du protocole dans chaque dossier et rendre accessible le protocole complet dans chaque secteur de soins.

ANNEXE 9 :
EORTC QLQ-C30 (version 3.0)

Nous nous intéressons à vous et à votre santé. Répondez vous-même à toutes les questions ***en entourant le chiffre*** qui correspond le mieux à votre situation. Il n'y a pas de "bonne" ou de "mauvaise" réponse. Ces informations sont strictement confidentielles.

	**Pas du tout**	**Un peu**	**Assez**	**Beaucoup**
1. Avez-vous des difficultés à faire certains efforts physiques pénibles, comme porter un sac à provision chargé ou une valise ?	1	2	3	4
2. Avez-vous des difficultés à faire une longue promenade ?	1	2	3	4
3. Avez-vous des difficultés à faire un petit tour dehors ?	1	2	3	4
4. Etes-vous obligé(e) de rester au lit ou dans un fauteuil pendant la journée ?	1	2	3	4
5. Avez-vous besoin d'aide pour manger, vous habiller, faire votre toilette ou aller aux W.C ?	1	2	3	4

AU COURS DE LA SEMAINE PASSEE :

	**Pas du tout**	**Un peu**	**Assez**	**Beaucoup**
6. Avez-vous été gêné(e) pour faire votre travail ou vos activités de tous les jours ?	1	2	3	4
7. Avez-vous été gêné(e) dans vos activités de loisirs ?	1	2	3	4
8. Avez-vous eu le souffle court ?	1	2	3	4
9. Avez-vous eu mal ?	1	2	3	4
10. Avez-vous eu besoin de repos ?	1	2	3	4
11. Avez-vous eu des difficultés pour dormir ?	1	2	3	4
12. Vous êtes-vous senti(e) faible ?	1	2	3	4
13. Avez-vous manqué d'appétit ?	1	2	3	4
14. Avez-vous eu des nausées (mal au cœur) ?	1	2	3	4
15. Avez-vous vomi ?	1	2	3	4
16. Avez-vous été constipé(e) ?	1	2	3	4
17. Avez-vous eu de la diarrhée ?	1	2	3	4
18. Etiez-vous fatigué(e) ?	1	2	3	4

AU COURS DE LA SEMAINE PASSEE :

	**Pas du tout**	**Un peu**	**Assez**	**Beaucoup**
19. Des douleurs ont-elles perturbé vos activités quotidiennes ?	1	2	3	4
20. Avez-vous eu des difficultés à vous concentrer sur certaines choses, par exemple, pour lire le journal ou regarder la télévision ?	1	2	3	4
21. Vous êtes-vous senti(e) tendu(e) ?	1	2	3	4
22. Vous êtes-vous fait du souci ?	1	2	3	4
23. Vous êtes-vous senti(e) irritable ?	1	2	3	4
24. Vous êtes-vous senti(e) déprimé(e) ?	1	2	3	4
25. Avez-vous eu des difficultés pour vous souvenir de certaines choses ?	1	2	3	4
26. Votre état physique ou votre traitement médical vous ont-ils gêné(e) dans votre vie familiale ?	1	2	3	4
27. Votre état physique ou votre traitement médical vous ont-ils gêné(e) dans vos activités sociales (par exemple sortir avec des amis, aller au cinéma ...) ?	1	2	3	4
28. Votre état physique ou votre traitement médical vous ont-ils causé des problèmes financiers ?	1	2	3	4

Pour les deux questions suivantes, veuillez répondre en entourant le chiffre entre 1 et 7 qui s'applique le mieux à votre situation

29. Comment évalueriez-vous votre état de santé au cours de la semaine passée ?

1	2	3	4	5	6	7
**Très mauvais**						**Excellent**

30.Comment évalueriez-vous l'ensemble de votre qualité de vie au cours de la semaine passée ?

1	2	3	4	5	6	7
**Très mauvais**						**Excellent**

ANNEXE 10 :
recommandations pour l'utilisation du filgrastim : (NEUPOGEN®)

ADMINISTRATION / POSOLOGIE :

La dose recommandée de NEUPOGEN est de 0,5 MU (5 µg)/kg/jour. La première injection de NEUPOGEN doit être effectuée au plus tôt 24 heures après la fin de la chimiothérapie cytotoxique. NEUPOGEN doit être administré de façon quotidienne par voie sous-cutanée ou en perfusion intraveineuse de 30 minutes, la solution de NEUPOGEN étant alors diluée dans une solution de glucose à 5% (pour une information complète concernant la dilution, consulter le dictionnaire VIDAL).
La préférence doit être donnée à la voie sous-cutanée dans la majorité des cas.
L'administration quotidienne de NEUPOGEN doit être poursuivi jusqu'à ce que le nadir du nombre de neutrophiles soit dépassé et que ce nombre soit revenu à une valeur normale.

MISES EN GARDE SPECIALES :

Chez les patients cancéreux : Leucocytose : Réaliser une numération leucocytaire à intervales réguliers. Arrêt du traitement si le nombre de leucocytes dépasse $50x10^9$/l après la date attendue du nadiraprès une chimiothérapie ou arrêt ou diminution de la posologie si le taux de leucocytes dépasse $70x10^{9/l}$ lors d'une collection de cellules souches.
Risques associés à la chimiothérapie intensive : Pas d'action sur la thrombopénie, ni sur l'anémie dues à la chimiothérapie cytotoxique. Surveiller le nombre de plaquettes et l'hématocrite.
Autres précautions : Prudence en cas d'intolérance héréditaire au fructose.

INTERACTIONS *:

Administration non recommandée dans les 24 heures précédent ou suivant une chimiothérapie myélosuppressive.

CONTRE-INDICATIONS :

NEUPOGEN ne doit pas être administré aux patients présentant une hypersensibilité connue au produit ou à l'un de ses constituants. NEUPOGEN ne doit pas être administré pour augmenter les doses de chimiothérapie cytotoxique au-delà des dosages établis. NEUPOGEN ne doit pas être administré à des patients présentant une neutropénie congénitale sévère (syndrome de Kostmann) avec anomalies cytogénétiques

GROSSESSE ET ALLAITEMENT *:

Innocuité non établie.

EFFET INDESIRABLE *:

Chez les patients atteint de cancer : Le plus fréquent : douleurs osseuses, troubles urimaires, modifications biologiques légères ou modérées, dose-dépendantes et réversibles à l'arrêt du traitement (élévation de gamma GT, élévation des Phosphatases Alcalines, élévation du taux de LDH).
Dans de rares cas : Baisses transitoires de la pression artérielles, perturbations vasculaires, vascularités cutanées, syndrome de Sweet (dermatose fébrile aiguë), poussées de polyarthrite rhumatoïde, syndrome de détresse respiratoire de l'adulte et réaction allergique.

INCOMPATIBILITES *: Solutions salines

* **pour une information complète, consulter le dictionnaire VIDAL**

<u>ANNEXE 11 :</u>
<u>recommandations pour l'utilisation de la darbepoetin alfa (ARANESP®)</u>

ADMINISTRATION / POSOLOGIE :

L'Aranesp®sera administré en sous cutané à une dose initiale de 6,75 µg/kg, une fois toutes les 3 semaine, chez les patients présentant une anémie (par ex. taux d'hémoglobine ≤ 11 g/dl (6,8 mmol/l)). Le taux cible d'hémoglobine à atteindre pour corriger l'anémie et réduire les besoins transfusionnels est de 12,0 g/dl ou 7,5 mmol/l.

- Si le taux d'hémoglobine dépasse 14,0 g/dl ou 8,7 mmol/l, le traitement doit être interrompu *(voir infra, § 2)*.
- Si la réponse clinique (fatigue, taux d'hémoglobine) n'est pas satisfaisante après 9 semaines (3 injections), arrêt de l'Aranesp® .

Les indications transfusionnelles sont laissées au libre choix de clinicien.

Ne pas oublier de faire une supplémentation en fer si le taux de ferritine sérique est < 100 µg/l ou si le coefficient de saturation de la transferrine est <20 %)

Le traitement par Aranesp® doit être poursuivi au moins pendant 4 semaines après l'arrêt de la chimiothérapie, ou jusqu'à ce que le taux d'hémoglobine se soit normalisé à une valeur supérieure ou égale à 12,0 g/dl ou 7,5 mmol/l. Une mesure de la NFS sera effectuée de façon hebdomadaire.

CONDUITE A TENIR :

1) **En cas d'événement indésirable grave** : A n'importe quel moment de l'étude, tout événement indésirable grave qui serait considéré par l'investigateur comme lié au traitement par Aranesp® entraînera l'arrêt immédiat du traitement. Suite à cela, un suivi régulier du patient sera assuré jusqu'à la disparition ou la stabilisation de cet événement.

Il est recommandé de ne pas administrer l'Aranesp® si le taux d'hémoglobine est > à 11 g/dL. Il est nécessaire de veiller à ce que ce taux ne dépasse pas 13g/dL. En cas d'augmentation de taux d'hémoglobine de plus de 2 g/dL en quatre semaines, réduire la posologie de 25 à 50 %.

ANNEXE 12 :
Liste des EIG attendus

Extrait du RCP oxaliplatine - ELOXATINE®

DC/EFFETS INDÉSIRABLES
Les effets indésirables rapportés lors du développement clinique de l'oxaliplatine dans le traitement du cancer colorectal métastatique ont été analysés sur une population de 244 patients traités en monothérapie et près de 1500 patients traités en association avec le 5-fluorouracile.

- **Système hématopoïétique :**

L'oxaliplatine administré en monothérapie (130 mg/m ^{2} toutes les 3 semaines) entraîne peu de toxicité hématologique de grades 3 et 4.

Oxaliplatine seul	Tous grades	Grade 3	Grade 4
Anémie (% patients)	64	3	< 1
Neutropénie (% patients)	15	2	< 1
Thrombopénie (% patients)	41	2	< 1

Lorsque l'oxaliplatine est utilisé en association avec le 5-fluorouracile et l'acide folinique, l'incidence des neutropénies et des thrombopénies est supérieure à celle observée avec l'association 5-fluorouracile/acide folinique seule.

Oxaliplatine associé au 5-fluorouracile	85 mg/m ^{2} toutes les 2 sem		
	Tous grades	Grade 3	Grade 4
Anémie (% patients)	83	4	< 1
Neutropénie (% patients)	66	25	13
Thrombopénie (% patients)	76	3	< 1

- Système digestif :

En monothérapie, l'oxaliplatine (130 mg/m ^{2} toutes les 3 semaines) peut entraîner une anorexie, des nausées, des vomissements, des diarrhées et des douleurs abdominales, non sévères dans la majorité des cas.

Oxaliplatine seul	Tous grades	Grade 3	Grade 4
Nausées, vomissements (% patients)	69	12	2
Diarrhées (% patients)	41	4	< 1
Mucites (% patients)	4	< 1	< 1
Anomalies hépatiques (% patients)	46	10	2

Un traitement préventif et/ou curatif avec des agents antiémétiques puissants est indiqué. Lorsque l'oxaliplatine est associé avec le 5-fluorouracile (avec ou sans acide folinique), la fréquence, comme la sévérité, des diarrhées et des mucites est augmentée de façon significative comparativement à celle observée avec le 5-fluorouracile seul.
De rares cas de colites, incluant des diarrhées à Clostridium difficile ont été rapportés. Une déshydratation, un iléus paralytique, une occlusion intestinale, une hypokaliémie, une acidose métabolique et une altération de la fonction rénale peuvent être provoqués par des diarrhées et/ou des vomissements sévères, notamment lorsque l'oxaliplatine est associé au 5-fluorouracile (cf Mises en garde/Précautions d'emploi).

Oxaliplatine associé au 5-fluorouracile	85 mg/m ^{2} toutes les 2 sem		
	Tous grades	Grade 3	Grade 4
Nausées, vomissements (% patients)	71	11	1
Diarrhées (% patients)	58	7	3
Mucites (% patients)	42	7	1

Des élévations des enzymes hépatiques de grades 1 et 2 sont fréquentes lors du traitement par oxaliplatine. Dans les études randomisées comparant l'association 5-fluorouracile/acide folinique à l'association 5-fluorouracile/acide folinique/oxaliplatine, l'incidence des élévations des enzymes hépatiques de grades 3 et 4 est comparable dans les deux groupes.

- Système nerveux :

La toxicité limitante de l'oxaliplatine est neurologique. Il s'agit essentiellement d'une neuropathie périphérique sensitive caractérisée par des dysesthésies et/ou des paresthésies des extrémités, accompagnées ou non de crampes, souvent déclenchées par le froid. Ces symptômes apparaissent chez 85 à 95 % des patients traités. La durée de ces symptômes, généralement régressifs entre les cycles de traitement, s'accroît avec la répétition de ceux-ci.

La survenue de douleurs et/ou d'une gêne fonctionnelle nécessite, selon la durée des symptômes, l'ajustement de la dose, voire l'arrêt du traitement (cf Mises en garde/Précautions d'emploi).
Cette gêne fonctionnelle, qui comprend des difficultés lors de l'exécution des gestes fins, est une conséquence possible de l'atteinte sensitive. Le risque de survenue d'une gêne fonctionnelle pour une dose cumulée d'environ 800 mg/m ^{2} (soit 10 cycles) est de 15 % ou moins. La symptomatologie neurologique s'améliore le plus souvent à l'arrêt du traitement.
Des manifestations neurosensorielles aiguës ont été rapportées (cf Sécurité préclinique). Elles débutent dans les heures suivant l'administration et surviennent souvent lors d'une exposition au froid. Elles se caractérisent par des paresthésies transitoires, des dysesthésies ou hypoesthésies, voire par un syndrome aigu de dysesthésie pharyngolaryngée. Ce syndrome aigu, dont l'incidence est estimée entre 1 % et 2 %, se caractérise par des sensations subjectives de dysphagie ou de dyspnée sans signe objectif de détresse respiratoire (sans cyanose, ni hypoxie) ou par laryngospasme ou bronchospasmes (sans stridor ou sifflement) ; une contracture de la mâchoire, une dysesthésie linguale, une dysarthrie et une oppression thoracique ont également été observées.
Bien que des antihistaminiques et des bronchodilatateurs aient été administrés dans ces situations, cette symptomatologie est rapidement réversible, même en l'absence de tout traitement. L'allongement de la durée de la perfusion dans les cycles suivants favorise la diminution de l'incidence de ce syndrome (cf Mises en garde/Précautions d'emploi).
D'autres symptômes neurologiques, comme une dysarthrie, la disparition des réflexes ostéotendineux et un signe de Lhermitte, ont été rapportés lors de traitement par oxaliplatine. Des cas isolés de névrites optiques ont été rapportés.

- Réactions allergiques :

Des réactions anaphylactiques peu fréquentes (en monothérapie) ou fréquentes (en association avec le 5-fluorouracile ± acide folinique) ont été rapportées incluant des cas de bronchospasme, d'angiœdème, d'hypotension et de choc anaphylactique.

Des cas fréquents de réactions allergiques tels que rash cutané (en particulier urticaire), conjonctivite, rhinite ont été rapportés.

- Autres effets :

Une ototoxicité clinique est survenue chez moins de 1 % des patients traités par oxaliplatine. De rares cas de surdité ont été rapportés.
Des anomalies de la fonction rénale ont été rapportées chez environ 3 % des patients traités, des anomalies de grades 3 et 4 chez moins de 1 % des patients.
Lors des études cliniques, ainsi que depuis sa mise sur le marché, aucune arythmie ventriculaire significative n'a été rapportée lors d'administration d'oxaliplatine.
Des cas très fréquents de fièvre ont été rapportés : soit des fièvres isolées de type immunologique soit des fièvres d'origine infectieuse (associées ou non à une neutropénie).
De rares cas de thrombocytopénies immunoallergiques et d'anémies hémolytiques immunoallergiques ont été rapportées.
De rares cas de pneumopathies interstitielles aiguës et de fibroses pulmonaires ont été rapportés (cf Mises en garde/Précautions d'emploi).
Une alopécie modérée a été rapportée chez 2 % des patients traités par l'oxaliplatine seul ; l'association de l'oxaliplatine et du 5-fluorouracile n'augmente pas l'incidence des alopécies observées lors des traitements par le 5-fluorouracile seul.
L'extravasation peut provoquer une douleur locale ainsi qu'une inflammation, pouvant être sévère et entraîner des complications, particulièrement lorsque l'oxaliplatine est perfusé par voie veineuse périphérique (cf Mises en garde/Précautions d'emploi).
Une baisse transitoire de l'acuité visuelle a été rapportée chez moins de 0,1 % des patients à la suite d'une administration d'oxaliplatine.
Des dysarthries ont été rapportées rarement (cf supra : système nerveux).

Extrait du RCP irinotécan- CAMPTO®

DC/EFFETS INDÉSIRABLES

Les effets indésirables suivants, possiblement ou probablement liés à l'administration de Campto, ont été analysés sur une population de 765 patients à la dose recommandée de 350 mg/m ^{2} en monothérapie, et de 145 patients traités par Campto en association avec 5-FU/AF toutes les 2 semaines à la dose recommandée de 180 mg/m ^{2}.

Effets gastro-intestinaux :

- Diarrhée tardive :

  La diarrhée tardive (survenant plus de 24 heures après l'administration de Campto) constitue une toxicité dose-limitante de Campto.

  - En monothérapie : La diarrhée sévère est observée chez 20 % des patients qui ont suivi les recommandations de prise en charge de la diarrhée. Une diarrhée sévère est retrouvée dans 14 % des cycles évaluables.Le délai médian d'apparition de la première selle liquide est de 5 jours après la perfusion de Campto.
  - En association : Une diarrhée sévère est observée chez 13,1 % des patients qui ont suivi les recommandations de prise en charge de la diarrhée. Une diarrhée sévère est retrouvée dans 3,9 % des cycles évaluables.

  De rares cas de colite pseudomembraneuse ont été rapportés, dont un cas avec une documentation bactériologique (Clostridium difficile).

- Nausées et vomissements :

  - En monothérapie : Des nausées et vomissements sévères sont observés chez environ 10 % des patients ayant reçu un traitement antiémétique.
  - En association : Une incidence moindre des nausées et vomissements sévères est observée (respectivement 2,1 % et 2,8 % des patients).

- Déshydratation : des épisodes de déshydratation généralement associés à une diarrhée et/ou des vomissements sont rapportés. De rares cas d'insuffisance rénale, d'hypotension ou de collapsus

cardiovasculaire ont été observés chez des patients ayant présenté des épisodes de déshydratation associés à une diarrhée et/ou à des vomissements.

- Autres événements gastro-intestinaux : Une constipation relative à Campto et/ou au lopéramide a été observée en monothérapie chez moins de 10 % des patients et, en association, chez 3,4 % des patients traités. De rares cas d'occlusion intestinale, d'iléus ou d'hémorragie gastro-intestinale, et de rares cas de colites, incluant des typhlites, des colites ischémiques et ulcéreuses ont été rapportés. De rares cas de perforations intestinales sont rapportés. D'autres effets peu sévères incluant anorexie, douleurs abdominales et stomatite ont été également observés.

Hématologie :

La neutropénie constitue une toxicité dose-limitante. La neutropénie était réversible et non cumulative ; le délai médian d'apparition du nadir était de 8 jours, que ce soit en monothérapie ou en association.

- En monothérapie :La neutropénie est observée chez 78,7 % des patients et est sévère (nombre de neutrophiles < 500/mm ^{3}) dans 22,6 % des cas. Parmi les cycles évaluables, 18 % se compliquent d'une neutropénie < 1000/mm ^{3}, dont 7,6 % d'une neutropénie < 500/mm ^{3}.La récupération totale est en général atteinte le 22 ^{e} jour. Une fièvre accompagnée d'une neutropénie sévère est rapportée chez 6,2 % des patients et dans 1,7 % des cycles. Des épisodes infectieux sont survenus chez environ 10,3 % des patients (2,5 % des cycles), ont été associés à une neutropénie sévère chez environ 5,3 % des patients (1,1 % des cycles), et ont entraîné 2 décès. Une anémie a été rapportée chez environ 58,7 % des patients (8 % avec une hémoglobine < 8 g/dl et 0,9 % avec une hémoglobine < 6,5 g/dl).Une thrombocytopénie (< 100 000/mm ^{3}) a été observée chez 7,4 % des patients et 1,8 % des cycles, dont 0,9 % des patients avec des plaquettes ≤50 000/mm ^{3}, soit 0,2 % des cycles. Presque tous les patients ont normalisé leur numération plaquettaire le 22 ^{e} jour.

- En association :Une neutropénie est observée chez 82,5 % des patients et est sévère (nombre de neutrophiles < 500/mm ^{3}) dans 9,8 % des cas. Parmi les cycles évaluables, 67,3 % se compliquent d'une neutropénie < 1000/mm ^{3}, dont 2,7 % d'une neutropénie < 500/mm ^{3}.La récupération totale est en général atteinte dans les 7 à 8 jours. Une fièvre accompagnée d'une neutropénie sévère est rapportée chez 3,4 % des patients et dans 0,9 % des cycles. Des épisodes infectieux sont survenus chez environ 2 % des patients (0,5 % des cycles), ont été associés à une neutropénie sévère chez environ 2,1 % des patients (0,5 % des cycles), et ont entraîné 1 décès. Une anémie est rapportée chez environ 97,2 % des patients (2,1 % avec une hémoglobine < 8 g/dl).Une thrombocytopénie (< 100 000/mm ^{3}) a été observée chez 32,6 % des patients et 21,8 % des cycles. Aucune thrombocytopénie sévère (< 50 000/mm ^{3}) n'a été observée.

Un cas de thrombocytopénie périphérique avec anticorps antiplaquettes est rapporté dans l'expérience clinique obtenue après la mise sur le marché.

Infection :

De rares cas d'insuffisance rénale, d'hypotension ou de collapsus cardiovasculaire ont été observés chez des patients atteints d'infection systémique grave.

Effets indésirables généraux et réactions au site d'injection :

- Syndrome cholinergique aigu : Un syndrome de type cholinergique aigu, transitoire et sévère, est observé chez 9 % des patients traités par monothérapie et chez 1,4 % des patients traités par association. Les principaux symptômes sont définis par une diarrhée précoce et un ensemble de symptômes tels que crampes abdominales, conjonctivites, rhinites, hypotension, vasodilatation, hypersudation, refroidissement, malaises, vertiges, troubles visuels, myosis, larmoiements et hypersalivation survenant pendant ou dans les 24 heures suivant l'administration de Campto. Les symptômes cèdent à l'administration d'atropine (cf Mises en garde/Précautions d'emploi).L'asthénie sévère a été observée chez moins de 10 % des patients traités par monothérapie et chez 6,2 % des patients traités par association. L'imputabilité à Campto n'est pas clairement établie. Une fièvre isolée non accompagnée d'infection ou de neutropénie sévère est survenue chez 12 % des patients traités par monothérapie et chez 6,2 % des patients traités par association. Des réactions modérées au site d'injection ont été peu fréquemment rapportées.

- Troubles cardiovasculaires : de rares cas d'hypertension pendant ou suivant la perfusion ont été rapportés.

- Troubles respiratoires : de rares cas de pneumopathie interstitielle et de pneumonie avec infiltrats pulmonaires ont été observés. Des effets précoces tels que dyspnée ont été rapportés.

- Atteintes cutanées et sous-cutanées : l'alopécie est très fréquente et réversible. Des réactions cutanées d'intensité modérée et peu fréquentes ont été rapportées.

- Troubles du système immunitaire : des réactions allergiques d'intensité modérée et peu fréquentes et de rares cas de réactions de type anaphylactique/anaphylactoïde ont été rapportés.

- Troubles musculosquelettiques : des effets précoces tels que contraction musculaire ou crampes et paresthésies ont été rapportés.

Examens biologiques : En monothérapie, une augmentation transitoire mineure à modérée des taux sériques soit des transaminases, soit des phosphatases alcalines, soit de la bilirubine est observée respectivement chez 9,2 %, 8,1 % et 1,8 % des patients, en l'absence de progression des métastases hépatiques. Une augmentation transitoire mineure à modérée des taux sériques de créatinine est observée chez 7,3 % des patients. En association, une élévation sérique transitoire (grade 1 et 2) des ALAT, ASAT, phosphatases alcalines ou bilirubine a été observée chez 15 %, 11 %, 11 % et 10 % des patients respectivement, en l'absence de progression des métastases hépatiques. Des grades 3 transitoires ont été observés chez 0 %, 0 %, 0 % et 1 % des patients respectivement. Aucun grade 4 n'a été observé. Dans de très rares cas, une élévation de l'amylase sérique et/ou de la lipase sérique a été rapportée. De rares cas d'hypokaliémie principalement liée à une diarrhée et des vomissements ont été rapportés. *Troubles du système nerveux :* de très rares cas de troubles du langage transitoires ont été rapportés lors de perfusions de Campto.

Extrait du RCP 5 Fluorouracile-

DC/EFFETS INDÉSIRABLES

Stomatite, Inflammation muqueuse, Diarrhée, Anorexie, Nausée, Vomissement, Hémorragie digestive (Exceptionnel), Coloration de la peau, Alopécie, Dermatite, Eruption cutanée, Urticaire, Photosensibilisation, Douleur précordiale, Electrocardiogramme(anomalie), Infarctus du myocarde (Exceptionnel), Leucopénie, Thrombopénie, Anémie (Rare), Ataxie, Larmoiement

Extrait du RCP de l'acide folinique

DC/EFFETS INDÉSIRABLES

Ils sont fonction de la dose et du schéma d'administration du 5-fluorouracile :

diarrhée : chez le sujet âgé, risque de déshydratation (cf Mises en garde/Précautions d'emploi) ; mucite, stomatite (cf Mises en garde/Précautions d'emploi) ; réactions cutanées : sécheresse de la peau, érythème ; conjonctivite, larmoiement ; toxicité hématologique modérée.

Extrait du RCP gemcitabine – GEMZAR®

DC/EFFETS INDÉSIRABLES

- Hématologiques : La gemcitabine peut induire une aplasie médullaire, entraînant une anémie, une leucopénie et une thrombocytopénie. La myélosuppression est généralement modérée, elle est plus prononcée pour la lignée granulocytaire. La thrombocytémie est un autre effet fréquemment rapporté.

- Hépatiques : Des augmentations des transaminases hépatiques sont observées. Elles sont habituellement faibles, transitoires et ne nécessitent que rarement l'arrêt du traitement. La prudence s'impose toutefois chez les patients dont la fonction hépatique est altérée.

- Œso-gastro-intestinaux : Nausées, parfois accompagnées de vomissements. Ces effets secondaires justifient des mesures thérapeutiques dans approximativement 20 % des cas, mais n'imposent que rarement la diminution de la dose et sont faciles à traiter par les antiémétiques classiques. Diarrhées, toxicité buccale à type de mucite.

- Pulmonaires : Dans les heures qui suivent l'injection de gemcitabine, les patients peuvent présenter une dyspnée, qui est généralement d'intensité faible et de courte durée. Elle nécessite rarement une réduction de

la posologie et disparaît habituellement sans traitement spécifique. Son mécanisme est inconnu et sa relation avec la gemcitabine n'est pas claire. Des cas d'œdème pulmonaire, de pneumopathies interstitielles et de syndrome de détresse respiratoire de l'adulte (ARDS), d'étiologie inconnue, ont été rapportés au cours du traitement par gemcitabine. Dès leur survenue, l'arrêt de la gemcitabine doit être envisagé.

- Rénaux : Une protéinurie et une hématurie modérées surviennent chez près de la moitié des patients, mais sont rarement significatives sur le plan clinique ; elles ne sont habituellement pas associées à des modifications de la créatinine sérique ou de l'urémie. On a cependant rapporté quelques cas d'insuffisance rénale d'étiologie incertaine. Aucune toxicité rénale cumulative n'a été observée (cf Mises en garde/Précautions d'emploi).Des manifestations cliniques compatibles avec un syndrome hémolytique et urémique ont été rapportées chez les patients recevant de la gemcitabine. Le traitement par gemcitabine doit être interrompu dès les premiers signes d'anémie hémolytique micro-angiopathique tels qu'une chute brutale de l'hémoglobine avec thrombocytopénie concomitante, élévation de la bilirubine sérique, de la créatinine sérique, de l'urée sanguine ou de la LDH. L'insuffisance rénale peut ne pas être réversible, même à l'arrêt du traitement, et une dialyse peut être nécessaire.

- Allergiques : Des éruptions peuvent survenir et s'accompagner de prurit. L'éruption est habituellement faible, ne nécessite pas de réduction posologique et répond à un traitement local. Une desquamation, une vésiculation et une ulcération sont des effets secondaires rapportés occasionnellement. Un bronchospasme a parfois été rapporté. Ce bronchospasme est habituellement d'intensité modérée et passager, mais il peut requérir un traitement parentéral. La gemcitabine ne doit pas être administrée aux patients ayant une hypersensibilité connue à ce produit. De rares cas de réaction anaphylactique ont été rapportés.

- Cardiaques : Des cas d'infarctus du myocarde, d'insuffisance cardiaque congestive et d'arythmie ont été observés. On a rapporté quelques cas d'hypotension.

- Cutanés : Des manifestations cutanéomusculaires sévères à type de dermatopolymyosite, au niveau du site antérieurement irradié, ont été rapportées après administration successive de radiothérapie et de gemcitabine.

- Autres : Un syndrome grippal rarement sévère peut survenir. Il est généralement de courte durée et nécessite rarement une diminution de la posologie. Fièvre, céphalées, dorsalgie, frissons, myalgies, asthénie et anorexie sont les symptômes les plus communément rapportés. De même, une toux, une rhinite, des malaises, des sueurs et une insomnie sont couramment signalés. La fièvre et l'asthénie sont également rapportées comme symptômes isolés. Le mécanisme à la base de cette toxicité est inconnu. Le paracétamol peut en atténuer les symptômes.
Œdème périphérique, très rarement œdème facial. L'œdème périphérique est habituellement modéré et n'impose que rarement une réduction de la posologie, mais peut être douloureux ; il est généralement réversible après l'arrêt de la gemcitabine. Le mécanisme à la base de cette toxicité est inconnu. Il n'y a aucune association avec des signes d'insuffisance cardiaque, hépatique ou rénale. Les effets secondaires suivants sont aussi couramment rapportés : alopécie (en général minime), somnolence.

Unité de Biostatistique
CTD labellisé INCa

Plan d'Analyse Statistique (Phase III)

PROTOCOLE ACCORD 11/0402

N° EudraCT : 2004-001985-42

ETUDE RANDOMISEE DE PHASE II/III COMPARANT L'ASSOCIATION FOLFIRINOX [OXALIPLATINE / IRINOTECAN / LV5FU] A LA GEMCITABINE EN PREMIERE LIGNE DE CHIMIOTHERAPIE DE PATIENTS ATTEINTS D'UN CANCER DU PANCREAS METASTATIQUE

Version 1, 03/07/2009

Promoteur :
FNCLCC
101, rue de Tolbiac
75654 Paris cedex 13, France

Investigateur principal :
Pr Thierry Conroy
Centre Alexis Vautrin
Département d'Oncologie médicale
6, avenue de Bourgogne
54511 Vandoeuvre les Nancy
Tel :03.83.59.84.60

Biostatisticien :
Sophie Gourgou-Bourgade
Tel :04.67.61.37.75 – Fax :04.67.61.37.18
E-Mail : sophie.gourgou@valdorel.fnclcc.fr

**ACCORD 11/0402**	
TITRE	ETUDE RANDOMISEE DE PHASE II/III COMPARANT L'ASSOCIATION FOLFIRINOX [OXALIPLATINE / IRINOTECAN / LV5FU] A LA GEMCITABINE EN PREMIERE LIGNE DE CHIMIOTHERAPIE DE PATIENTS ATTEINTS D'UN CANCER DU PANCREAS METASTATIQUE
Promoteur	FNCLCC
Auteur	Sophie Gourgou-Bourgade<br>+33 467 61 37 75 Fax : +33 467 61 37 18<br>E-mail : sophie.gourgou@valdorel.fnclcc.fr<br><br>CRLC Val d'Aurelle - Unité de Biostatistique<br>Parc Euromédecine<br>34298 MONTPELLIER Cedex 5 - FRANCE
Validé par	Andrew Kramar
Version	1
Date	10 juillet 2009

Page de SIGNATURES

J'ai lu ce plan d'analyse statistique et confirme que tous les objectifs d'étude comme indiqués dans le protocole sont couverts.
.

Sophie Gourgou-Bourgade		
Senior Biostatisticien<br>CRLC Val d'Aurelle	Signature	Date

Andrew Kramar		
Senior Biostatisticien<br>CRLC Val d'Aurelle	Signature	Date

Christine Montoto-Grillot		
Chef de projet<br>FNCLCC, Promoteur	Signature	Date

TABLE DES MATIERES

1 RATIONEL **6**

2 OBJECTIFS DE L'ETUDE **6**

3 METHODOLOGIE DE L'ETUDE **6**

3.1 DESCRIPTION 6
3.2 PLAN DE L'ÉTUDE 7
3.2.1 Durée de l'étude *7*
3.2.2 Traitement *7*
3.2.3 Méthode d'allocation des traitements *7*
3.3 COMITÉ DE SURVEILLANCE - DATA MONITORING COMMITTEE (DMC) 7
3.4 DÉCISION DE LA PHASE III 8
3.5 ANALYSE INTERMEDIAIRE AU COURS DE LA PHASE III 8

4 DETERMINATION DU NOMBRE DE SUJETS NECESSAIRES **8**

4.1 PHASE II 8
4.2 PHASE III 8

5 EFFICACITE ET PARAMATRES DE TOLERANCE **9**

5.1 CRITÈRE PRINCIPAL 9
5.2 CRITÈRES SECONDAIRES 9
5.3 PARAMÈTRES DE TOLÉRANCE 9

6 DEFINITION DES POPULATIONS **9**

7 ANALYSES STATISTIQUES **10**

7.1 MÉTHODES STATISTIQUES 10
7.1.1 Statistiques descriptives *10*
7.1.2 Variables qualitatives *10*
7.1.3 Données de Survie *10*
7.2 DÉFINITIONS AND CONVENTIONS 11
7.2.1 Definitions *11*
7.2.2 Conventions *11*
7.2.3 Données manquantes *11*
7.2.4 Dates manquantes ou incomplètes *11*
7.3 CARACTÉRISTIQUES DES PATIENTS 11
7.3.1 Disposition des patients *11*
7.3.2 Déviations au protocole *12*
7.3.3 Facteurs de la randomisation *12*
7.3.4 Caractéristiques démographiques *12*
7.3.5 Examens physiques et QLQ-C30 ..... *12*
7.3.6 Caractéristiques de la maladie *12*
7.3.7 Biologie initiale et marqueurs tumoraux *13*
7.4 TRAITEMENTS ÉTUDIÉS 13
7.4.1 Bras A : FOLFIRINOX *13*
7.4.2 Bras B : Gemcitabine *13*
7.5 EFFICACITÉ 14
7.5.1 Survie globale (critère principal) *14*
7.5.2 Survie sans progression *14*
7.5.3 Taux de réponse objective et durée de la réponse *14*
7.6 TOLÉRANCE 15
7.6.1 Exposition au traitement *15*
7.6.2 Evénements indésirables (EI) *17*
7.6.3 Données biologiques *17*
7.6.4 Etat général OMS *17*
7.6.5 Poids *17*
7.6.6 Traitements concomitants *17*
7.7 QUALITÉ DE VIE 18

8 REFERENCES **18**

LISTE DES ABBREVIATIONS ET DEFINITION DES TERMES

Abbréviations	Définition
EI	Evénements intercurrents (AE=adverse event)
ALAT	Alanine amino transferase
PAL	Phosphatase Alkaline
ASAT	Aspartate amino transferase
BSA / SC	Body Surface Area / Surface corporelle
SC / BSA	Surface corporelle / Body Surface Area
CRF	Case Report Form
DI	Dose Intensity / Dose intensité
DMC	Data Monitoring Committee
ECG	Electrocardiogramme
EORTC	European Organisation for Research and Treatment of Cancer
ICH	International Conference on Harmonisation
iDMC	Independent Data Monitoring Committee
ITT	Intention–to-treat / Intention de Traiter
NCI-CTC	National Cancer Institute - Common Toxicity Criteria
QLQ-C30	Quality of Life Questionnaire – C30
QLQ-PAN24	Quality of Life Questionnaire – PAN24
RDI	Relative Dose Intensity / Dose intensité relative
PAS	Plan d'Analyse Statistique

1 RATIONEL

Le plan d'analyses statistiques (PAS) décrit les analyses statistiques à réaliser pour l'étude ACCORD 11/0402-Prodige 4 concernant la partie phase III.

Un bref descriptif de la planification de l'étude sera réalisé, suivi par le détail des populations d'analyse et des méthodes statistiques utilisés. Les tables et listings à produire seront détaillés

Ce PAS a été rédigé à partir des documents suivants :

- Protocole original contenant l'ensemble des amendements (version 9).
- International Conference on Harmonisation (ICH) guideline E9 (Statistical Principles for Clinical Trials).

2 OBJECTIFS DE L'ETUDE

L'objectif principal de l'étude est d'évaluer l'efficacité en terme de survie globale de l'association oxaliplatine, irinotecan, 5 fluorouracile et acide folinique (Folfirinox – bras A) dans le traitement du cancer du pancréas métastatique par rapport au traitement de référence par gemcitabine (bras B).

Phase III:

Objectif principal:

- Comparer la survie globale globale entre les deux schémas thérapeutiques, l'association Folfirinox – bras A par rapport au traitement de référence par gemcitabine bras B.

Objectifs secondaires:

- Comparer la survie sans progression entre les 2 bras
- Comparer la qualité de vie entre les 2 bras.
- Comparer les taux de réponse
- Comparer la toxicité aux traitements
- Evaluer la qualité de vie selon les questionnaires EORTC QLQ-C30 et PAN 24.

3 METHODOLOGIE DE L'ETUDE

3.1 Description

Cet essai est une phase II/III, multicentrique, randomisé.

Quatre vingt huit patients ayant un cancer du pancréas métastatique seront randomisés dans l'étude de phase II. A la fin de la phase II, si la décision de continuer en phase III est prise, 272 patients supplémentaires seront randomisés en phase III pour atteindre un total de 360 patients

Les patients seront randomisés 1 :1 selon les deux bras de traitements suivants :

- **Bras A : Folfirinox** (oxaliplatine (Eloxatine®) 85 mg/m2 J1 en 2h, puis irinotecan (Campto®)180 mg/m2 J1 en 90mn + acide folinique 400 mg/m2, J1 en 2h (pendant la perfusion d'irinotécan) + 5-FU bolus 400 mg/m² J1 suivi de 5-FU continu 2,4 g/m2 au total sur 46 heures, soit 1,2 g/m2 à J1 et 1,2 g/m2 à J2).

- **Bras B : gemcitabine** (1000 mg/m2 en 30 mn par voie intra veineuse stricte, à J1, J8, J15, J22, J29, J36, J43 ; Reprise de la gemcitabine à J57, 3 semaines sur 4 (J57, J64, J71 suivi d'une semaine de pause)).

3.2 Plan de l'étude

3.2.1 Durée de l'étude

Les patients inclus dans le bras A (Folfirinox) seront traités jusqu'à la progression et un maximum de 12 cycles est conseillé.

Les patients inclus dans le bras B (gemcitabine) seront traités jusqu'à la progression et un maximum de 6 mois est conseillé.

Au delà de cette période de traitement protocolaire, la prise en charge médicamenteuse ou non du patient, est laissé au libre choix de l'investigateur.

De même en cas de progression, la prise en charge thérapeutique du patient est laissé au choix de l'investigateur.

L'ensemble des patients seront suivis jusqu'au décès.

3.2.2 Traitement

- **Bras A : Folfirinox**
  - oxaliplatine (Eloxatine®)85 mg/m2 J1 en 2h,
  - irinotecan (Campto®)180 mg/m^2 J1 en 90mn,
  - acide folinique 400 mg/m2, J1 en 2h (pendant la perfusion d'irinotécan),
  - 5-FU bolus 400 mg/m² J1 suivi de 5-FU continu 2,4 g/m2 au total sur 46 heures, soit 1,2 g/m2 à J1 et 1,2 g/m2 à J2).

- **Bras B : gemcitabine**
  - gemcitabine (1000 mg/m2 en 30 mn par voie intra veineuse stricte, à J1, J8, J15, J22, J29, J36, J43 ; Reprise de la gemcitabine à J57, 3 semaines sur 4 (J57, J64, J71 suivi d'une semaine de pause)).

3.2.3 Méthode d'allocation des traitements

Une randomisation centralisée (Sous Oracle Forms par Euraxi pharma) a été réalisée selon la méthode de minimisation selon les facteurs de stratification suivants :

- l'état général OMS (0 vs 1),
- la localisation (tête vs autre).
- le centre (n modalités),

Les patients seront randomisés selon selon un ratio 1:1 dans les deux bras de traitement :
Bras A: Folfirinox
Bras B: gemcitabine.

3.3 Comité de surveillance - Data Monitoring Committee (DMC)

Un DMC indépendant (iDMC) a été constitué afin de suivre l'évolution des inclusions dans l'étude, la tolérance et l'efficacité des traitements administrés. L'iDMC, est constitué de 2 oncologues médicaux, 2 radiothérapeutes et un statisticien ne participant pas à l'étude.

L'iDMC sera sollicité pour chacune des étapes des analyses intermédiaires planifiées.

3.4 Décision de la Phase III

A la fin de la phase II, il a été décide de continuer les inclusions en phase III randomisée dont l'objectif principal est d'évaluer la survie globale.

3.5 Analyse intermédiaire au cours de la Phase III

Une analyse intermédiaire d'efficacité sera réalisée lorsque 2/3 des événements (décès) auront été observés soit **167 événements**.
Afin de maintenir un risque global de 5%, cette analyse intermédiaire ne sera considérée comme significative que si la p-value est inférieure ou égale à 0,001 et **p≤0,049** à l'analyse finale.

Le Comité de Surveillance (iDMC) pourra proposer l'arrêt prématuré de l'essai s'il le juge nécessaire et si l'ensemble des données disponibles provenant de l'essai ou d'autres sources est suffisamment convaincant pour influencer les pratiques thérapeutiques de la majorité des médecins.

4 DETERMINATION DU NOMBRE DE SUJETS NECESSAIRES

4.1 Phase II

Un total de 88 patients devait être inclus dans la phase II (44 par bras). En réalité, 97 patients ont été inclus et seront utilisés dans l'analyse de phase III.

4.2 Phase III

Pour mettre en évidence une différence dans les médianes de survie de 3 mois (passage de 7 à 10 mois de médiane), soit un risque relatif de 0.70, il faudra inclure **360 patients** pour maintenir un risque global alpha de 5 %, en acceptant un risque beta de 20 % (puissance de l'essai = 80%).
Cette puissance sera obtenue lorsque **250 événements** auront été observés (calcul effectué avec le logiciel East 5).

Etant donné que 97 patients ont été inclus au cours de la phase II, 263 patients restent à être inclus au cours de la phase III pour atteindre les 360 patients nécessaires.

5 EFFICACITE ET PARAMATRES DE TOLERANCE

5.1 Critère principal

Le critère principal est la **survie globale** étant calculée depuis la date de randomisation et la survenue du décès. Les patients vivants aux dernières nouvelles seront censurés.

5.2 Critères secondaires

Les critères secondaires sont les suivants :

- **Survie sans progression** étant le délai entre la date de randomisation à la date de première mise en évidence d'une progression documentée, la date du décès, ou la date des dernières nouvelles. Les patients vivants sans rechute seront censurés.
- **Taux de réponse** (selon les critères RECIST) : sera prise en compte la meilleure réponse enregistrée entre le début du traitement et la progression de la maladie ou la dernière évaluation (meilleure réponse globale optimale). La réponse est définie selon les critères RECIST. Toutes les réponses objectives doivent être confirmées 4 semaines après leur observation par un nouvel examen. Un comité indépendant de 2 radiologues experts relira toutes les évaluations tumorales radiologiques pour confirmer les réponses enregistrées.
- **Durée de la réponse globale** étant calculée depuis la date à laquelle les critères de mesure définissant une réponse partielle ou complète sont rapportés jusqu'à la date de la documentation objective de la première progression,
- **Incidence de la toxicité** (selon l'échelle de toxicité NCI version 3.0),
- **Evaluation de la qualité de vie** selon le questionnaire EORTC QLQ-C30 (version 3) et le module spécifique EORTC-PAN24.

5.3 Paramètres de tolérance

Les paramètres de tolérance évalués seront l'ensemble des informations recueillies dans le cahier d'observation selon l'échelle CTC-NCI v3.0 après réconciliation avec les données de pharmacovigilance du promoteur.

6 DEFINITION DES POPULATIONS

<u>Population ITT :</u> l'ensemble des patients randomisés dans le bras de traitement (intention de traiter).

<u>Population évaluable pour la tolérance :</u> l'ensemble des patients randomisés ayant reçu au moins une cure ou une injection de traitement.

<u>Population éligible</u> : l'ensemble des patients randomisés ayant reçu au moins une dose de traitement et sans violation des critères d'inclusion ou de non inclusion.

<u>Population évaluable pour la réponse tumorale :</u>
À définir cf. PAS phase II

Une analyse en ITT sera réalisée sur l'ensemble des critères de jugement.

7 ANALYSES STATISTIQUES

7.1 Méthodes statistiques

7.1.1 Statistiques descriptives

Les variables quantitatives seront décrites par le nombre d'observations, la médiane, le minimum, et le maximum. Sauf indication contraire, la médiane sera présentée avec une décimale en plus de la valeur mesurée.
Les variables qualitatives seront décrites par le nombre d'observations (N) et la fréquence (%) avec le pourcentage par rapport à la population totale. Les catégories manquantes seront rajoutées pour rapporter l'ensemble des données. Les pourcentages seront présentés avec une décimale.
Les analyses descriptives seront résumées par bras de traitement et globalement seulement pour les caractéristiques initiales.

La "p-value" sera présentée avec 3 décimales sauf indication contraire.
Les tables et listings seront générées par STATA v10.0.

7.1.2 Variables qualitatives

Le test du Chi-2 sera utilisé pour comparer les proportions (ou test exact de Fisher si les fréquences attendues <5).
Les intervalles de confiance à 95% pour les proportions seront calculés par la méthode exacte Binomiale.

7.1.3 Données de Survie

Les données de survie correspondent à l'observation du délai pour qu'un événement particulier se produise (par exemple le temps jusqu'au décès).

La méthode de Kaplan-Meier sera utilisée pour analyser les données de survie et pour estimer les temps médian de survie. Les courbes de survie de Kaplan-Meier seront présentées. L'intervalle de confiance à 95% pour a médiane de survie sera calculé selon la méthode de Brookmeyer and Crowley (1982).
Les distributions de survie seront comparées par le test du logrank.

Les HRs et leurs IC95% seront estimés par un modèle à hazard proportionnel de Cox ajusté sur les facteurs de stratification utilisés pour la randomisation (OMS, localisation, centre). Le modèle de Cox sera utilisé pour comparer les distributions de survie après ajustement des éventuels facteurs pronostiques.

La validité des risques proportionnels sera évaluée graphiquement et/ou testée pour les covariables dépendantes du temps. Si une non proportionnalité des risques existe, les distributions de survie seront comparées par le test modifié de Kolmogorov-Smirnov,et ceci sera considéré comme support pour les comparaisons prévues par le test du logrank.

7.2 Définitions and conventions

7.2.1 Definitions

Age sera calculé ainsi :
Age = int((date at screening visit – date of birth) / 365.25)

Surface corporelle sera calculée selon la formule de Dubois and Dubois [2]
BSA/SC = (weight in kg) $^{0.425}$ *** (height in m)** $^{0.725}$ *** 0.20247**

Indice de Masse Corporelle sera calculé ainsi :
IMC= weight/ (height in m)²
Puis **IMC_cl** selon 4 catégories : maigres <18,5 / normaux<25 / surpoids<30 / obèses≥30

Jour 1 du cycle :
Le J1 du cycle est défini comme la date de 1ère administration du cycle.

Arrêt du traitement :
Les patients sont considérés sous traitement pour la durée du traitement et pour les 30 jours suivants l'arrêt du traitement.
L'arrêt du traitement est défini comme le dernier jour où le sujet reçoit un traitement à l'étude.

7.2.2 Conventions

Les délais jusqu'aux événements seront calculés à partir de la date de randomisation.
Pour tout calcul de délai ou de durée entre deux dates, la convention suivante sera appliquée : **[later date] – [earlier date] + 1 jour**.
Pour convertir un nombre de jours en année ou en mois, la convention suivante sera appliquée : **1 an = 365.25 jours**; **1 mois = 30.4375 jours**.

7.2.3 Données manquantes

Sauf indication contraire, les valeurs manquantes ne seront pas estimées.

7.2.4 Dates manquantes ou incomplètes

De la part d'EURAXI, convention lors de la 1ère analyse pour l'iDMC de décembre 2008 :
Si le jour d'une date est manquant, alors le jour central du mois (15) est attribué.

7.3 Caractéristiques des patients

Sur la population ITT

7.3.1 Disposition des patients

Les éléments suivants seront résumés par bras de traitement:

- Disposition des patients: population ITT (sujets randomisés), population évaluable pour la tolérance, population éligible, population évaluable pour la réponse tumorale.
- Administration des traitements et raison d'arrêt

Les listings suivants seront établis comme support :

- Sujets exclus de la population évaluable pour la tolérance (Sujet non traité)

- Sujets non éligibles
- Sujets non évaluables pour la réponse tumorale

7.3.2 Déviations au protocole

Suite à l'iDMC de décembre 2008, la notion de déviation majeure ou mineure est inutile étant donné que l'ensemble des analyses seront réalisée en ITT.

7.3.3 Facteurs de la randomisation

Les facteurs relevés au moment de la randomisation seront décrits :

- Sexe: homme/femme : fréquence et %
- Age (années) : médiane et étendue
- Etat général OMS : fréquence et %
- Localisation tumorale : fréquence et %

7.3.4 Caractéristiques démographiques

Les caractéristiques démographiques seront résumées selon les informations de la randomisation et du bilan d'inclusion.

- Sexe: homme/femme : fréquence et %
- Age (années) : médiane et étendue
- Etat général OMS : fréquence et %
- Poids (kg) : médiane et étendue
- Taille (cm) : médiane et étendue
- Surface corporelle (m^2) : médiane et étendue
- IMC (kg/m^2) sera calculé : médiane et étendue

Les groupes seront comparés pour évaluer la comparabilité initiale sur ces caractéristiques.

7.3.5 Examens physiques et QLQ-C30

Les examens réalisés à l'inclusion seront décrits :

- Scanner thoraco-abdominopelvien: délai par rapport à la randomisation, fréquence et % du résultat normal/anormal,
- ECG : délai par rapport à la randomisation, fréquence et % du résultat normal/anormal,
- Test de grossesse chez les femmes : délai par rapport à la randomisation, fréquence et % du résultat positif/négatif.
- Qualité de vie QLQ-C30 : score initiaux des 8 dimensions évaluées / médiane et étendue

7.3.6 Caractéristiques de la maladie

Les groupes seront comparés pour évaluer la comparabilité initiale sur ces caractéristiques.

7.3.6.1 Tumeur primitive

Les caractéristiques de la tumeur primitive seront résumées selon les informations du bilan d'inclusion (CRF p3).

- Délai du diagnostic à partir de la randomisation
- Type de diagnostic, différenciation
- Délai Randomisation/évaluation tumorale

- Type d'examen, classification pTNM, cTNM
- Localisation de la tumeur primitive
- Dimension maximale de la tumeur
- Traitements antérieurs éventuels (naute, délais par rapport à la randomisation)

7.3.6.2 Tumeur métastatique

Les caractéristiques de la tumeur métastatique seront résumées selon les informations du bilan d'inclusion (CRF p3).

- Délai du diagnostic à partir de la randomisation
- Type de diagnostic, différenciation
- Délai Randomisation/évaluation tumorale
- Type d'examen, classification pTNM, cTNM
- Traitements antérieurs éventuels (naute, délais par rapport à la randomisation)
- Marqueurs tumoraux (CA19-9, ACE)
- Nombre de sites métastatiques
- Nature des sites métastatiques
- Nombre de lésions mesurables et non mesurables et sites

7.3.7 Biologie initiale et marqueurs tumoraux

Les résultats biologiques initiaux suivants ont été identifies comme « Non fait »/ « normaux »/ « anormaux » et seront décrit tels quels :

- Bilan hématologique: Hémoglobine, neutrophiles/granulocytes, plaquettes, TP et autres hématologie.
- Bilan biochimique: Calcium, Glycémie, albumine, protidémie, autre
- Bilan hépatique : bilirubine totale/libre, LDH, Phosphatase alkaline (PAL), ALAT (SGPT), ASAT (SGOT), Gamma GT, autre
- Bilan rénal : Créatininémie, autre.

7.4 Traitements étudiés

7.4.1 Bras A : FOLFIRINOX

Seront décrit pour chaque drogue :

- Le nombre de cycles administrés,
- La dose cumulée (mg/m²)
- La dose intensité (mg/m²/semaine)
- La dose intensité relative
- Modification : Nombre de réductions, d'arrêts et raisons
- Retards des administrations et raisons

7.4.2 Bras B : Gemcitabine

Seront décrit :

- Le nombre de cycles administrés, de perfusion
- La dose cumulée (mg/m²)
- La dose intensité (mg/m²/semaine)
- La dose intensité relative
- Modification : Nombre de réductions, d'arrêts et raisons
- Retards des administrations et raisons

7.5 Efficacité

7.5.1 Survie globale (critère principal)

Population: ITT

Globalement et par bras de traitement, seront données :

- Le suivi médian,
- La survie globale médiane,
- Les taux de survie globale à 6 et 12 mois,
- Le nombre d'événements observés,
- Le nombre de sujets à risque
- Le test du logrank associé
- Le modèle de Cox stratifié sur les facteurs de stratification (HR et IC95%) univarié et multivarié,
- Les courbes de survie globale.

7.5.2 Survie sans progression

Population: ITT

Globalement et par bras de traitement, seront données :

- La survie sans progression médiane,
- Les taux de survie sans progression,
- La nature et le nombre d'événements observés,
- Le nombre de sujets à risque
- Le test du logrank associé
- Le modèle de Cox stratifié sur les facteurs de stratification (HR et IC95%) univarié et multivarié,
- Les courbes de survie sans progression.

7.5.3 Taux de réponse objective et durée de la réponse

Population: ITT, Population évaluable pour la réponse

La réponse tumorale a été évaluée selon les critères RECIST.

Le taux de réponse globale de la 1ère évaluation sera décrit par bras de traitement par la fréquence de chaque type de réponse, le % et les intervalles de confiance à 95%.

Le meilleur taux de réponse globale sur l'ensemble des évaluations sera décrit par la fréquence de chaque type de réponse et son % et l'intervalle de confiance à 95% associé pour chaque bras de traitement.
Le test de chi-2 ou de Fisher's Exact si approprié seront utilisés pour comparer les bras de traitement en terme de taux de réponse.

La durée de la réponse médiane sera estimée par la méthode de Kaplan-Meier.

7.6 Tolérance

7.6.1 Exposition au traitement

L'exposition aux traitements sera évaluée par la description des doses cumulées, dose intensité et dose intensité relative de chacune des drogue par patient et par bras de traitement.

La dose cumulée (mg/m²) :
La dose cumulée (mg/m²) par patient est la somme des doses totales administrées que le sujet à reçu au cours de l'étude rapporté à la surface corporelle (Somme doses (mg)/ SC(m²)).

7.6.1.1 5-FU

Les patients inclus dans le bras A FOLFIRINOX auront reçu un bolus de 400mg/m² plus une perfusion de 46 heures en continu de 2,4 g/m² pour chaque cycle (soit 1,2 g/m² à J1 et 1,2 g/m² à J2) après oxaliplatine, acide folinique et irinotecan.

La durée de traitement par 5-FU (en semaines) au cours de l'étude est définie comme : **[last dosing date – first dosing date + 13]/7**

La dose intensité et la dose intensité relative par patient seront calculées pour des cycles de 2 semaines.
La dose intensité (mg/m²/cycle) est définie comme :

$$\frac{\text{Dose cumulée de 5-FU (mg/m}^2\text{)}}{\text{Durée de traitement par 5-FU (en semaines) / 2}}$$

La dose intensité relative est définie comme la dose intensité divisée par la dose prévue par cycle (2800 mg/m²/2 semaines=cycle).

7.6.1.2 Irinotecan (iri)

Les patients inclus dans le bras A FOLFIRINOX auront reçu une dose de 180 mg/m² IV en perfusion de 90 minutes après l'oxaliplatine.

La durée de traitement par l'irinotecan (en semaines) au cours de l'étude est définie comme : **[last dosing date – first dosing date + 14]/7**

La dose intensité et la dose intensité relative par patient seront calculées pour des cycles de 2 semaines.

La dose intensité (mg/m²/cycle) est définie comme :

$$\frac{\text{Dose cumulée de IRI (mg/m}^2\text{)}}{\text{Durée de traitement par IRI (en semaines) / 2}}$$

La dose intensité relative est définie comme la dose intensité divisée par la dose prévue par cycle (180 mg/m²/2 semaines=cycle).

7.6.1.3 Oxaliplatin (oxa)

Les patients inclus dans le bras A FOLFIRINOX auront reçu une dose de 85 mg/m² IV en perfusion de 2 heures à J1 avant toute autre perfusion toutes les 2 semaines.

La durée de traitement par oxaliplatin (en semaines) au cours de l'étude est définie comme : **[last dosing date – first dosing date + 14]/7**

La dose intensité et la dose intensité relative par patient seront calculées pour des cycles de 2 semaines.

La dose intensité (mg/m²/cycle) est définie comme :

$$\frac{\text{Cumulative dose of Oxaliplatin (mg/m²)}}{\text{Durée de traitement par oxaliplatin (en semaines) / 2}}$$

La dose intensité relative est définie comme la dose intensité divisée par la dose prévue par cycle (85 mg/m²/2 semaines=cycle) i.e.

$$\text{RDI} = \frac{\text{Dose Intensité}}{\text{Dose prévue}} = \frac{\dfrac{\text{Dose cumulée d'Oxaliplatin}}{\text{Durée de trt par oxaliplatin (sem) / 2}}}{\text{Dose prévue (mg/m²/cycle)}}$$

Ce qui peut être écrit comme :

$$\frac{\dfrac{\text{Dose cumulée d'Oxaliplatin}}{\text{[last dosing date – first dosing date + 14]/7}}}{\text{Planned dose * } \dfrac{\text{Nbre de cycles}}{\text{14*nbre de cycles /7}}}$$

7.6.1.4 Gemcitabine (gem)

Les patients inclus dans le bras B Gemcitabine auront reçu une dose de 1000 mg/m² en perfusion de 30 minutes apr IV stricte une fois par semaine pendant 7 semaines suivie de 14 jours de repos. Puis à partir du cycle suivant, l'administration est répétée une fois par semaine pendant 3 semaines consécutives, suivie d'une semaine de repos.

La durée de traitement par gemcitabine (en semaines) au cours de l'étude est définie comme : **[last dosing date – first dosing date + 7]/7**

La dose intensité et la dose intensité relative par patient seront calculées par semaine.

La dose intensité (mg/m²/semaine) est définie comme :

$$\frac{\text{Dose cumulée de gemcitabine (mg/m²)}}{\text{Durée de traitement par gemcitabine (en semaines)}}$$

La dose intensité relative est définie comme la dose intensité divisée par la dose prévue par semaine (1000 mg/m²/semaines).

7.6.2 Evénements indésirables (EI)

Population évaluable pour la tolérance

Tous les événements indésirables liés aux traitements ont été recueillis seront décrits selon les groupes prédéfinis dans le CRF.
La gravité des EI sera grade selon l'échelle de toxicité du NCI-CTC (version 3.0).

Les EI seront décrits pour chaque bras de traitement par cycle et par patient.
Chaque EI sera décrit selon les 5 grades (0,1,2,3,4) et selon les grades 3/4.

Pour l'analyse par patient, si un EI est rapporté plus d'une fois au cours du traitement, le grade le plus sévère sera rapporté pour le patient.
Pour l'analyse par cycle, si un EI est rapporté plus d'une fois au cours d'un cycle de traitement, le grade le plus sévère du cycle sera rapporté pour le patient.

Le test de chi-2 ou de Fisher's Exact si approprié seront utilisés pour comparer les bras de traitement en terme d'incidence de toxicité de grade 3/4.

7.6.3 Données biologiques

Les données biologiques ont été recueillies au cours des cycles selon une cotation normale/anormale.
Elles seront décrites telles quelles pour chaque bras de traitement par cycle et par patient.
Pour l'analyse par patient, si une donnée biologique est rapportée anormale au moins une fois au cours du traitement, cette donnée sera rapporté pour le patient.
Pour l'analyse par cycle, si une donnée biologique est rapportée anormale au moins une fois au cours du cycle, cette donnée sera rapporté pour le patient.

7.6.4 Etat général OMS

L'évolution de l'état général OMS depuis le bilan d'inclusion jusqu'à la fin de l'étude sera présentée par bras de traitement.

7.6.5 Poids

Le poids étant mesuré à chaque cycle, l'évolution de ce paramètre depuis le bilan d'inclusion jusqu'à la valeur la plus basse après la 1ère dose de drogue sera codé selon l'échelle de toxicité CTC-NCI version 3.
L'incidence du pire changement de grade par patient sera décrit par bras de traitement. La perte et le gain de poids seront décrits.

Paramètre	*Grade 0*	*Grade 1*	*Grade 2*	*Grade 3*	*Grade 4*
Gain/perte de poids	<5%	5 – <10%	10 – <20%	≥20%	--

7.6.6 Traitements concomitants

La prise de traitements concomitants et leur durée au cours des cycles seront décrites et listés par bras de traitement.

7.7 Qualité de Vie

La Qualité de vie sera évaluée par le questionnaire EORTC QLQ-C30 et le module spécifique validé EORTC PAN 24.

8 REFERENCES

[1] Haybittle JL: Repeated assessments of results in clinical trials of cancer treatment. Br J Radiol 1971;44:793-7

[2] DuBois D; DuBois EF: A formula to estimate the approximate surface area in height and weight be known. *Arch Int Med* 1916 17:863-71
